# Green Synthesis of Thiazolidine-2,4-dione Derivatives and Their Lipoxygenase Inhibition Activity With QSAR and Molecular Docking Studies

**DOI:** 10.3389/fchem.2022.912822

**Published:** 2022-07-05

**Authors:** Melita Lončarić, Ivica Strelec, Valentina Pavić, Vesna Rastija, Maja Karnaš, Maja Molnar

**Affiliations:** ^1^ Department of Applied Chemistry and Ecology, Faculty of Food Technology Osijek, Josip Juraj Strossmayer University of Osijek, Osijek, Croatia; ^2^ Department of Biology, Josip Juraj Strossmayer University of Osijek, Osijek, Croatia; ^3^ Department of Agroecology and Environmental Protection, Faculty of Agrobiotechnical Sciences Osijek, Josip Juraj Strossmayer University of Osijek, Osijek, Croatia

**Keywords:** 2,4-thiazolidinedione, synthesis, deep eutectic solvents, green chemistry, lipoxygenase, QSAR, molecular docking

## Abstract

Thiazolidinediones are five-membered, heterocyclic compounds that possess a number of pharmacological activities such as antihyperglycemic, antitumor, antiarthritic, anti-inflammatory, and antimicrobial. Conventional methods for their synthesis are often environmentally unacceptable due to the utilization of various catalysts and organic solvents. In this study, deep eutectic solvents were used in the synthesis of thiazolidinedione derivatives that acted as both solvents and catalysts. Initially, a screening of 20 choline chloride-based deep eutectic solvents for thiazolidinedione synthesis, *via* Knoevenagel condensation, was performed in order to find the most suitable solvent. Deep eutectic solvent, choline chloride, *N*-methylurea, was proven to be the best for further synthesis of 19 thiazolidinedione derivatives. Synthesized thiazolidinediones are obtained in yields from 21.49% to 90.90%. The synthesized compounds were tested for the inhibition of lipid peroxidation as well as for the inhibition of soy lipoxygenase enzyme activity. The antioxidant activity of the compounds was also determined by the ABTS and DPPH methods. Compounds showed lipoxygenase inhibition in the range from 7.7% to 76.3%. Quantitative structure–activity relationship model (*R* ^2^ = 0.88; *Q* ^*2*^_loo_ = 0.77; *F* = 33.69) for the inhibition of soybean lipoxygenase was obtained with descriptors *Mor29m, G2u*, and *MAXDP*. The molecular docking confirms experimentally obtained results, finding the binding affinity and interactions with the active sites of soybean LOX-3.

## 1 Introduction

Thiazolidine-2,4-dione is a five-membered, heterocyclic compound. Chemically, thiazole ring contains the carbonyl moiety at positions 2 and 4, the -NH group, and the methylene group (–CH_2_), which allows for various modifications of the molecule. Thiazolidine-2,4-dione derivatives are important heterocyclic compounds that possess a number of pharmacological activities such as antihyperglycemic, antitumor (Chadha et al., 2015), antiarthritic (da Rocha Junior et al., 2013), anti-inflammatory (Youssef et al., 2010), and antimicrobial (Albrecht et al., 2005). It is used in the production of drugs for diabetes mellitus (type 2) treatment. It belongs to the so-called glitazone drugs such as rosiglitazone, pioglitazone, lobeglitazone, and troglitazone (Chadha et al., 2015). Thiazolidinedione is used to inhibit metal corrosion in acidic solutions and as a reagent sensitive to heavy metals in analytical chemistry (Jain et al., 2013). Thiazolidinedione derivatives can inhibit certain enzymes such as aldose reductase, phosphoinositide-3-kinase, Pim kinase, cyclooxygenase, D-glutamate ligase, and histone deacetylase (Beharry et al., 2009; Jain et al., 2013; Kim et al., 2013). Some thiazolidinedione derivatives also showed good potential for lipoxygenase inhibition (Bozdag-Dündar et al., 2009; Avupati et al., 2018).

Lipoxygenases are enzymes that belong to dioxygenases containing iron and catalyze the oxidation of polyunsaturated fatty acids (Ivanov et al., 2010; Kavetsou et al., 2017). These enzymes are present in plants, mammals, and microorganisms (Sadeghian and Jabbari, 2016). Four seed isoforms of lipoxygenases (LOX-1, LOX-2, LOX-3a, LOX-3b) have been identified in soybean (*Glycine max*) (Axelrod et al., 1981). Due to the lack of other sufficient purified isoforms, soybean LOX-3 has been the most investigated isoform (Jothi et al., 2018). In lipoxygenase pathway, undesirable compounds may be formed causing side effects in plants and vegetables by changing color, creating unwanted odors, and changing the antioxidant properties (Chatterjee and Sharma, 2018). Some natural compounds, such as polyphenols (-)-epigallocatechin gallate (Skrzypczak-Jankun et al., 2003a), curcumin (Skrzypczak-Jankun et al., 2003b), quercetin (Sadik et al., 2003), and coumarins (Torres et al., 2013) can effectively inhibit this enzyme. Previous studies have shown that various synthesized organic compounds can inhibit lipoxygenase: thiazolyl derivatives (Tsolaki et al., 2018) and coumarin derivatives (Lončarić et al., 2020). The X-ray structures of soybean LOX-3 complexes with inhibitors such as 4-nitrocatechol (PDB ID: 1NO3) (Skrzypczak-Jankun et al., 2004) and protocatechuic acid (PDB ID: 1N8Q) (Borbulevych et al., 2004) were revealed providing a deeper insight into the mode of inhibition.

Organic compounds are still often synthesized by conventional synthetic procedures, including the usage of volatile organic solvents and harmful catalysts, showing adverse environmental and health effects. Following the new trends and concepts in green chemistry, the utilization of such harmful chemicals is to be avoided or at least minimized. A pursuit of environmentally acceptable solvents has led us to the utilization of deep eutectic solvents (DESs). These solvents are characterized by low vapor pressure, non-flammability, and easy handling, in addition to being biodegradable, do not require any purification before use, and can be recycled and reused (Zhang et al., 2012).

The thiazolidinediones used in this research are synthesized in deep eutectic solvents without the usage of any organic solvents and catalysts. In this research, the inhibition of lipid peroxidation and lipoxygenase activity of thiazolidinedione derivatives were tested. In addition, the antioxidant activity of the examined thiazolidinediones was determined by DPPH and ABTS methods. The quantitative structure–activity relationship (QSAR) study was performed to reveal significant structural characteristics important for lipoxygenase inhibition. Molecular docking of the analyzed compounds to the soybean lipoxygenase (LOX)-3 was evaluated in order to compare the binding affinities with experimentally determined inhibitions and determined the interactions with the binding site of the enzyme.

## 2 Materials and Methods

All chemicals used within this study were purchased from commercial suppliers. For thin-layer chromatography (TLC), fluorescent silica gel plates F254 (Merck, Darmstadt, Germany) were used, and TLC was performed in benzene:acetone:acetic acid (8:1:1) as an eluent. Determination of melting points of compounds was conducted on the electrothermal melting point apparatus (Electrothermal Engineering Ltd., Rochford, United Kingdom). Mass spectra were recorded on an LC/MS/MS API 2000 sp (CA, United States). NMR spectra were recorded on a Bruker Avance 600 MHz NMR Spectrometer (Bruker Biospin GmbH, Rheinstetten, Germany) at 293 K in dimethylsulfoxide-*d*6 (DMSO-*d*6). Spectrophotometric analysis were performed on ThermoSpectronic, Helios Gamma spectrophotometer (Thermo Fisher Scientific, Waltham, MA, United States).

### 2.1 General Procedure for the Synthesis of Deep Eutectic Solvents

Deep eutectic solvents (DESs) were obtained by mixing hydrogen bond acceptor (HBA) (choline chloride) and various hydrogen bond donors (HBDs) in molar ratios, as listed in Table 1. Choline chloride was mixed with appropriate HBDs, and the mixture was heated until a clear liquid was obtained.

**TABLE 1:** Reaction times and product yields obtained in different deep eutectic solvents.

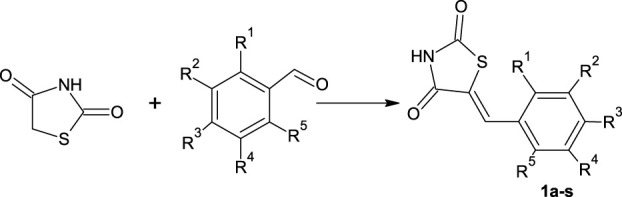				
HBA	HBD	Ratio ChCl:HBD	Time (h)	Y (%)
ChCl	Urea	1:2	2.5	22.8
	*N*-Methylurea	1:3	2	79.9
	Thiourea	1:2	6	6.4
	Glucose	1:1	9	17.9
	Fructose	1:1	8	14.1
	Xylitol	1:1	10	^a^
	Sorbitol	1:1	10	^a^
	Butan-1,4-diole	1:2	10	^a^
	Ethan-1,2-diole	1:2	10	^a^
	Glycerol	1:2	6	25.2
	Acetamide	1:2	10	^a^
	Malic acid	1:1	10	^a^
	Citric acid	1:2	10	^a^
	Tartaric acid	1:1	10	^a^
	Malonic acid	1:1	10	^a^
	Oxalic acid	1:1	10	^a^
	1,3-Dimethylurea	1:2	8	3.8
	Lactic acid	1:2	10	^a^
	Levulinic acid	1:2	10	^a^
	*trans*-Cinnamic acid	1:1	10	^a^

a No product obtained.

### 2.2 General Procedure for the Synthesis of Thiazolidinedione Derivatives (1a–s)

Thiazolidinedione derivatives were synthesized by the Knoevenagel condensation. An equimolar amount of thiazolidine-2,4-dione and substituted benzaldehyde was added to the DES and stirred until completion of the reaction monitored by TLC. Upon the consumption of the reactants, water was added, and the precipitated product was filtered. Detailed procedure for each synthesized compound, as well as compound characterization, is given in Section 2.4.

#### 2.2.1 General Procedure for the Recyclability of the DES

Recyclability experiments were performed on the model reaction in choline chloride: *N*-methylurea DES. After the reaction was finished, the addition of water precipitated the crude product, which was filtered off. Then, the water was evaporated from the mixture and the same reaction was conducted again, under the same conditions. This was repeated five times.

### 2.3 Inhibitory Activities

Inhibitory activities are measured in the presence of thiazolidinedione derivatives dissolved in DMSO at a concentration of 100 µM. The soybean lipoxygenase inhibition assay, the inhibition of linoleic acid lipid peroxidation, and the reducing activity of the stable radical 1,1-diphenyl-picrylhydrazyl (DPPH) were determined, as described previously (Lončarić et al., 2020).

The ABTS^•+^ method was performed according to the previously published procedures with several modifications (Osman et al., 2012). Solutions of the ABTS radical (7.4 mM) and potassium persulfate (2.6 mM) in water were mixed in a molar ratio of 1:1. The prepared mixture was left in the dark place at room temperature for 16 h. Thereafter, the prepared solution was diluted with DMSO to achieve a solution absorbance of 1.0 ± 0.02 at 734 nm. All synthesized compounds were dissolved in DMSO and prepared at a concentration of 1 mM. One hundred microliters of a compound solution in DMSO and 900 µl of ABTS^•+^ were mixed and allowed to stand for 90 min in a dark place to allow the reaction to proceed. The absorbance of the mixture was measured at 734 nm. The study was conducted in three parallels.

### 2.4 Compound Characterization (1a–s)

#### 2.4.1 5-(2-Hydroxybenzylidene)thiazolidine-2,4-dione (1a)

Using thiazolidinedione (0.234 g, 2 mmol) and salicylaldehyde (215 µl, 2 mmol), in accordance with the general procedure, the title compound **1a** was obtained (0.159 g, 35.9% yield) as a yellow solid (m.p. 269–272°C). ^**1**^ **H** (600 MHz) δ 12.48 (s, 1H, NH), 10.48 (s, 1H, OH), 8.00 (s, 1H, CH), 7.30 (q, *J* = 7.92; 9.84; 7.26 Hz, 2H, arom.), 6.93 (q, *J* = 8.40; 9.48; 7.50 Hz, 2H, arom.). ^**13**^ **C** (150 MHz) δ 168.15; 167.50; 157.24; 132.20; 128.20; 126.27; 121.87; 119.64; 116.08. LC/MS (C_10_H_7_NO_3_S): *m/z*: 220.01 (M-) 221.23.

#### 2.4.2 5-(4-Hydroxy-3-methoxybenzylidene)thiazolidine-2,4-dione (1b)

Using thiazolidinedione (0.234 g, 2 mmol) and 4-hydroxy-3-methoxybenzaldehyde (0.304 g, 2 mmol), in accordance with the general procedure, the title compound **1b** was obtained (0.343 g, 68.3% yield) as a brown solid (m.p. 199–201°C). ^**1**^ **H** (300 MHz) δ 11.98 (s, 1H, NH), 9.94 (s, 1H, OH), 7.71 (s, 1H, CH), 7.17 (d, *J* = 1.89 Hz, 1H, arom.), 7.07 (dd, *J* = 8.34; 1.89 Hz, 1H, arom.), 6.93 (d, *J* = 8.25 Hz, 1H, arom.), 3.82 (s, 3H, OCH_3_). ^**13**^ **C** (150 MHz) δ 172.47; 168.52; 167.94; 149.89; 148.43.130.06; 124.85; 124.59; 119.68; 116.71; 114.60. LC/MS (C_11_H_9_NO_4_S): *m/z*: 250.06 (M-) 251.26.

#### 2.4.3 5-(2,5-Dihydroxybenzylidene)thiazolidine-2,4-dione (1c)

Using thiazolidinedione (0.234 g, 2 mmol) and 2,5-dihydroxybenzaldehyde (0.276 g, 2 mmol), in accordance with the general procedure, the title compound **1c** was obtained (0.102 g, 21.5% yield) as a brown solid (m.p. 194–198°C). ^**1**^ **H** (600 MHz) δ 9.70 (s, 1H, OH), 7.61 (s, 1H, CH), 7.23 (d, *J* = 8.76 Hz, 1H, arom.), 6.98 (s, 1H, arom.), 6.92 (dd, *J* = 8.22; 1.68; 2.40 Hz, 1H, arom.). ^**13**^ **C** (150 MHz) δ 153.98; 144.08; 132.23; 128.61; 120.04; 117.85; 116.75; 110.91. LC/MS (C_10_H_7_NO_4_S): *m/z*: 258.82 (M-/+Na) 237.23.

#### 2.4.4 5-(3-Methoxybenzylidene)thiazolidine-2,4-dione (1d)

Using thiazolidinedione (0.234 g, 2 mmol) and 3-methoxybenzaldehyde (245 µl, 2 mmol), in accordance with the general procedure, the title compound **1d** was obtained (0.178 g, 37.8% yield) as a white solid (m.p. 194–197°C). ^**1**^ **H** (300 MHz) δ 12.62 (s, 1H, NH), 7.76 (s, 1H, CH), 7.45 (t, *J* = 8.16 Hz, 1H, arom.), 7.15 (d, *J* = 6.45 Hz, 2H, arom.), 7.06 (dd, *J* = 8.34; 1.89; 0.30 Hz, 1H, arom.), 3.80 (s, 3H, OCH_3_). ^**13**^ **C** (150 MHz) δ 168.28; 167.70; 160.08; 134.85; 132.21; 130.86; 122.36; 116.74; 115.76; 55.73. LC/MS (C_11_H_9_NO_3_S): *m/z*: 233.99 (M-) 235.26.

#### 2.4.5 5-(3-Hydroxy-4-methoxybenzylidene)thiazolidine-2,4-dione (1e)

Using thiazolidinedione (0.234 g, 2 mmol) and 3-hydroxy-4-methoxybenzaldehyde (0.304 g, 2 mmol), in accordance with the general procedure, the title compound **1e** was obtained (0.271 g, 53.9% yield) as a yellow solid (m.p. 254–257°C). ^**1**^ **H** (600 MHz) δ 12.46 (s, 1H, NH), 9.47 (s, 1H, OH), 7.62 (s, 1H, CH), 7.06 (d, *J* = 2.16 Hz, 2H, arom.), 7.00 (d, *J* = 1.56 Hz, 1H, arom.), 3.81 (s, 3H, OCH_3_). ^**13**^ **C** (150 MHz) δ 168.03; 167.40; 149.99; 146.89; 132.24; 125.63; 123.45; 119.96; 115.84; 112.37; 55.63. LC/MS (C_11_H_9_NO_4_S): *m/z*: 250.00 (M-) 251.26.

#### 2.4.6 5-(3,4-Dihydroxybenzylidene)thiazolidine-2,4-dione (1f)

Using thiazolidinedione (0.234 g, 2 mmol) and 3,4-dihydroxybenzaldehyde (0.276 g, 2 mmol), in accordance with the general procedure, the title compound **1f** was obtained (0.139 g, 29.3% yield) as a brown solid (m.p. 270–271°C). ^**1**^ **H** (600 MHz) δ 12.42 (s, 1H, NH), 9.82 (s, 1H, OH), 9.44 (s, 1H, OH), 7.60 (s, 1H, CH), 6.99 (d, *J* = 1.98 Hz, 1H, arom.), 6.96 (q, *J* = 8.28; 1.95 Hz, 1H, arom.), 6.87 (d, *J* = 8.22 Hz, 1H, arom.). ^**13**^ **C** (150 MHz) δ 168.15; 166.53; 148.57.145.82; 132.63; 124.28; 123.89; 118.72; 116.37; 116.22. LC/MS (C_10_H_7_NO_4_S): *m/z*: 236.01 (M-) 237.23.

#### 2.4.7 5-(3,4,5-Trimethoxybenzylidene)thiazolidine-2,4-dione (1g)

Using thiazolidinedione (0.234 g, 2 mmol) and 3,4,5-trimethoxybenzaldehyde (0.392 g, 2 mmol), in accordance with the general procedure, the title compound **1g** was obtained (0.341 g, 57.8% yield) as a yellow solid (m.p. 172–174°C). ^**1**^ **H** (600 MHz) δ 12.59 (s, 1H, NH), 7.72 (s, 1H, CH), 6.88 (s, 2H, arom.), 3.80 (s, 6H, OCH_3_), 3.70 (s, 3H, OCH_3_). ^**13**^ **C** (150 MHz) δ 167.81; 167.20.155.15; 139.35; 132.05; 128.49; 122.41; 107.48; 60.16; 55.96. LC/MS (C_13_H_13_NO_5_S): *m/z*: 294.11 (M-) 295.31.

#### 2.4.8 5-(2,5-Dimethoxybenzylidene)thiazolidine-2,4-dione (1h)

Using thiazolidinedione (0.234 g, 2 mmol) and 2,5-dimethoxybenzaldehyde (0.332 g, 2 mmol), in accordance with the general procedure, the title compound **1h** was obtained (0.432 g, 81.3% yield) as a yellow solid (m.p. 220–223°C). ^**1**^ **H** (600 MHz) δ 12.56 (s, 1H, NH), 7.90 (s, 1H, CH), 7.07 (t, *J* = 2.64 Hz, 2H, arom.), 6.89 (d, *J* = 2.40 Hz, 1H, arom.), 3.82 (s, 3H, OCH_3_), 3.74 (s, 3H, OCH_3_). ^**13**^ **C** (150 MHz) δ 167.91; 167.28; 153.01; 152.36; 126.35; 123.79; 121.90; 117.57.113.22; 113.00; 56.06: 55.47. LC/MS (C_12_H_11_NO_4_S): *m/z*: 263.99 (M-) 265.29.

#### 2.4.9 5-(3-Bromobenzylidene)thiazolidine-2,4-dione (1i)

Using thiazolidinedione (0.234 g, 2 mmol) and 3-bromobenzaldehyde (235 µl, 2 mmol), in accordance with the general procedure, the title compound **1i** was obtained (0.397 g, 69.8% yield) as a white solid (m.p. 210–212°C). ^**1**^ **H** (300 MHz) δ 12.69 (s, 1H, NH), 7.82 (t, *J* = 1.56 Hz, 1H, arom.), 7.78 (s, 1H, CH), 7.68 (m, 1H, arom.), 7.59 (d, *J* = 7.86 Hz, 1H, arom.), 7.50 (t, *J* = 7.82 Hz, 1H, arom.). ^**13**^ **C** (150 MHz) δ 168.05; 167.68; 135.98; 133.31; 133.23; 131.79; 130.46; 128.55; 125.92; 122.90. LC/MS (C_10_H_6_BrNO_3_S): *m/z*: 284.03 (M-) 284.13.

#### 2.4.10 5-(2-Hydroxy-5-nitrobenzylidene)thiazolidine-2,4-dione (1j)

Using thiazolidinedione (0.234 g, 2 mmol) and 5-nitrosalicylaldehyde (0.334 g, 2 mmol), in accordance with the general procedure, the title compound **1j** was obtained (0.334 g, 62.8% yield) as a red solid (m.p. 226–228°C). ^**1**^ **H** (600 MHz) δ 8.40 (s, 1H, OH), 8.17 (d, *J* = 2.82 Hz, 1H, arom.), 8.04 (dd, *J* = 9.24; 2.82 Hz, 1H, arom.), 7.85 (s, 1H, CH), 6.80 (d, *J* = 9.18 Hz, 1H, arom.). ^**13**^ **C** (150 MHz) δ 169.06; 127.19; 125.27; 125.07; 121.01; 117.88. LC/MS (C_10_H_6_N_2_O_5_S): *m/z*: 265.04 (M-) 266.23.

#### 2.4.11 5-(2-Methoxybenzylidene)thiazolidine-2,4-dione (1k)

Using thiazolidinedione (0.234 g, 2 mmol) and 2-methoxybenzaldehyde (0.241 µl, 2 mmol), in accordance with the general procedure, the title compound **1k** was obtained (0.334 g, 71.0% yield) as a yellow solid (m.p. 240–241°C). ^**1**^ **H** (600 MHz) δ 12.54 (s, 1H, NH), 7.95 (s, 1H, CH), 7.45–7.48 (m, 1H, arom.), 7.39 (dd, *J* = 7.74; 1.38 Hz, 1H, arom.), 7.14 (d, *J* = 8.22 Hz, 1H, arom.), 7.08 (t, *J* = 7.53 Hz, 1H, arom.), 3.87 (s, 3H, OCH_3_). ^**13**^ **C** (150 MHz) δ 168.09; 167.41; 132.36; 128.52; 126.42; 123.43; 121.41.120.90; 111.82; 55.73. LC/MS (C_11_H_9_NO_3_S): *m/z*: 233.84 (M-) 235.26.

#### 2.4.12 5-(3-Hydroxybenzylidene)thiazolidine-2,4-dione (1l)

Using thiazolidinedione (0.234 g, 2 mmol) and 3-hydroxybenzaldehyde (0.244 g, 2 mmol), in accordance with the general procedure, the title compound **1l** was obtained (0.132 g, 29.7% yield) as a brown solid (m.p. 262–264°C). ^**1**^ **H** (300 MHz) δ 12.58 (s, 1H, NH), 9.82 (s, 1H, OH), 7.67 (s, 1H, CH), 7.31 (t, *J* = 7.89 Hz, 1H, arom.), 7.01 (d, *J* = 7.89 Hz, 1H, arom.), 6.96 (s, 1H, arom.), 6.86 (dd, *J* = 8.07; 1.76 Hz, 1H, arom.). ^**13**^ **C** (150 MHz) δ 168.42; 167.80; 158.32; 134.65; 132.45.130.83; 123.75; 121.78; 118.19; 116.37. LC/MS (C_10_H_7_NO_3_S): *m/z*: 220.50 (M-) 221.23.

#### 2.4.13 5-(4-Hydroxybenzylidene)thiazolidine-2,4-dione (1m)

Using thiazolidinedione (0.234 g, 2 mmol) and 4-hydroxybenzaldehyde (0.244 g, 2 mmol), in accordance with the general procedure, the title compound **1m** was obtained (0.269 g, 60.8% yield) as a yellow solid (m.p. 296–297°C). ^**1**^ **H** (600 MHz) δ 12.43 (s, 1H, NH), 10.30 (s, 1H, OH), 7.69 (s, 1H, CH), 7.45 (d, *J* = 8.58 Hz, 2H, arom.), 6.91 (d, *J* = 8.64 Hz, 2H, arom.). ^**13**^ **C** (150 MHz) δ 168.05; 167.52; 159.84; 132.35; 132.24; 123.90; 118.97; 116.28. LC/MS (C_10_H_7_NO_3_S): *m/z*: 220.11 (M-) 221.23.

#### 2.4.14 5-(4-(Dimethylamino)benzylidene)thiazolidine-2,4-dione (1n)

Using thiazolidinedione (0.234 g, 2 mmol) and 4-dimethylaminobenzaldehyde (0.298 g, 2 mmol), in accordance with the general procedure, the title compound **1n** was obtained (0.451 g, 90.9% yield) as an orange solid (m.p. 286–289°C). ^**1**^ **H** (600 MHz) δ 12.31 (s, 1H, NH), 7.66 (s, 1H, CH), 7.43 (d, *J* = 9.00 Hz, 2H, arom.), 6.82 (d, *J* = 9.00 Hz, 2H, arom.), 3.01 (s, 6H, CH_3_). ^**13**^ **C** (150 MHz) δ 168.15; 167.57; 151.39; 132.84; 132.08; 127.75; 119.79; 115.69; 111.99. LC/MS (C_12_H_12_N_2_O_2_S): *m/z*: 247.02 (M-) 248.30.

#### 2.4.15 5-(4-(Benzyloxy)-2-hydroxybenzylidene)thiazolidine-2,4-dione (1o)

Using thiazolidinedione (0.117 g, 1 mmol) and 4-(benzyloxy)salicylaldehyde (0.228 g, 1 mmol), in accordance with the general procedure, the title compound **1o** was obtained (0.291 g, 88.9% yield) as a yellow solid (m.p. 184–188°C). ^**1**^ **H** (600 MHz) δ 12.39 (s, 1H, NH), 10.54 (s, 1H, OH), 7.91 (s, 1H, CH), 7.44 (d, *J* = 8.46 Hz, 2H, arom), 7.40 (t, *J* = 7.47 Hz, 2H, arom.), 7.35 (m, 1H, arom.), 7.28 (d, *J* = 8.76 Hz, 1H, arom.), 6.65 (dd, *J* = 8.76; 2.46 Hz, 1H, arom.), 6.57 (d, *J* = 2.52 Hz, 1H, arom.), 5.11 (s, 2H, CH_2_). ^**13**^ **C** (150 MHz) δ 161.08; 158.70; 136.57; 129.40; 128.44; 127.92; 127.69; 113.99; 106.85; 102.01; 69.28; 55.11; 53.13. LC/MS (C_17_H_11_NO_4_S): *m/z*: 326.20 (M+) 325.34.

#### 2.4.16 5-(3-Fluorobenzylidene)thiazolidine-2,4-dione (1p)

Using thiazolidinedione (0.234 g, 2 mmol) and 3-fluorobenzaldehyde (212 µl, 2 mmol), in accordance with the general procedure, the title compound **1p** was obtained (0.140 g, 31.4% yield) as a white solid (m.p. 169–171°C). ^**1**^ **H** (600 MHz) δ 12.67 (s, 1H, NH), 7.78 (s, 1H, CH), 7.58 (q, *J* = 7.98; 6.18 Hz, 1H, arom.), 7.44 (dd, *J* = 9.96; 1.98 Hz, 1H, arom.), 7.42 (d, *J* = 7.92 Hz, 1H, arom.), 7.32 (ddd, *J* = 2.16; 8.46; 8.70 Hz, 1H, arom.). ^**13**^ **C** (150 MHz) δ 167.54; 167.11; 163.03; 161.41; 135.35; 131.29; 130.31; 125.50; 119.97; 116.58. LC/MS (C_10_H_6_FNO_2_S): *m/z*: 222.31 (M-) 223.22.

#### 2.4.17 5-(2,4-Dimethoxybenzylidene)thiazolidine-2,4-dione (1q)

Using thiazolidinedione (0.234 g, 2 mmol) and 2,4-dimethoxybenzaldehyde (0.332 g, 2 mmol), in accordance with the general procedure, the title compound **1q** was obtained (0.467 g, 87.9% yield) as a yellow solid (m.p. 251–253°C). ^**1**^ **H** (300 MHz) δ 12.42 (s, 1H, NH), 7.90 (s, 1H, CH), 7.33 (d, *J* = 8.40 Hz, 1H, arom.), 6.69 (dd, *J* = 11.61; 2.40 Hz, 2H, arom.), 3.87 (s, 3H, OCH_3_), 3.82 (s, 3H, OCH_3_). ^**13**^ **C** (150 MHz) δ 168.04; 163.52; 160.28; 130.52; 126.92; 120.34; 114.73; 106.74; 99.09; 56.38; 56.09. LC/MS (C_12_H_11_NO_4_S): *m/z*: 264.16 (M-) 265.29.

#### 2.4.18 5-Benzylidenethiazolidine-2,4-dione (1r)

Using thiazolidinedione (0.234 g, 2 mmol) and benzaldehyde (205 µl, 2 mmol), in accordance with the general procedure, the title compound **1r** was obtained (0.134 g, 32.7% yield) as a white solid (m.p. 251–254°C). ^**1**^ **H** (600 MHz) δ 12.62 (s, 1H, NH), 7.80 (s, 1H, CH), 7.52 (m, 6H, arom.). ^**13**^ **C** (150 MHz) δ 168.33; 167.77; 133.49; 132.24; 130.87; 130.46; 129.77; 124.01. LC/MS (C_10_H_7_NO_2_S): *m/z*: 204.22 (M-) 205.23.

#### 2.4.19 5-((1H-Indol-3-yl)methylene)thiazolidine-2,4-dione (1s)

Using thiazolidinedione (0.234 g, 2 mmol) and indole-3-carboxaldehyde (0.290 g, 2 mmol), in accordance with the general procedure, the title compound **1s** was obtained (0.439 g, 89.8% yield) as a yellow solid (m.p. >300°C). ^**1**^ **H** (600 MHz) δ 12.31 (s, 1H, NH), 12.12 (s, 1H, NH), 8.06 (s, 1H, CH), 7.88 (d, *J* = 7.86 Hz, 1H, arom.), 7.73 (d, *J* = 2.76 Hz, 1H, arom.), 7.51 (d, *J* = 8.04 Hz, 1H, arom.), 7.25 (t, *J* = 7.44 Hz, 1H, arom.), 7.19 (t, *J* = 7.38 Hz, 1H, arom.). ^**13**^ **C** (150 MHz) δ 167.23; 136.16; 128.58; 126.75; 124.41; 123.02; 121.00; 118.29; 116.20; 112.36; 110.39. LC/MS (C_12_H_8_N_2_O_2_S): *m/z*: 243.05 (M-) 244.27.

### 2.5 Computational Methods

#### 2.5.1 QSAR Study

The activities of molecules expressed as the % inhibition of soybean lipoxygenase (LOX inh. %) were converted to the logarithmic values (log % LOX inh.). The 3D structures of 19 molecules were optimized by applying Spartan ‘08 (Wavefunction, Inc., Irvine, CA, United States) using the molecular mechanics force field (MM+) (Hocquet and Langgård, 1998) and subsequently by the semiempirical PM3 method (Stewart, 1989). The molecular descriptors were generated using Parameter Client (Virtual Computational Chemistry Laboratory; http://146.107.217.178/lab/pclient/). The initial number of 1728 calculated descriptors was reduced on 568 exclusion of descriptors with zero and constant values, as descriptors that were too intercorrelated (>85%), using QSARINS-Chem 2.2.1 (University of Insubria, Varese, Italy). Generation of the QSAR model was performed using QSARINS with a genetic algorithm, limiting the number of descriptors in the model (I) to three. Considering a reduced number of compounds, the set of molecules was not split on training and test, and the models were assessed by fitting criteria and internal cross-validation (Gramatica, 2013). In order to assess the reliability of the prediction of the modeled inhibition for the entire set of chemicals, the investigation of the applicability domain was performed. Williams plots were used to check the outliers and molecules outside of warning leverage (h*), which is defined as 3*p*’/*n*, where n is the number of training compounds and *p*’ is the number of model-adjustable parameters (Gramatica, 2013).

#### 2.5.2 Molecular Docking

The crystal coordinates of the soybean LOX-3 (PDB ID: 1NO3) in the complex with ligand 4-nitrocatechol (PDB ID: 4NC) were downloaded from the Protein Data Bank (PDB; https://www.rcsb.org/). BIOVIA Discovery Studio 4.5 (Dassault Systèmes, France) was used for protein structure preparation and visualization, while iGEMDOCK (BioXGEM, Taiwan) was used for molecular docking. A set of the optimized structures of 19 thiazolidinediones were docked into the binding site of radius 8 Å, determined according to the ligand 4NC. Molecular docking was performed using the evolutionary method with parameters: the population size: 200, generations: 70, the number of poses: 3. iGEMDOCK generates protein–compound interaction profiles and rank compounds by pharmacological energy profiles mines the pharmacological interactions based on protein–compound interaction profiles. Pharmacological scoring function (*E*pharma/(kcal mol^−1^) is based on the pharmacological interactions (electrostatic (*E*), hydrogen-bonding (*H*), and van der Waals (*V*) (Hsu et al., 2011).

## 3 Results and Discussion

### 3.1 Synthesis of Thiazolidinedione Derivatives

**scheme 1:**
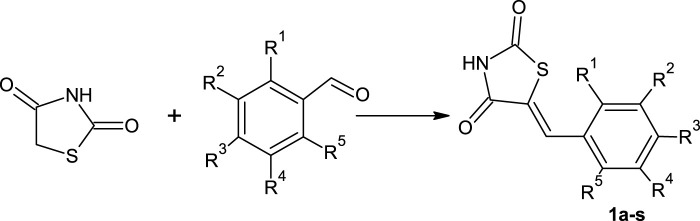
Synthesis of thiazolidinedione derivatives 1a-s.

A series of thiazolidinedione derivatives were synthesized in various DESs *via* Knoevenagel condensation. Thiazolidinedione derivatives were synthesized in reactions between different substituted benzaldehydes and thiazolidinedione (**1a**–**s**) (Scheme 1). The structures of all obtained thiazolidinedione derivatives were confirmed by different spectral methods (spectral data are shown in Supplementary Material S1).

In this research, a selected model reaction between 4-dimethylaminobenzaldehyde and thiazolidinedione was carried out in 20 different DESs listed in Table 1. Selected DESs were applied as both solvents and catalysts. Due to the high viscosity of some DESs or their melting points above room temperature, we chose a temperature of 80°C for all reactions. The product of model reaction (5-(4-(dimethylamino)benzylidene)thiazolidine-2,4-dione) was obtained in seven DESs in which the hydrogen bond donors were amides (urea, *N*-methylurea, 1,3-dimethylurea, acetamide), thioamide (thiourea), alcohol (ethane-1,2-diol), and carboxylic acid (oxalic acid) (Table 1). The lowest reaction yield of 3.8% was obtained in ChCl: oxalic acid DES. The highest yield of 79.9% was obtained in ChCl: *N*-methylurea DES, while other yields were significantly lower and ranged from 3.8 to 25.2%. The reaction time ranged from 2 to 9 h. The longest duration of 9 h was shown by the reaction that took place in the reaction medium ChCl: 1,3-dimethylurea. ChCl: *N*-methylurea DES was selected as the most suitable solvent for further synthesis of thiazolidinedione derivatives, in which, with the highest product yield, the reaction took place in the shortest time of 2 h. If the product did not appear at the TLC plate after 10 h, the reaction was stopped. The duration of the chemical reaction as well as all the yields obtained in the mentioned DESs are shown in Table 1.

**FIGURE 1:**
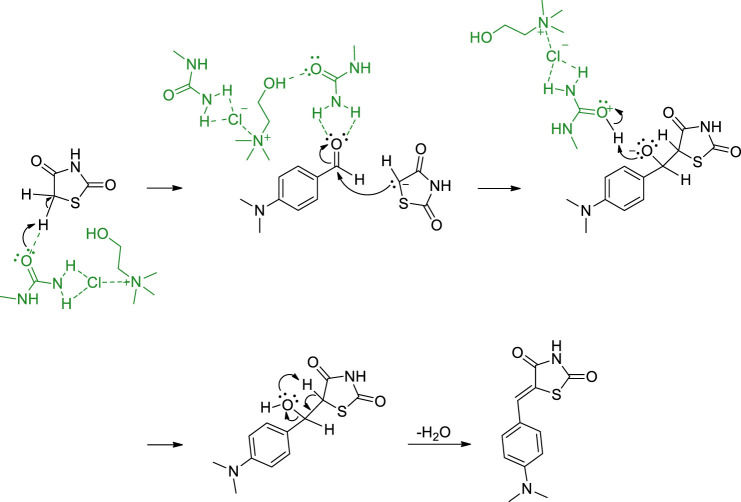
Proposed mechanism of Knoevenagel condensation for the synthesis of thiazolidinedione derivatives in DES..

The proposed mechanism of the model reaction with DES ChCl: *N*-methylurea is shown in Figure 1. In the first step of the reaction, thiazolidinedione is deprotonated and protonated *N*-methylurea is formed. The deprotonated thiazolidinedione is added to the carbonyl carbon of the aldehyde, while oxygen takes the proton from the DES, dehydration occurs and thiazolidinediones are formed.

After solvent selection, various substituted aldehydes were reacted with thiazolidinedione to obtain the desired thiazolidinedione derivatives. All reactions were monitored by TLC and quenched with water after the disappearance of reactants on the TLC plate. The precipitated product was collected by filtration. The yields of thiazolidinedione series of compounds ranged from 21.5% to 90.9% (Section 2.4). The lowest yield was obtained for compound **1c**, while the highest yield was observed for compound **1n**. The isolated compounds’ yield are highly dependent on the substituents on the aldehyde aromatic ring, which determine the aldehyde reactivity toward thiazolidinedione as well as the solubility of the synthesized compounds in water. Substituents on the aromatic ring can increase or decrease the positive character of carbonyl carbon in aldehydes, making them more or less susceptible to nucleophilic attack. Furthermore, they can also influence the compounds’ water solubility. Since all reactions within this research were performed until the full consumption of reactants, the product solubility in water could have determined the isolated yield. The best yields are obtained for aldehydes with electron-donating groups, such as -N(CH_3_)_2_ and -OCH_3_, while the lowest yields were obtained in the compounds containing –OH groups. As already mentioned, this effect is probably a result of the higher solubility of these compounds in water. Since a simple precipitation of the product was accomplished by the addition of water in the reaction mixture, some compounds possessing higher water solubility could have been obtained in lower yields. For the sake of greenness and simplicity of the method, further extractions of such compounds were not performed. Some of the synthesized compounds have already been synthesized and published by various authors. The yields obtained by some conventional methods for some thiazolidinedione derivatives were compared to those obtained by our method (Table 2). As can be seen, the yields of some compounds obtained within this research are comparable to the reported data, while for some compounds, lower yields are obtained. It is important to accentuate that out method does not utilize any catalysts, while for most compounds, a purification step is also avoided. To emphasize the green character of this reaction, we also performed a recyclability experiments on the model reaction in choline chloride: *N*-methylurea DES. The results (Table 3) indicate that after five cycles, a yield slightly dropped from 70.43% to 61.88%.

**TABLE 2:** Comparison to other reported methods of thiazolidinedione synthesis.

Compound	Yield obtained in this research (%)	Method	Yield (%)	References
**1a**	35.9	Reflux in ethanol; piperidine	37	Ha et al. (2012)
		Ethanol; baker’s yeast	40	Pratap et al. (2011)
		Tetrabutylammonium bromide; potassium carbonate	95	Durai Ananda Kumar et al. (2015)
		Water; KAl (SO_4_)_2_ ∙ 12H_2_O; stirring	88	Shelke et al. (2010)
**1e**	53.9	Reflux in ethanol; piperidine	53	Ha et al. (2012)
**1f**	29.3	Water; KAl (SO_4_)_2_ ∙ 12H_2_O; stirring	85	Shelke et al. (2010)
**1g**	57.8	Reflux in polyethylene glycol-300	78	Mahalle et al. (2008)
		Reflux in ethanol; piperidine	38	Ha et al. (2012)
**1h**	81.3	Tetrabutylammonium bromide; potassium carbonate	92	Durai Ananda Kumar et al. (2015)
**1l**	29.7	Tetrabutylammonium bromide; potassium carbonate	89	Durai Ananda Kumar et al. (2015)
**1m**	60.8	Mechanosynthesis; ammonium acetate	96	Metwally et al. (2011)
		Reflux in ethanol; piperidine	67	Ha et al. (2012)
		Ethanol: water (1:1); ionic liquid tetrabutylammonium hydroxide, stirring	92	Khazaei et al. (2014)
		Tetrabutylammonium bromide; potassium carbonate	91	Durai Ananda Kumar et al. (2015)
		Water; KAl (SO_4_)_2_ ∙ 12H_2_O; stirring	92	Shelke et al. (2010)
**1n**	90.9	Ethanol: water (1 : 1); ionic liquid tetrabutylammonium hydroxide, stirring	90	Khazaei et al. (2014)
		Tetrabutylammonium bromide; potassium carbonate	92	Durai Ananda Kumar et al. (2015)
		Water; KAl (SO_4_)_2_ ∙ 12H_2_O; stirring	87	Shelke et al. (2010)
**1q**	87.9	Reflux in ethanol; piperidine	96	Ha et al. (2012)
**1r**	32.7	Mechanosynthesis; ammonium acetate	96	Metwally et al. (2011)
		Ethanol: water (1 : 1); ionic liquid tetrabutylammonium hydroxide, stirring	90	Khazaei et al. (2014)
		Ethanol; baker’s yeast	45	Pratap et al. (2011)
		Tetrabutylammonium bromide; potassium carbonate	93	Durai Ananda Kumar et al. (2015)
		Water; KAl (SO_4_)_2_ ∙ 12H_2_O; stirring	95	Shelke et al. (2010)
**1s**	89.8	Reflux in toluene; L-proline	92	Riyaz et al. (2011)

**TABLE 3:** The impact of solvent recycling on product yield for model reaction.

Solvent	Yield (%)
Choline chloride: *N*-methylurea	70.43
1st recycle	75.53
2nd recycle	64.01
3rd recycle	62.25
4th recycle	63.35
5th recycle	61.88

### 3.2 Inhibitory Activity

The synthesized thiazolidinedione derivatives (**1a–s**) were tested for the inhibition of lipid peroxidation as well as the inhibitory activity against soybean lipoxygenase at a 100 µM concentration in the reaction mixture. All the results are shown in Table 4. Thiazolidinedione derivatives inhibited the LOX activity in the range of 7.3%–76.3%. Of the 19 compounds tested, only two inhibited the LOX activity above 50%. The two compounds with the highest activity are 5-(2,5-dihydroxybenzylidene)thiazolidine-2,4-dione (**1c**) and 5-((1*H*-indol-3-yl)methylene)thiazolidine-2,4-dione (**1s**). The determined IC_50_ values for compounds **1c** and **1s** were 3.52 and 7.46 µM, respectively. Compound 5-(3,4,5-trimethoxybenzylidene)thiazolidine-2,4-dione (**1g**), which has three methoxy groups substituted on the aromatic ring, had the lowest enzyme inhibition. Omar et al. (2020) carried out an evaluation of 31 thiazolidin-4-one-1,3,4-thiadiazole hybrids for their activity toward LOX. Results showed that increasing the length of the aliphatic substituent decreased the LOX inhibitory potency, while increasing the number of hydroxy groups decreased the inhibitory activity. Similar trends were observed for derivatives with methoxy groups. Their results showed that small hydrophobic group, at the 5th position of thiazolidinone, was required for good activity, while the 5th position of the thiazolidinone moiety should be unsubstituted. Marc et al. (2019) synthesized 12 new phenolic derivatives of thiazolidine-2,4-dione and found that in most cases, antioxidant activities were linked to the number of phenolic OH groups present in the molecules. Thiazolidinedione derivatives inhibited lipid peroxidation in the range of 23.0%–84.2%. The highest inhibition was achieved with the compound 5-(3-methoxybenzylidene)thiazolidine-2,4-dione (**1d**), while the lowest inhibition was achieved with the compound 5-(4-hydroxybenzylidene)thiazolidine-2,4-dione (**1m**). A high inhibition of lipid peroxidation of 82.9% was also shown by the compound 5-((1*H*-indol-3-yl)methylene)thiazolidine-2,4-dione (**1s**). Compounds with halogen substituents (**1i**) and (**1p**) showed similar lipid peroxidation inhibition values of 49.2% and 46.0%, respectively. All results were compared to appropriate standards. The results of the inhibition of the LOX activity were compared with the NDGA standard whose inhibition was 80.8%. The results of lipid peroxidation inhibition were compared with Trolox whose inhibition was 62.3%.

**TABLE 4:** Structures of analyzed compounds, values of the experimentally determined inhibition of soybean lipoxygenase, the inhibition of lipid peroxidation induced by AAPH, DPPH, and radical scavenging ability (at 100 μM concentrations of the compounds).

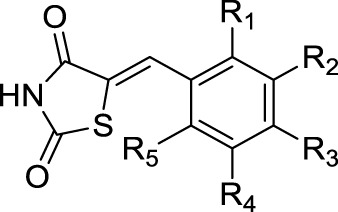										
Compound	R_1_	R_2_	R_3_	R_4_	R_5_	LP inh. (%) (100 μM)	LOX inh. % (100 μM)	LOX inh. IC_50_ (μM)	DPPH (%) (100 μM)	ABTS (%) (100 μM)
1a	OH	H	H	H	H	55.2	13.8	-	3.5	97.5
1b	H	OCH_3_	OH	H	H	36.5	8.5	-	27.9	99.7
1c	OH	H	H	OH	H	61.6	76.3	3.52	16.5	97.6
1d	H	OCH_3_	H	H	H	84.2	27.2	-	6.2	NA
1e	H	OH	OCH_3_	H	H	65.1	14.5	-	3.1	98.9
1f	H	OH	OH	H	H	50.4	13.4	-	57.6	100.0
1g	H	OCH_3_	OCH_3_	OCH_3_	H	46.3	7.3	-	3.3	NA
1h	OCH_3_	H	H	OCH_3_	H	62.4	17.6	-	4.2	NA
1i	H	Br	H	H	H	49.2	7.7	-	3.1	NA
1j	OH	H	H	NO_2_	H	76.9	18.3	-	9.6	49.3
1k	OCH_3_	H	H	H	H	55.9	30.31	-	3.8	NA
1l	H	OH	H	H	H	68.2	20.2	-	10.7	84.7
1m	H	H	OH	H	H	69.5	12.7	-	3.8	94.8
1n	H	H	NC_2_H_6_	H	H	23.0	12.5	-	7.6	74.7
1o	OH	H	OCH_2_PH	H	H	50.0	34.7	-	NA	100.0
1p	H	F	H	H	H	46.0	12.9	-	9.4	NA
1q	OCH_3_	H	OCH_3_	H	H	59.9	19.2	-	4.7	6.6
1r	H	H	H	H	H	56.7	18.8	-	3.6	NA
1s	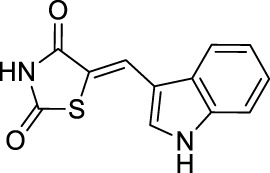					82.9	71.1	7.46	2.7	84.2
		Trolox				62.3	-	-	-	-
		NDGA				-	80.8	-	-	-

NA, no activity; NDGA, nordihydroguaiaretic acid; DPPH-1, 1-diphenyl-picrylhydrazyl; ABTS, 2,2′-azino-bis(3-ethylbenzothiazoline-6-sulfonic acid; LP, lipid peroxidation; LOX, inh., soybean lipoxygenase inhibition.

Thiazolidinedione derivatives inhibited ABTS radicals in the range of 6.6%–100.0% at a concentration of 100 µM (Table 4). High inhibition values (>94%) of the ABTS radical were shown by the following compounds: **1a**, **1b**, **1c**, **1e**, **1f**, **1m,** and **1o**, which had a substituted hydroxyl group on the phenyl ring. Compounds **1d**, **1g**, **1h**, **1i**, **1k**, **1p,** and **1r** did not show activity at the tested concentration and had one or more methoxy groups or a halogen element substituted on the benzene ring. Inhibition values of DPPH radical were 2.7%–57.6%. The compound 5-(3,4-dihydroxybenzylidene) thiazolidine-2,4-dione (**1f**) had the highest inhibition, while the compound 5-((1*H*-indol-3-yl) methylene)thiazolidine-2,4-dion (**1s**) had the lowest activity. Compound **1o** showed no activity. From the obtained results, it can be concluded that the compounds with a substituted hydroxyl group had high inhibition values of the ABTS radical, since the inhibition is achieved by donating a hydrogen atom. Inhibition values of the DPPH radical were much lower in comparison with the ABTS radical inhibition, perhaps because of a different reaction mechanism. It can be assumed that they react on the principle of the SET mechanism, and the tested compounds are not good electron donors (Shalaby and Shanab, 2013; Liang and Kitts, 2014; Li et al., 2018). It has already been shown that a catechol-like structure of a 3,4-dihydrohybenzilidene moiety greatly contributes to the antioxidant activity, both in ABTS (Kim et al., 2019; Ilyasov et al., 2020) and in DPPH scavenging activities (Molnar et al., 2012). Therefore, the presence of hydroxyl groups contributes to the increased antioxidant activity, while halogen, methoxy, phenoxy, and benzyloxy groups contribute to the increased inhibition of lipoxygenase activity and lipid peroxidation.

### 3.3 QSAR

Generation of QSAR models for the inhibition of soybean lipoxygenase was performed on the set of the 19 compounds. The data were not split into the training and test sets due to a limited number of compounds. The best QSAR model obtained is:

$$\begin{array}{c} \log\;\text{LOX}\;\text{inh}\text{.}\;\%=2.48+2.56Mor\mathrm{29}m+6.28G2u-0.53MAXDP\\ N=19 \end{array}$$ (1)

**FIGURE 2:**
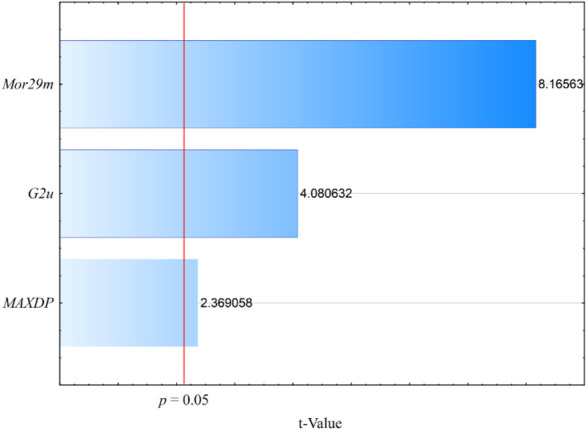
Chart of each descriptor t-values in the model (1).

**FIGURE 3:**
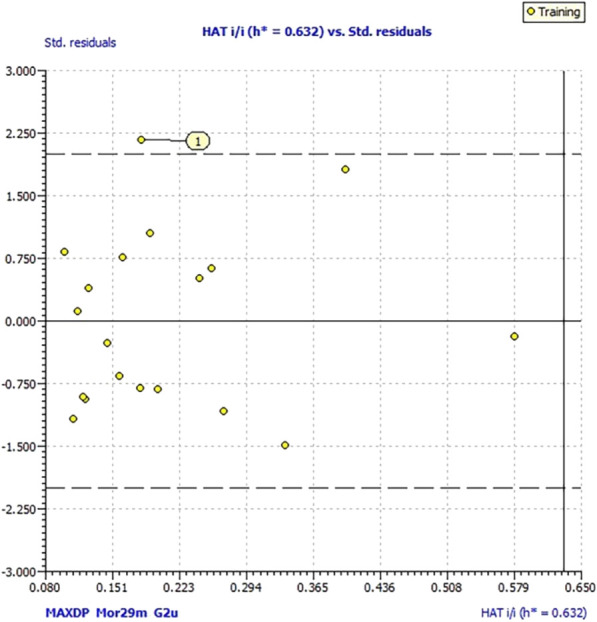
Williams plot of applicability domain of the QSAR model (1).

Descriptors in Eq. 1 are listed in order of relative importance by their *t* values (coefficient divided by its standard error) shown in the chart (Figure 2). The molecular descriptor values have been tabulated in Supplementary Material S2a. Experimental and calculated log LOX inh. % by model (1) are shown in Supplementary Material S2b. Values of Pearson correlation coefficient (*R* < 0.70) in the correlation matrix between the descriptors (Table 5) excluded their collinearity overfitting. Also, the strong correlation between the descriptor *Mor29m* and LOX inhibitions could be observed (*R* = 0.74). The parameters of fitting and internal validation for the QSAR model (1) are presented in Table 6. Low collinearity between the descriptors is confirmed by the low values of *Kxx* and *ΔK* > 0.05. Model satisfied all fitting parameters: *R* ^2^ ≥ 0.7; *R* ^2^–*R* ^2^_adj_ < 0.3; and *RMSE*_tr_ < 0.3; *CCC*_tr_ > 0.85. The model demonstrates a satisfactory stability in internal validation (*Q* ^*2*^_LOO_ ≥ 0.5; *Q* ^*2*^_LMO_ ≥ 0.6; *RMSE*_tr_ < *RMSE*_cv_; *r* ^2^_m_ ≥ 0.6) (Kiralj and Ferreira, 2009; Veerasamy et al., 2011). Parameters of Y-scrambling (*R* ^*2*^_Yscr_ < 0.2; *Q* ^*2*^_*Yscr*_ < 0.2) highlight that the model is robust and not obtained by chance correlation (Masand et al., 2019). The only failed parameter is the concordance correlation coefficient obtained by cross-validation (*CCC*_cv_) that is higher than 0.85. A lower value of this parameter indicates low reproducibility of cross-validation and thus lower accuracy of the proposed model (Lin, 1992). Inspection of the Williams plot of the applicability domain (Figure 3) revealed the one outlier, molecule **1a**, which has the greatest residual (0.25) between experimental and calculated values (Supplementary Material S2b). to measure the agreement. Molecules outside of the warning leverage (h*) were not identified. Exclusion of molecule **1a** from the data set, the following model was obtained using the same descriptors:

$$\begin{array}{c} \text{log}\;\text{LOX}\;\text{inh}.\;\%=3.34+2.69Mor\mathrm{29}m+5.65G2u-0.69MAXDP\\ N=18 \end{array}$$ (2)

**FIGURE 4:**
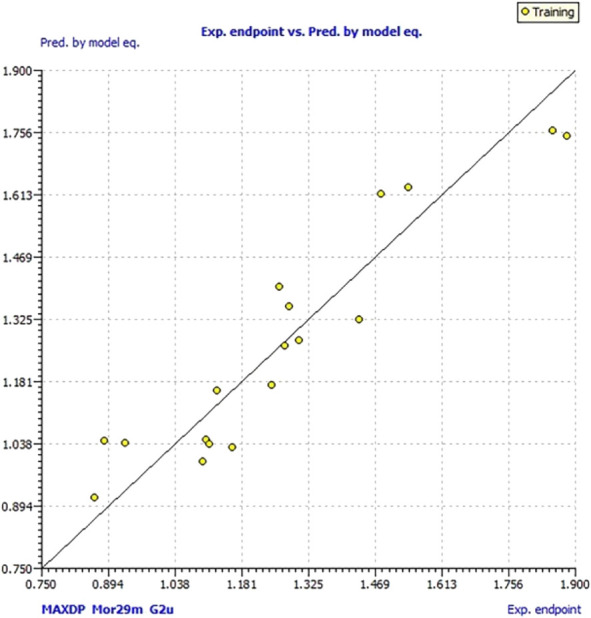
Graph of experimental vs. calculated values of log LOX inh. % by the model (2).

**FIGURE 5:**
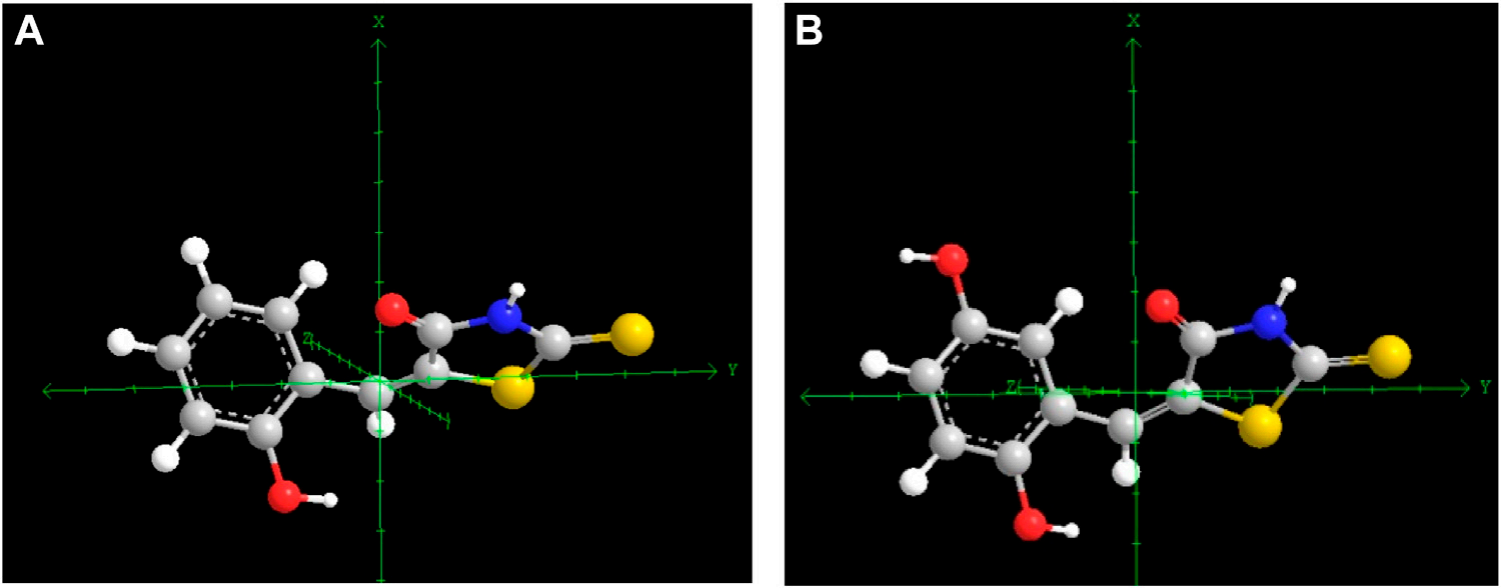
Difference in the symmetry of structures molecules **1a** **(A)** and **1c** **(B)** respect to axes y..

According to the statistical parameters presented in Table 6, a significantly improved model was obtained. It is especially improved *CCC*_cv_ on 0.88, which is greater than the threshold values (0.85), commonly used by the proposed validation criteria (Chirico and Gramatica, 2011). Graph of experimental vs. calculated values of log LOX inh. % by the model (2) is presented in Figure 4. The descriptor *Mor29m* belongs to the 3D molecular representations of the structure based on electron diffraction (3D-MoRSE) descriptors (Schuur et al., 1996). This descriptor codes pairs of atoms weighted by atomic mass at the scattering parameter 28 Å. (Devinyak et al., 2014). The relatively large scattering parameter of this descriptor conditions small discriminative power for interatomic differences between heavy atoms. The greater the number of atoms, higher the atomic mass at the short distance decreases the value of *Mor29m*, which influences lower inhibition. Thus, in the least active compound (**1g**) (log LOX inh. % = 0.86), the three methoxy groups are at the close positions, R_2_, R_3_, and R_4_, which lower *Mor29m* to a negative value (−0.9), while in **1h**, where the–OCH_3_ are in distant positions, R_1_ and R_4_, a value of *Mor29m* increased to 0.02 and inhibition effect is better (log LOX inh. % = 1.25). The most active compound, **1c,** has two hydroxyl groups in the distant positions (R_1_ and R_4_), while the second most active, compound **1s**, instead of benzylidene group substituted with hydroxyl or methoxy groups possesses the unsubstituted indole group. Both compounds have positive values of *Mor29m* (0.04 and 0.16, respectively) (Supplementary Material S2a). The second variable in models (1) and (2) is *G2u*, unweighted three-dimensional molecular index (WHIM) descriptors. This descriptor evaluates molecular symmetry on the basis of the number of symmetric atoms along a second axis of the molecule (Todeschini and Gramatica, 1997). According to the positive coefficient of *G2u* in QSAR models (1–2), higher values of this descriptor imply molecules with enhanced LOX inhibition. Therefore, molecule **1c** has one of the highest values of *G2u* (0.23) (Supplementary Material S2a). The two hydroxyl groups were symmetrically located at positions R_1_ and R_4_, which probably affected the inhibitory activity. Molecule **1a**, which possesses only one hydroxyl group at the position R_1_, is asymmetrical; therefore, it has lower *G2u* (0.18) and a weaker inhibition effect (Figure 5). The third descriptor in QSAR models (1–2) is the topological descriptor, a maximal electrotopological positive variation (*MAXDP*). Molecular electrotopological variation is the sum over all atoms of the intrinsic state differences. *MAXDP* is a measure of the total charge transfer in the molecule and it is related to the electrophilicity of the molecule (Gramatica et al., 2000). The maximal value of descriptor *MAXDP* has molecule **1g** (4.65), which is also the least active (Supplementary Material S2a). This result is consistent with the QSAR study of lipid peroxidation–inhibition potential of phenolic compounds, which revealed that *MAXDP* also has negative contributions to the activity (Ray et al., 2008). A study by Roussaki et al. (2010), Roussaki et al. (2014) has shown that the presence of the prenyloxy group in coumarin derivatives enhances lipophilicity and thus their soybean lipoxygenase inhibitory activity. Our previous QSAR study of the coumarin derivatives for lipoxygenase inhibition revealed that molecules with more atoms and higher polarizabilities in a radius of 3.5 Å from the geometrical center increase lipoxygenase inhibition (Lončarić et al., 2020).

**TABLE 5:** Correlation matrix between descriptors included in model (1).

	log LOX inh. %	*MAXDP*	*Mor29m*	*G2u*
log LOX inh. %	1.00	−0.03	0.74	0.21
*MAXDP*	−0.03	1.00	0.33	−0.15
*Mor29m*	0.74	0.33	1.00	−0.31
*G2u*	0.21	−0.15	−0.31	1.00

**TABLE 6:** The statistical parameters of the QSAR models (1) and (2).

Parameter	Model (1)	Model (2)
*N*_compounds_	19	18
*R* ^2^	0.82	0.88
*R* ^2^_adj_	0.79	0.85
*s*	0.13	0.11
*F*	23.45	33.69
*Kxx*	0.27	0.29
*ΔK*	0.05	0.10
*RMSE*_tr_	0.11	0.10
*MAE*_tr_	0.10	0.09
*CCC*_tr_	0.90	0.94
*Q* ^*2*^_LOO_	0.70	0.77
*Q* ^*2*^_LMO_	0.66	0.75
*RMSE*_cv_	0.15	0.14
*MAE*_cv_	0.13	0.12
*PRESS*_cv_	0.43	0.33
*CCC*_cv_	0.83	0.88
*R* ^*2*^_Yscr_	0.17	0.17
*Q* ^*2*^_*Yscr*_	−0.38	−0.42
*RMSE*_average_ _Yscr_	0.25	0.25
*r* ^2^_m_	0.67	0.70
Applicability domain		
*N*_compounds_ outlier (st. res. > 2.0)	1 (**1a**)	-
*N*_compounds_ out of app.dom	-	-

LOO (the leave-one out); LMO (the leave-more out); *R* ^2^ (coefficient of determination); *R* ^2^_adj_ (adjusted coefficient of determination); *s* (standard deviation of regression); *F* (Fisher ratio); *Kxx* (global correlation among descriptors); Δ*K* (global correlation among descriptors); _tr_ = training set; _cv_ = cross-validation); _Yscr_ = Y-scramble; *RMSE*, root-mean-square error; *MAE*, mean absolute error; CCC, concordance correlation coefficient; *Q* ^2^ = variance explained; PRESS, predictive error sum of squares; *r* ^2^_m_ = absolute difference between the *R* ^2^ and *R* ^2^_0_ (determination coefficients with and without intercept); h* (warning leverage for the applicability domain of the model).

### 3.4 Molecular Docking With Soybean LOX-3

**FIGURE 6:**
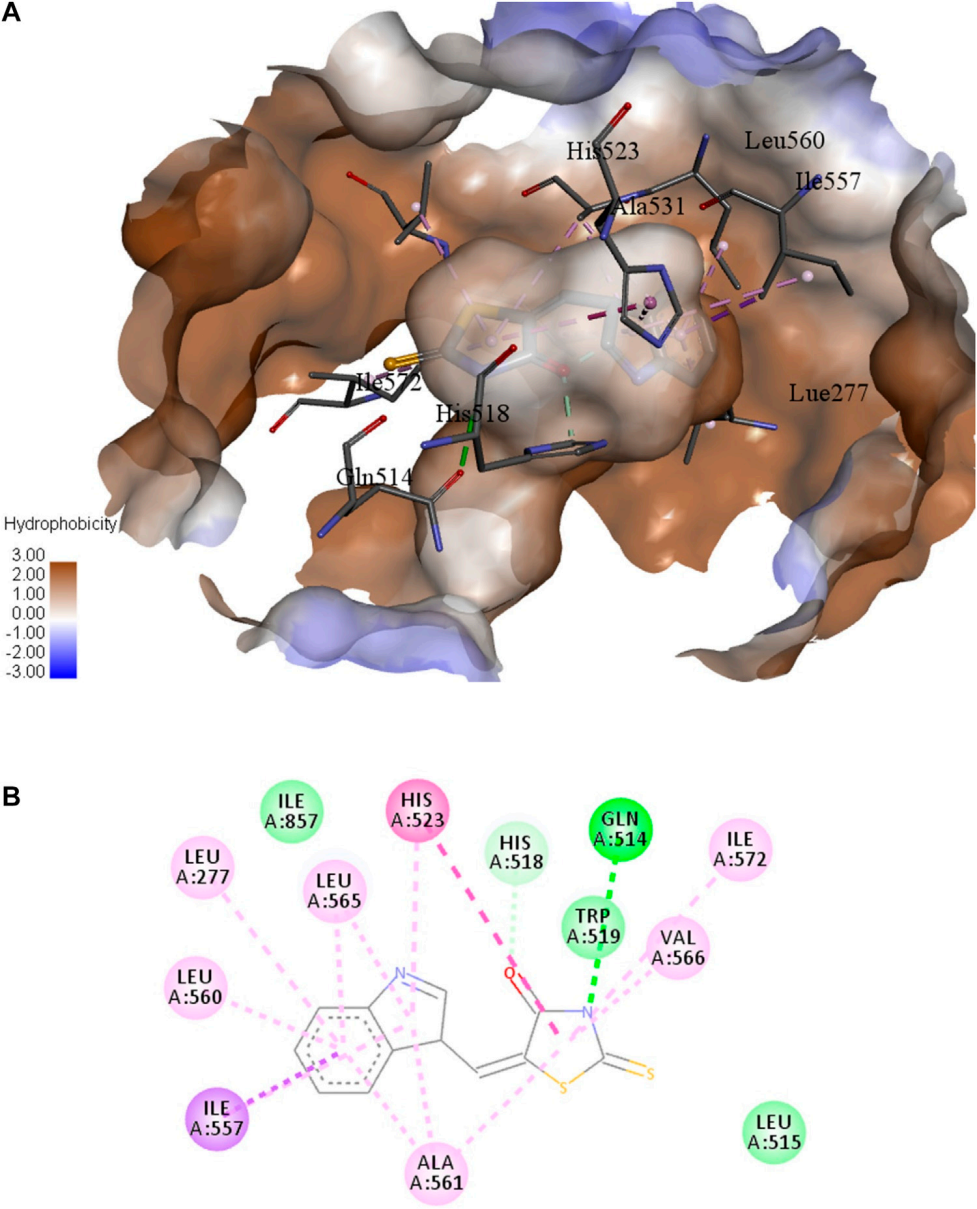
**(A)** Hydrophobic surface representation of soybean LOX-3 binding site with docked compound **1s**. **(B)** 2D representation of the main interactions of compound **1s** with residues in binding site of LOX-3: green = conventional hydrogen bond; light green = van der Waals; very light green = carbon-hydrogen bond; dark purple = π-sigma; purple = π-π T-shaped; light purple = alkyl and π-alkyl.

In order to predict the docked conformations, 19 synthesized thiazolidinediones, and extracted ligand 4NC, as inhibitors of soybean LOX-3 (PDB ID: 1NO3), molecular docking was performed. The total energy of a predicted pose (fitness) and the energy of interactions between the protein residue and ligand are tabulated in Table 7. The results of molecular docking confirmed the experimentally obtained results. The highest binding energy has compound **1s**, which exhibited the second highest inhibition against LOX-3 (Table 4). The standard ligand, 4NC, achieved only the sixth position in the score with a total energy of −64.74 kcal mol^−1^. The energies of the main interactions between compound **1s** and residues of the LOX-3 binding pocket are presented in Table 8. The docked position of the compound **1s** in the binding site of LOX-3 is presented in Figure 6A, while the 2D presentations of the main interactions with residues are shown in Figure 6B. Inhibitor **1s** is anchored in the hydrophobic channel lined by the side chains of Lue277, Ile857, Leu560, Ile557, and Ala561. Hydrogen bond generates nitrogen from thiazolidine with Gln514, while the carbon hydrogen bond is present between the oxygen atom and His518. Residue His523 generates a strong π–sigma interaction with the pyrrole ring. Indole group also generates interactions with Leu565, Lue277, Leu560, Ile557, and Ala561. X-ray structure of soybean LOX-3 complex with 4-nitrocatechol (4NC) revealed that the two hydroxyl groups interact with His523 (Skrzypczak-Jankun et al., 2004). The docking study of soybean LOX-3 with coumarin derivatives showed that those ligands formed H-bond interactions with His513, Gln514, His518, Trp519, and Asp766. The formation of H-bond between the oxygen atom from the benzoyl group (Lončarić et al., 2020) was important.

**TABLE 7:** Total energies of a predicted poses (fitness) and energies of interactions (hydrogen-bonding (*H*), van der Waals (*V*), and electrostatic (*E*)) between protein residue and ligand (kcal mol^−1^) in the binding site of soybean LOX-3 (1NO3).

Compound	Pose	Fitness	*H*	*V*	*E*
**1s**	2	−72.91	−18.42	−54.45	0.00
**1g**	2	−72.87	−1.60	−71.31	0.00
**1o**	0	−70.08	−9.48	−60.61	0.00
**1c**	2	−67.68	−11.74	−55.93	0.00
**1e**	2	−66.46	−2.73	−63.73	0.00
**4NC**	2	−64.74	−15.06	−51.49	1.81
**1b**	0	−64.62	−7.84	−56.78	0.00
**1j**	1	−63.72	−20.09	−44.82	1.19
**1a**	2	−63.65	−15.53	−48.12	0.00
**1q**	0	−62.70	−7.30	−55.40	0.00
**1n**	2	−62.47	−3.50	−58.97	0.00
**1h**	1	−61.41	−8.27	−53.13	0.00
**1f**	0	−60.81	−5.69	−55.12	0.00
**1d**	2	−60.66	−3.36	−57.30	0.00
**1k**	2	−60.13	0.00	−60.13	0.00
**1m**	2	−58.51	−9.36	−49.16	0.00
**1l**	2	−57.36	−2.50	−54.86	0.00
**1p**	1	−56.65	−3.17	−53.48	0.00
**1r**	2	−55.38	−10.50	−44.88	0.00
**1i**	1	−54.50	−7.00	−47.50	0.00

**TABLE 8:** Energy of the main interactions between LOX-3 residues and ligand **1s** (M = main chain; S = side chain).

H bond	Energy	van der Waals interaction	Energy
M-Gln514	−1.16	S-Gln514	−4.81
S-Gln514	−3.50	S-His518	−1.14
S-His518	−9.50	S-Trp519	−9.33
S-His523	−1.99	S-His523	−10.14
M-Ile857	−2.26	M-Asn558	−1.38
		S-Asn558	−0.47
		S-Leu565	−2.92
		S-Ile572	−1.77

## 4 Conclusion

Thiazolidinedione derivatives were successfully synthesized in choline chloride-based deep eutectic solvents with yields ranging from 21.5% to 90.9%. After screening 20 different DESs, the most suitable solvent for the synthesis of mentioned compounds was proven to be ChCl: *N*-methylurea. The highest inhibition of the lipoxygenase activity of 76.3% was achieved with compound **1c** (IC_50_ = 3.52 µM). Compound **1d** showed the highest inhibition of lipid peroxidation of 84.2%. Thiazolidinedione derivatives showed better inhibition of the ABTS radical than the inhibition of the DPPH radical. The highest inhibition of the ABTS radical of 100% was achieved with compounds **1f** and **1o**. QSAR study revealed the essential structural features of thiazolidine-2,4-dione derivatives for the enhanced inhibition of the soybean LOX-3, such as larger distance between atoms, higher atomic mass, symmetrical distribution of the atomic group in the molecule, and lower electrophilicity of the molecule. Molecular docking confirmed the experimentally obtained results of LOX-3 inhibition and elucidated the mode of the interactions with residues in the hydrophobic binding pocket of LOX-3. Future synthesis of thiazolidinediones as promising LOX-3 inhibitors should be limited to the lipophilic derivatives without hydroxyl and methoxy groups, or their symmetric distribution in order to prevent steric hindrance. Since thiazolidinediones are also known as insulin-sensitizing agents used in the treatment of type 2 diabetes as effective agents for attenuating insulin resistance, compounds can be tested for their anti-inflammatory and antidiabetic activities.

## Data Availability

The original contributions presented in the study are included in the article/Supplementary Material; further inquiries can be directed to the corresponding author.
