# Supplementary material for: Green Synthesis of Thiazolidine-2,4-dione Derivatives and Their Lipoxygenase Inhibition Activity With QSAR and Molecular Docking Studies
Source: Front Chem. 2022 Jul 5;10:912822. doi: 10.3389/fchem.2022.912822 (PMC9294463; doi:10.3389/fchem.2022.912822)
Supplement: Supplementary file 1 [file DataSheet2.docx]

**Green Synthesis of Thiazolidine-2,4-dione Derivatives and their Lipoxygenase Inhibition Activity**

**Melita Lončarić, Ivica Strelec, Vesna Rastija, Maja Karnaš, Maja Molnar**

**Supplementary material 2a:** The values of molecular descriptors included in model (1).

| **Compound** | **log LOX inh. %** | ***Mor29m*** | ***G2u*** | ***MAXDP*** |
| --- | --- | --- | --- | --- |
| **1a** | 1.14 | 0.03 | 0.18 | 4.33 |
| **1b** | 0.93 | -0.13 | 0.20 | 4.44 |
| **1c** | 1.88 | 0.04 | 0.23 | 4.35 |
| **1d** | 1.43 | -0.08 | 0.22 | 4.43 |
| **1e** | 1.16 | -0.14 | 0.20 | 4.42 |
| **1f** | 1.13 | -0.13 | 0.21 | 4.34 |
| **1g** | 0.86 | -0.09 | 0.18 | 4.65 |
| **1h** | 1.25 | 0.02 | 0.17 | 4.58 |
| **1i** | 0.89 | -0.24 | 0.24 | 4.38 |
| **1j** | 1.26 | 0.06 | 0.18 | 4.44 |
| **1k** | 1.48 | 0.04 | 0.22 | 4.46 |
| **1l** | 1.31 | -0.04 | 0.18 | 4.32 |
| **1m** | 1.10 | -0.12 | 0.18 | 4.32 |
| **1n** | 1.10 | -0.12 | 0.19 | 4.47 |
| **1o** | 1.54 | 0.16 | 0.19 | 4.63 |
| **1p** | 1.11 | -0.06 | 0.22 | 4.85 |
| **1q** | 1.28 | 0.06 | 0.18 | 4.56 |
| **1r** | 1.27 | -0.11 | 0.22 | 4.30 |
| **1s** | 1.85 | 0.16 | 0.20 | 4.55 |

**Supplementary material 2b:** The values of experimentally determined logarithmic values of % inhibition of soybean lipoxygenase, calculated by model (1), and residuals.

| **Compound** | **log LOX inh. % exp.** | **log LOX inh. % calc.** | **Residuals** |
| --- | --- | --- | --- |
| **1a** | 1.14 | 1.39 | 0.25 |
| **1b** | 0.93 | 1.03 | 0.10 |
| **1c** | 1.88 | 1.73 | -0.16 |
| **1d** | 1.43 | 1.32 | -0.11 |
| **1e** | 1.16 | 1.02 | -0.14 |
| **1f** | 1.13 | 1.14 | 0.01 |
| **1g** | 0.86 | 0.92 | 0.06 |
| **1h** | 1.25 | 1.15 | -0.09 |
| **1i** | 0.89 | 1.07 | 0.18 |
| **1j** | 1.26 | 1.35 | 0.09 |
| **1k** | 1.48 | 1.60 | 0.12 |
| **1l** | 1.31 | 1.23 | -0.08 |
| **1m** | 1.10 | 1.01 | -0.09 |
| **1n** | 1.10 | 0.99 | -0.11 |
| **1o** | 1.54 | 1.61 | 0.07 |
| **1p** | 1.11 | 1.10 | -0.02 |
| **1q** | 1.28 | 1.33 | 0.05 |
| **1r** | 1.27 | 1.24 | -0.03 |
| **1s** | 1.85 | 1.73 | -0.12 |
