# Supplementary material for: Green Synthesis of Thiazolidine-2,4-dione Derivatives and Their Lipoxygenase Inhibition Activity With QSAR and Molecular Docking Studies
Source: Front Chem. 2022 Jul 5;10:912822. doi: 10.3389/fchem.2022.912822 (PMC9294463; doi:10.3389/fchem.2022.912822)

**Green Synthesis of Thiazolidine-2,4-dione Derivatives and their Lipoxygenase Inhibition Activity with QSAR and Molecular Docking Studies**

Melita Lončarić^1^, Ivica Strelec^1^, Valentina Pavić^2^, Vesna Rastija^3^, Maja Karnaš^3^, Maja Molnar^1*^

**Supplementary material 1:** Spectral data for synthesized thiazolidinedione derivatives (Mass spectra, ^1^H and ^13^C spectra)

**5-(2-hydroxybenzylidene)thiazolidine-2,4-dione (1a)**

Using thiazolidinedione (0.234 g, 2 mmol) and salicylaldehyde (215 µL, 2 mmol), in accordance with the General Procedure, the title compound **1a** was obtained (0.159 g, 35.9 % yield) as a yellow solid (m.p. 269 – 272 °C). **^1^H** (600 MHz) δ 12.48 (s, 1H, NH), 10.48 (s, 1H, OH), 8.00 (s, 1H, CH), 7.30 (q, *J* = 7.92; 9.84; 7.26 Hz, 2H, arom.), 6.93 (q, *J* = 8.40; 9.48; 7.50 Hz, 2H, arom.). **^13^C** (150 MHz) δ 168.15; 167.50; 157.24; 132.20; 128.20; 126.27; 121.87; 119.64; 116.08.

**Mass spectrum (1a)**


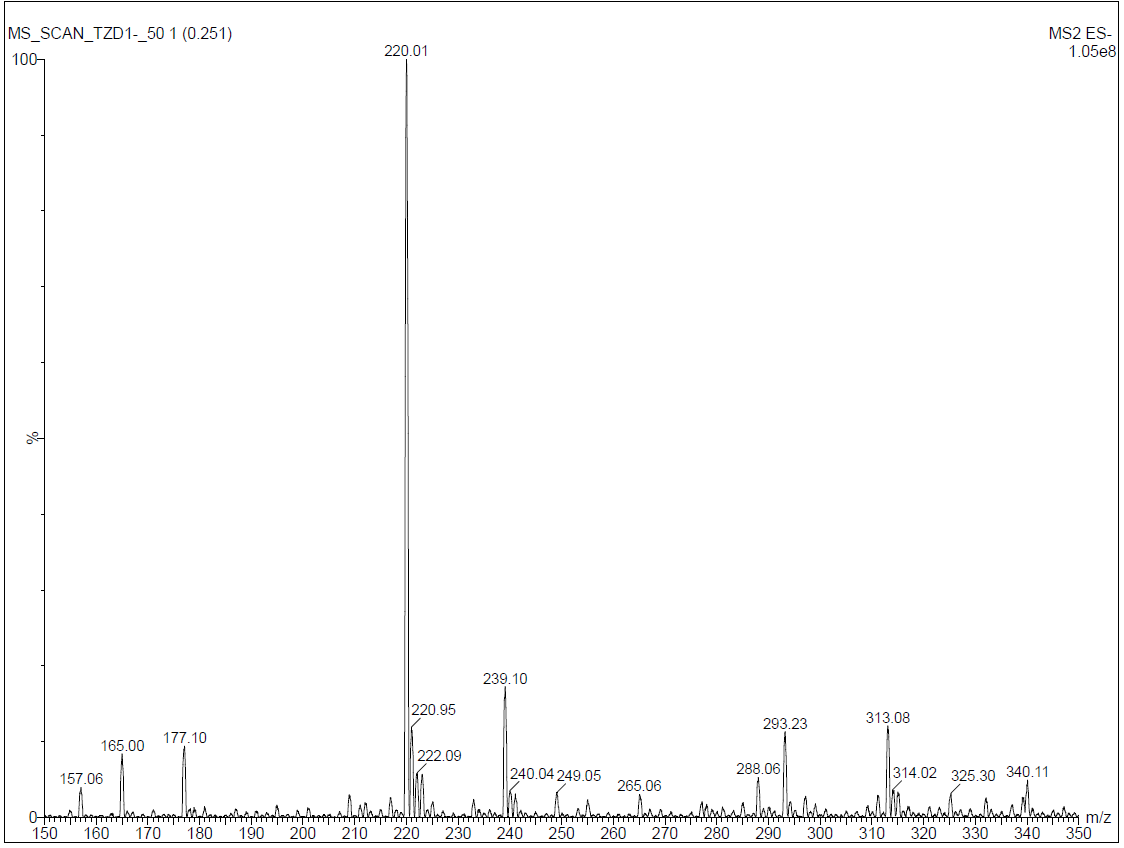


**^1^H NMR spectrum (1a)**


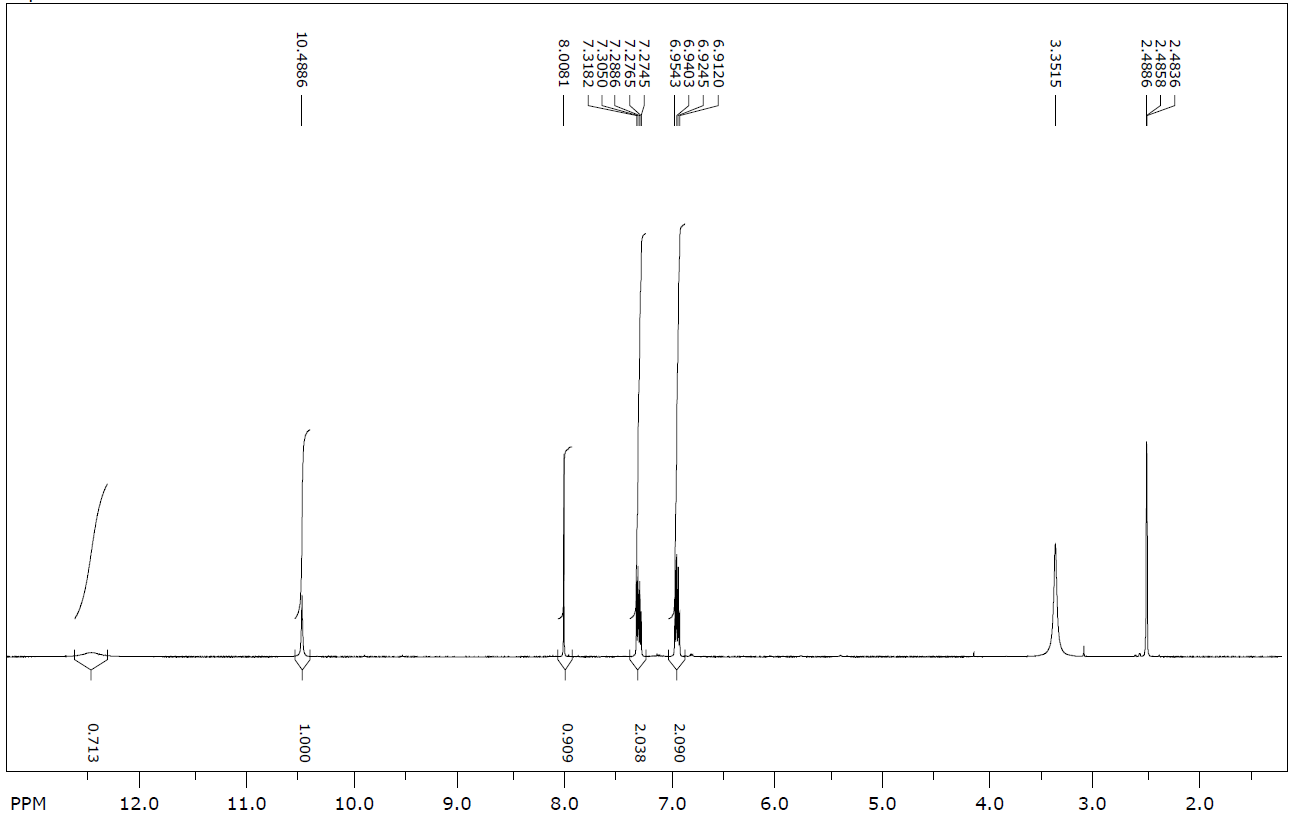


**^13^C NMR spectrum (1a)**


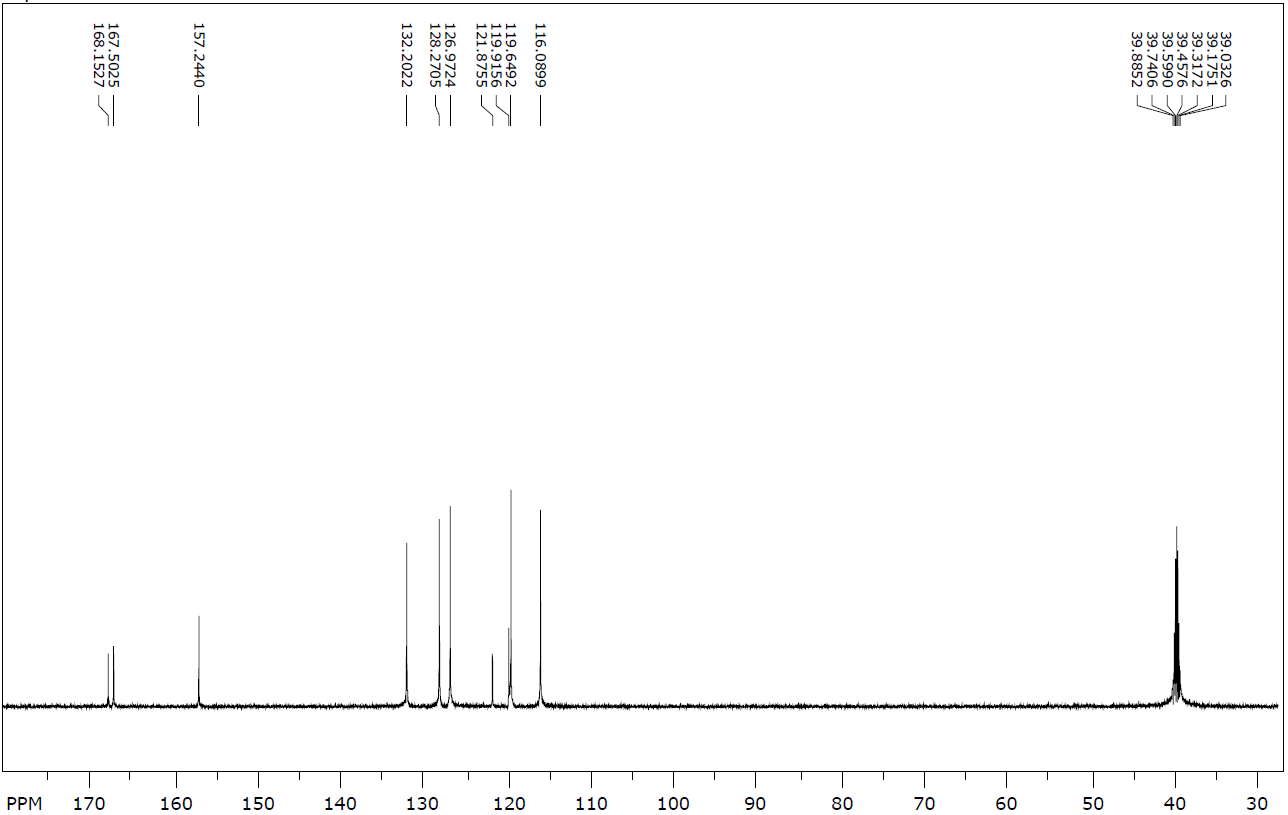


**5-(4-hydroxy-3-methoxybenzylidene)thiazolidine-2,4-dione (1b)**

Using thiazolidinedione (0.234 g, 2 mmol) and 4-hydroxy-3-methoxybenzaldehyde (0.304 g, 2 mmol), in accordance with the General Procedure, the title compound **1b** was obtained (0.343 g, 68.3 % yield) as a brown solid (m.p. 199 – 201 °C). **^1^H** (300 MHz) δ 11.98 (s, 1H, NH), 9.94 (s, 1H, OH), 7.71 (s, 1H, CH), 7.17 (d, *J* = 1.89 Hz, 1H, arom.), 7.07 (dd, *J* = 8.34; 1.89 Hz, 1H, arom.), 6.93 (d, *J* = 8.25 Hz, 1H, arom.), 3.82 (s, 3H, OCH_3_). **^13^C** (150 MHz) δ 172.47; 168.52; 167.94; 149.89; 148.43. 130.06; 124.85; 124.59; 119.68; 116.71; 114.60.

**Mass spectrum (1b)**


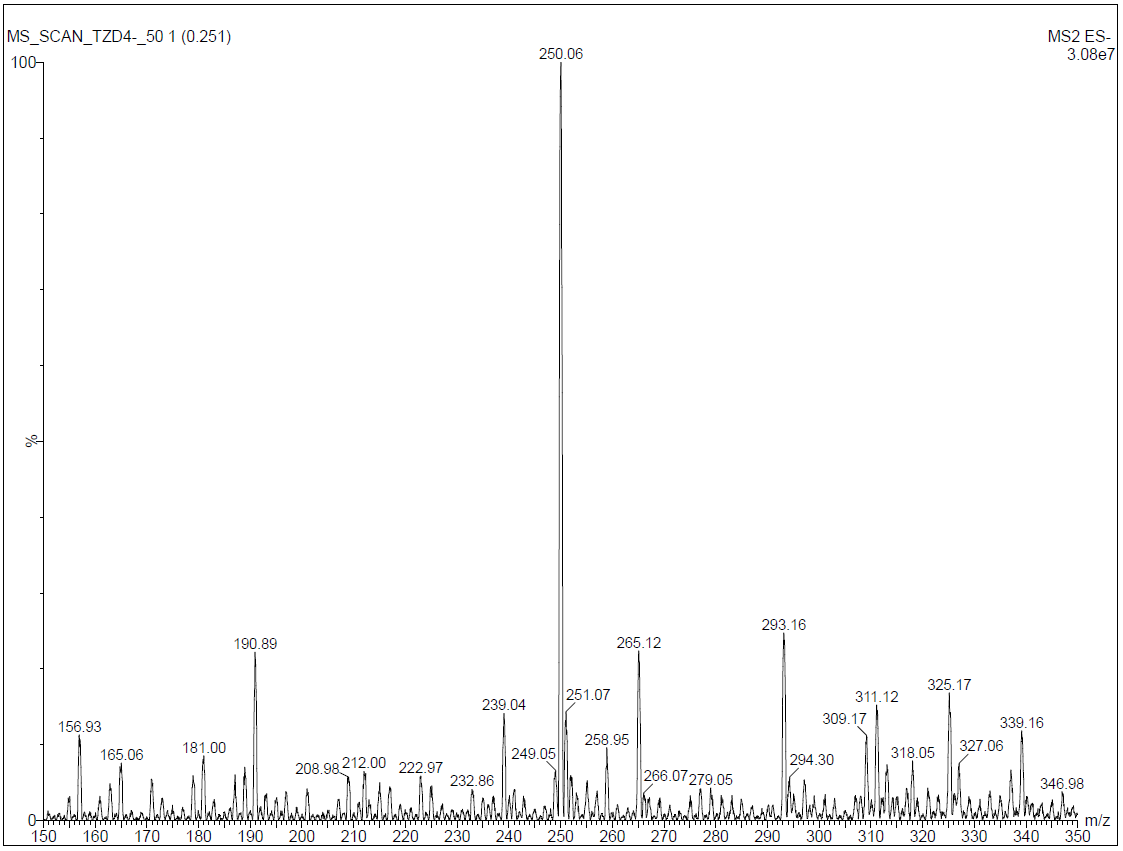


**^1^H NMR spectrum (1b)**


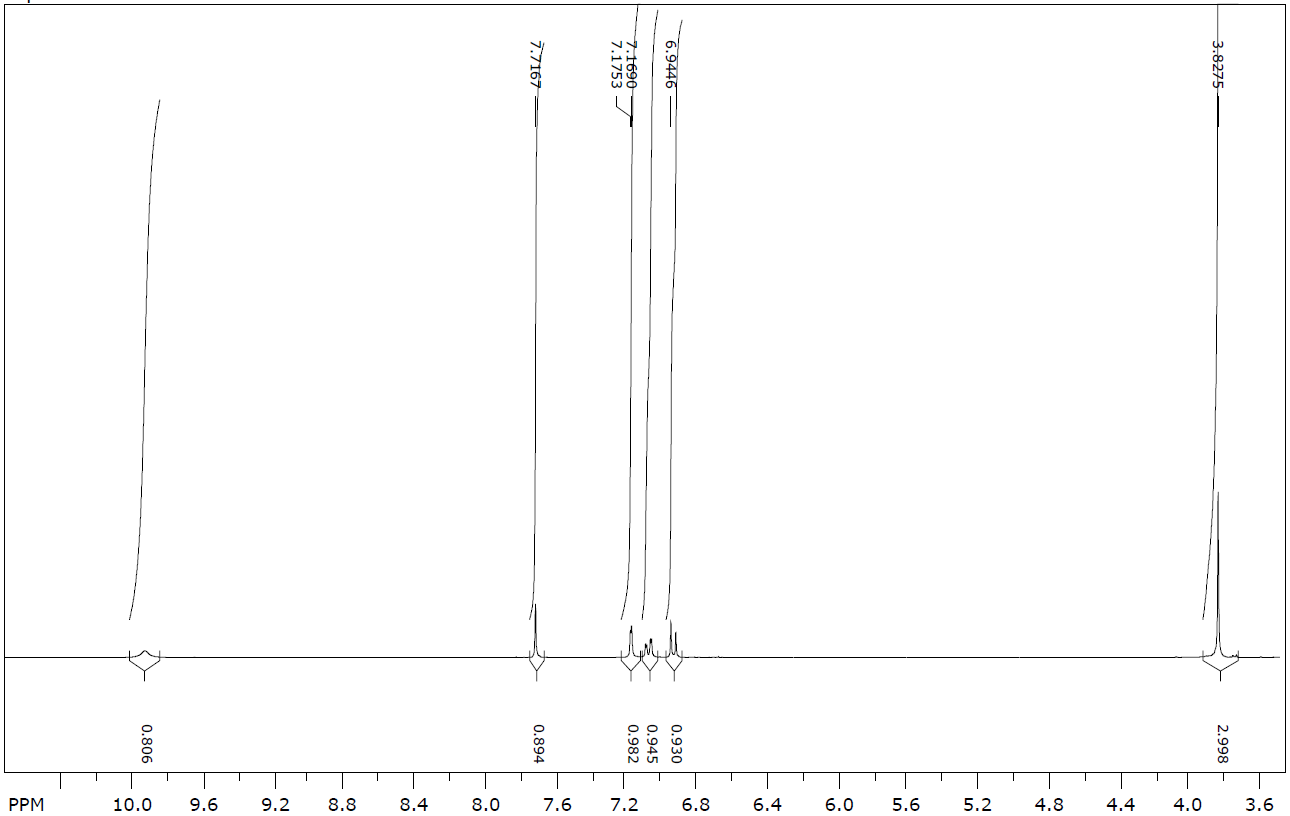


**^13^C NMR spectrum (1b)**


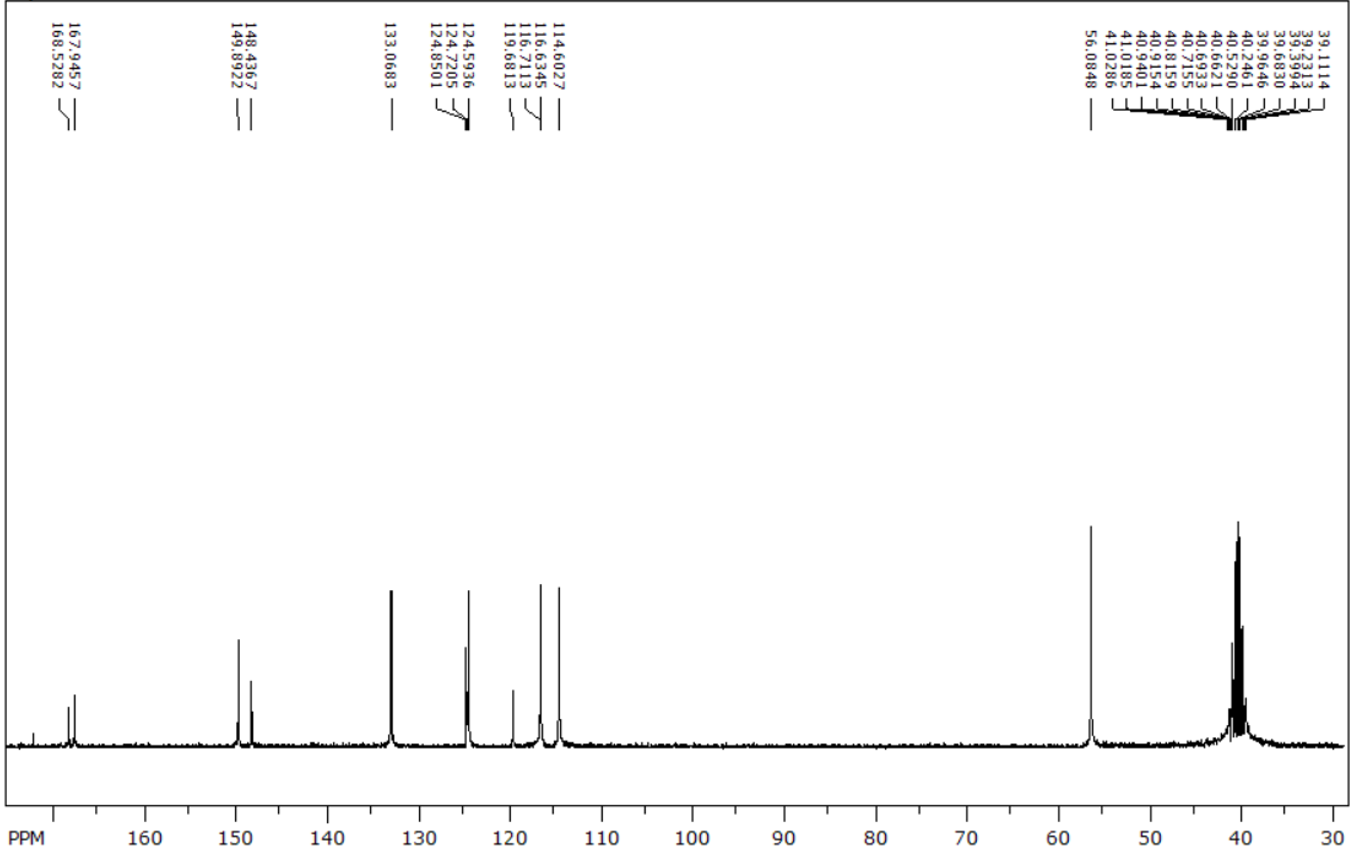


**5-(2,5-dihydroxybenzylidene)thiazolidine-2,4-dione (1c)**

Using thiazolidinedione (0.234 g, 2 mmol) and 2,5-dihydroxybenzaldehyde (0.276 g, 2 mmol), in accordance with the General Procedure, the title compound **1c** was obtained (0.102 g, 21.5 % yield) as a brown solid (m.p. 194 – 198 °C). **^1^H** (600 MHz) δ 9.70 (s, 1H, OH), 7.61 (s, 1H, CH), 7.23 (d, *J* = 8.76 Hz, 1H, arom.), 6.98 (s, 1H, arom.), 6.92 (dd, *J* = 8.22; 1.68; 2.40 Hz, 1H, arom.). **^13^C** (150 MHz) δ 153.98; 144.08; 132.23; 128.61; 120.04; 117.85; 116.75; 110.91.

**Mass spectrum (1c)**


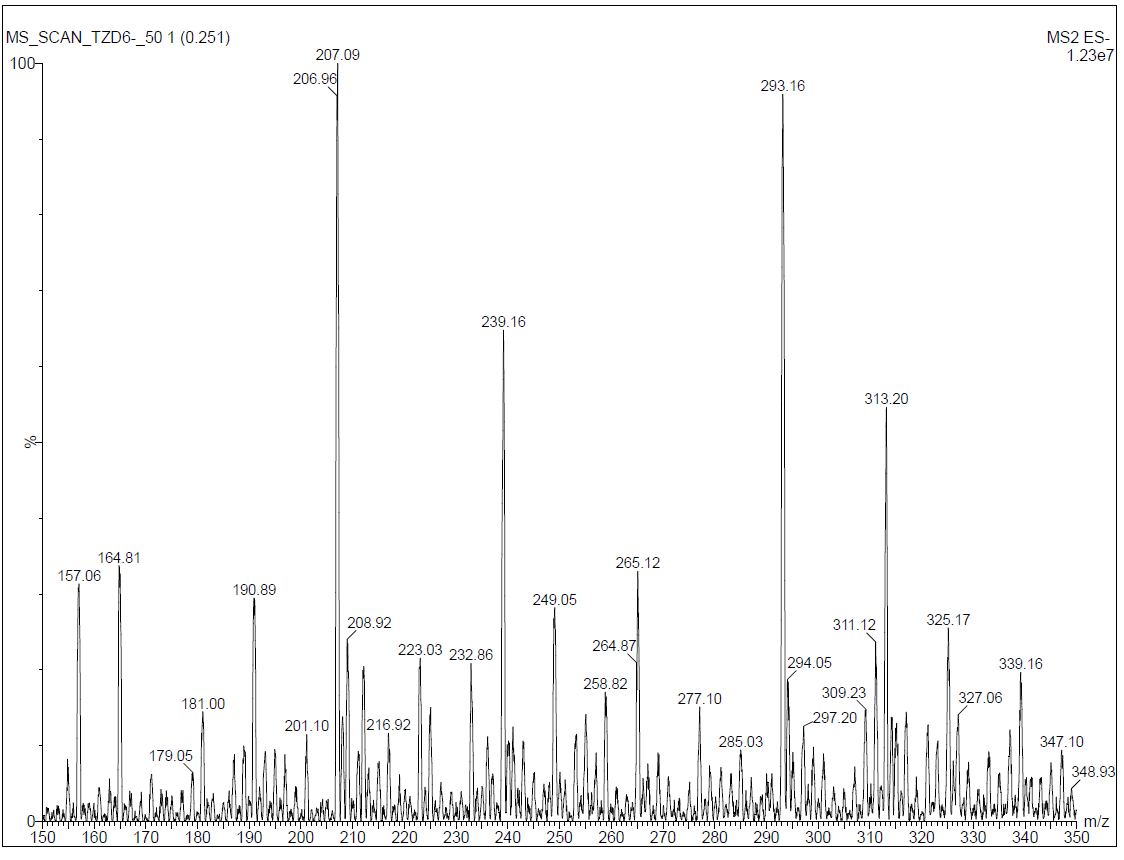


**^1^H NMR spectrum (1c)**


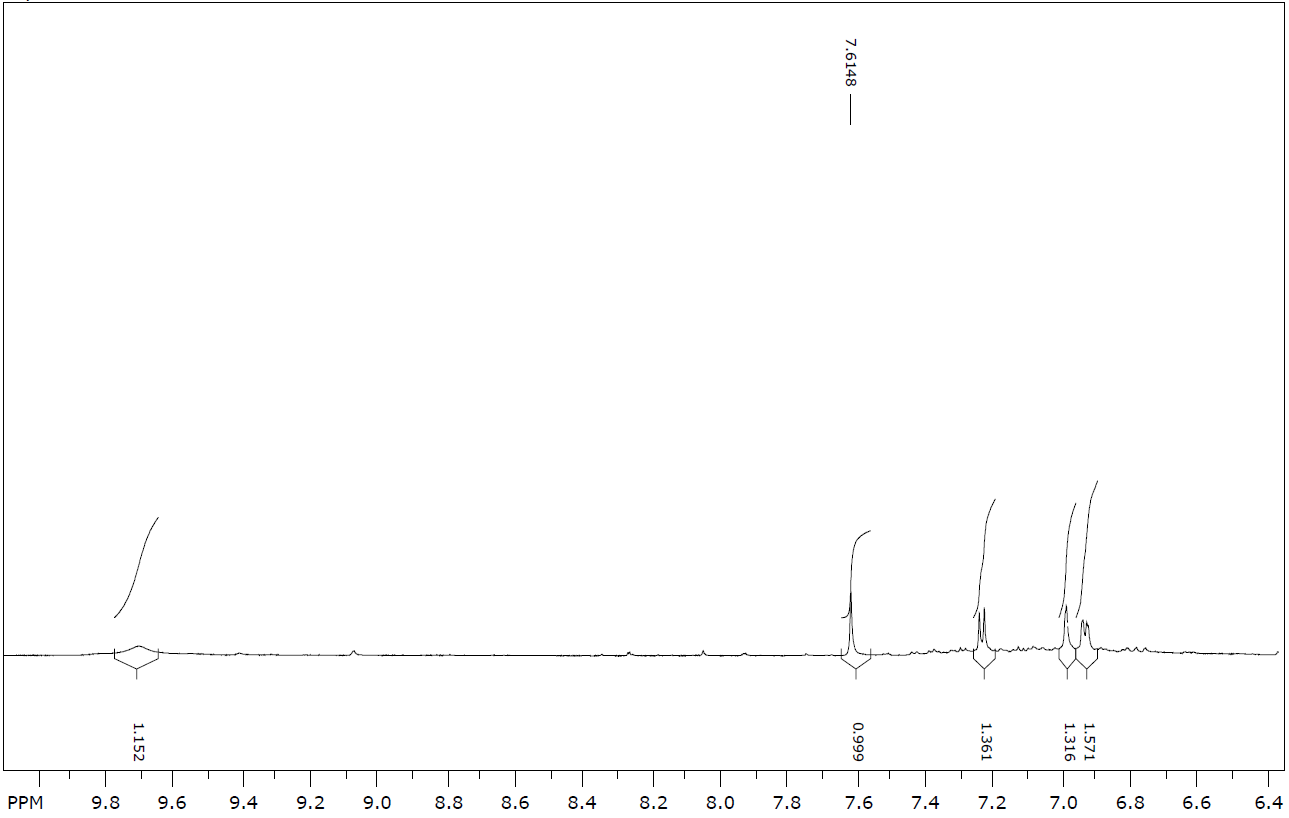


**^13^C NMR spectrum (1c)**


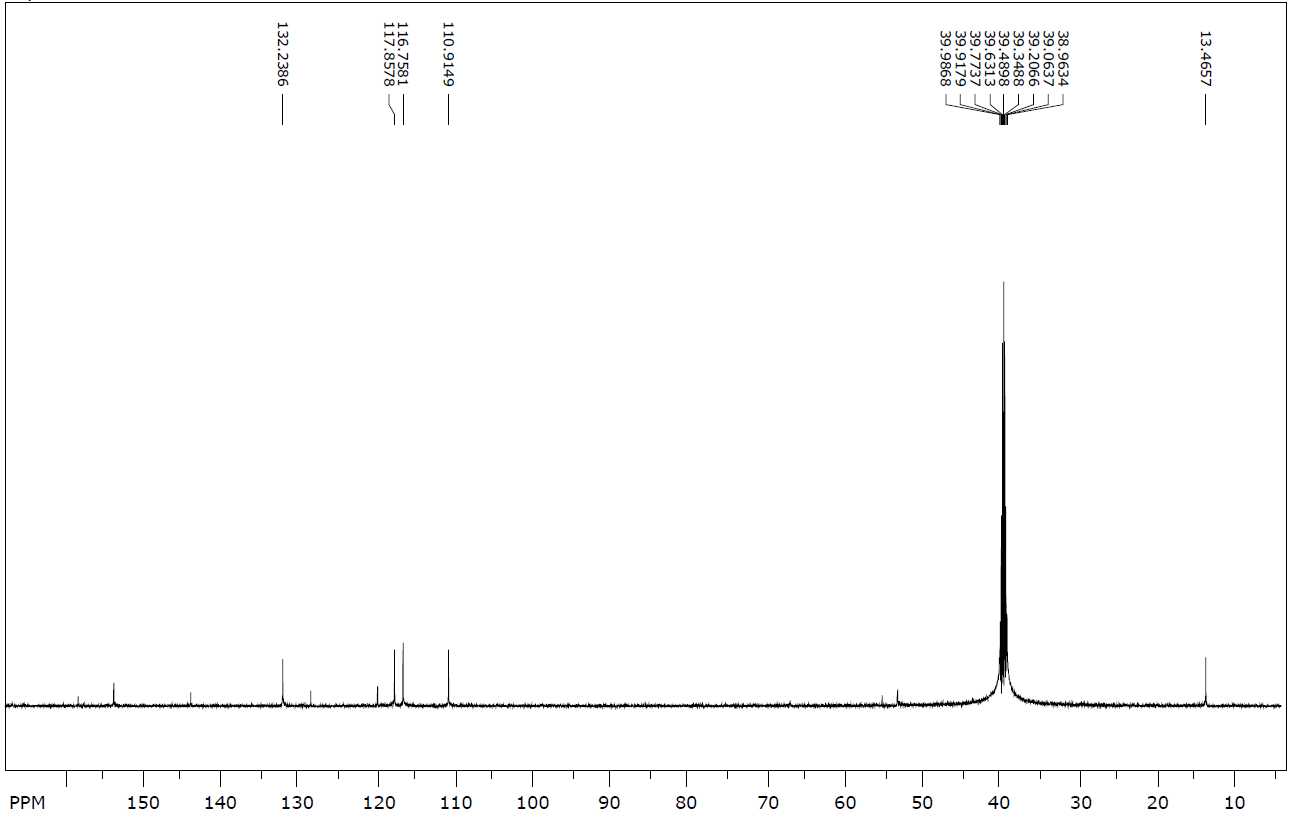


**5-(3-methoxybenzylidene)thiazolidine-2,4-dione (1d)**

Using thiazolidinedione (0.234 g, 2 mmol) and 3-methoxybenzaldehyde (245 µl, 2 mmol), in accordance with the General Procedure, the title compound **1d** was obtained (0.178 g,  37.8 % yield) as a white solid (m.p.  194 – 197 °C). **^1^H** (300 MHz) δ 12.62 (s, 1H, NH), 7.76 (s, 1H, CH), 7.45 (t, *J* = 8.16 Hz, 1H, arom.), 7.15 (d, *J* = 6.45 Hz, 2H, arom.), 7.06 (dd, *J* = 8.34; 1.89; 0.30 Hz, 1H, arom.), 3.80 (s, 3H, OCH_3_). **^13^C** (150 MHz) δ 168.28; 167.70; 160.08; 134.85; 132.21; 130.86; 122.36; 116.74; 115.76; 55.73.

**Mass spectrum (1d)**


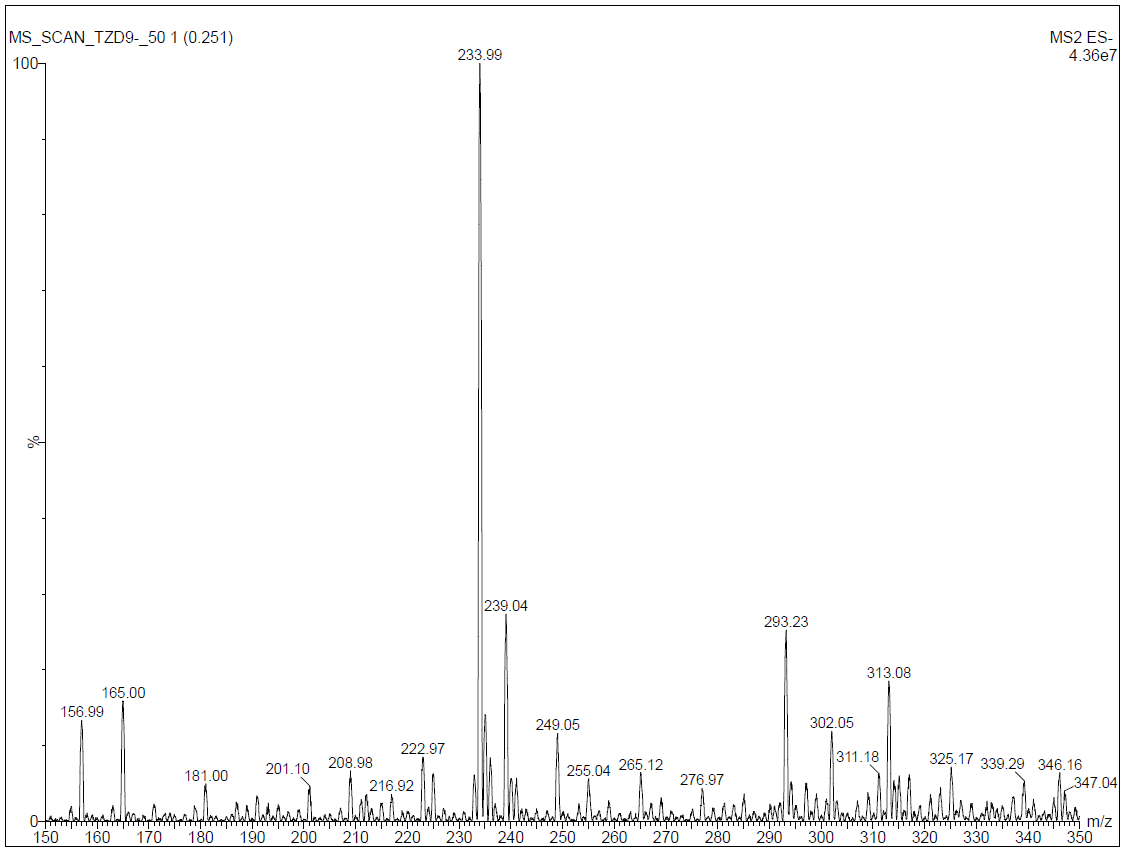


**^1^H NMR spectrum (1d)**


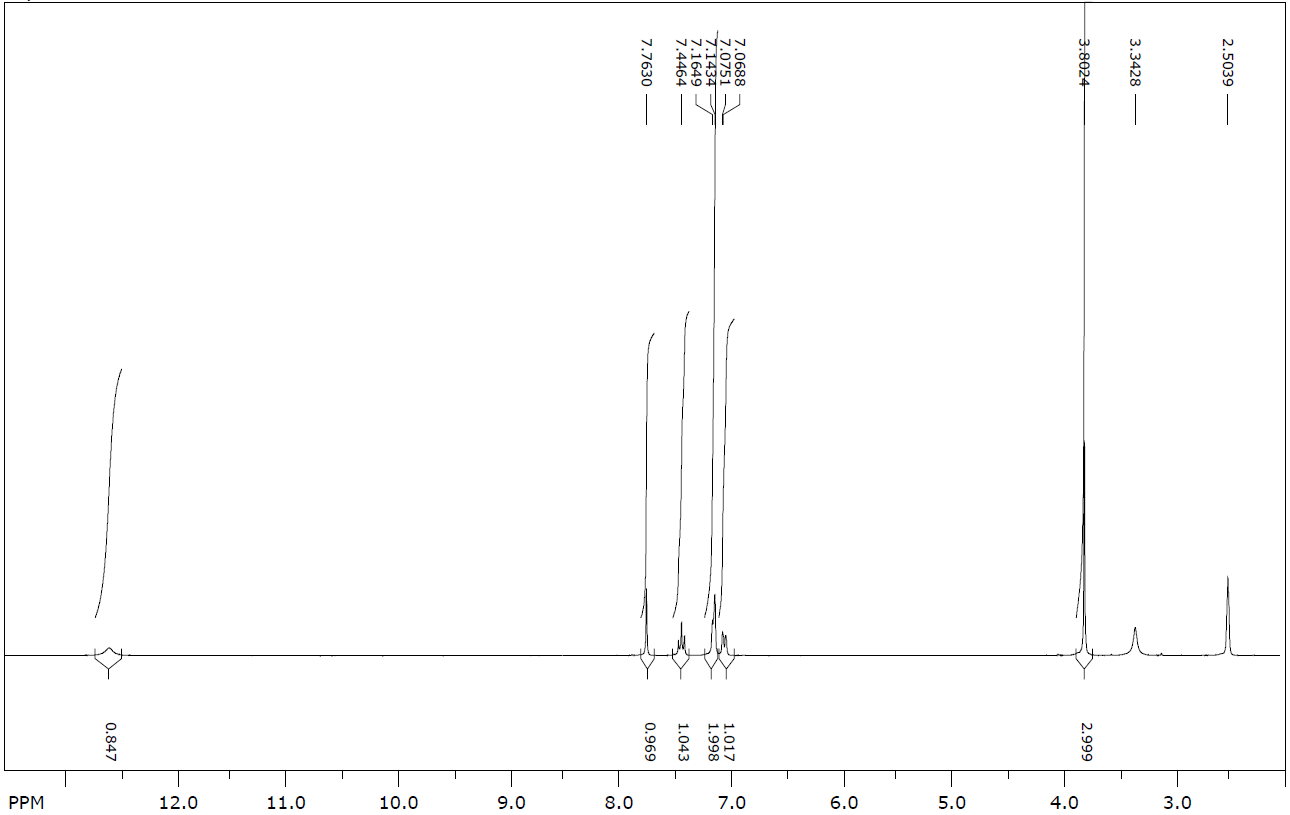


**^13^C NMR spectrum (1d)**


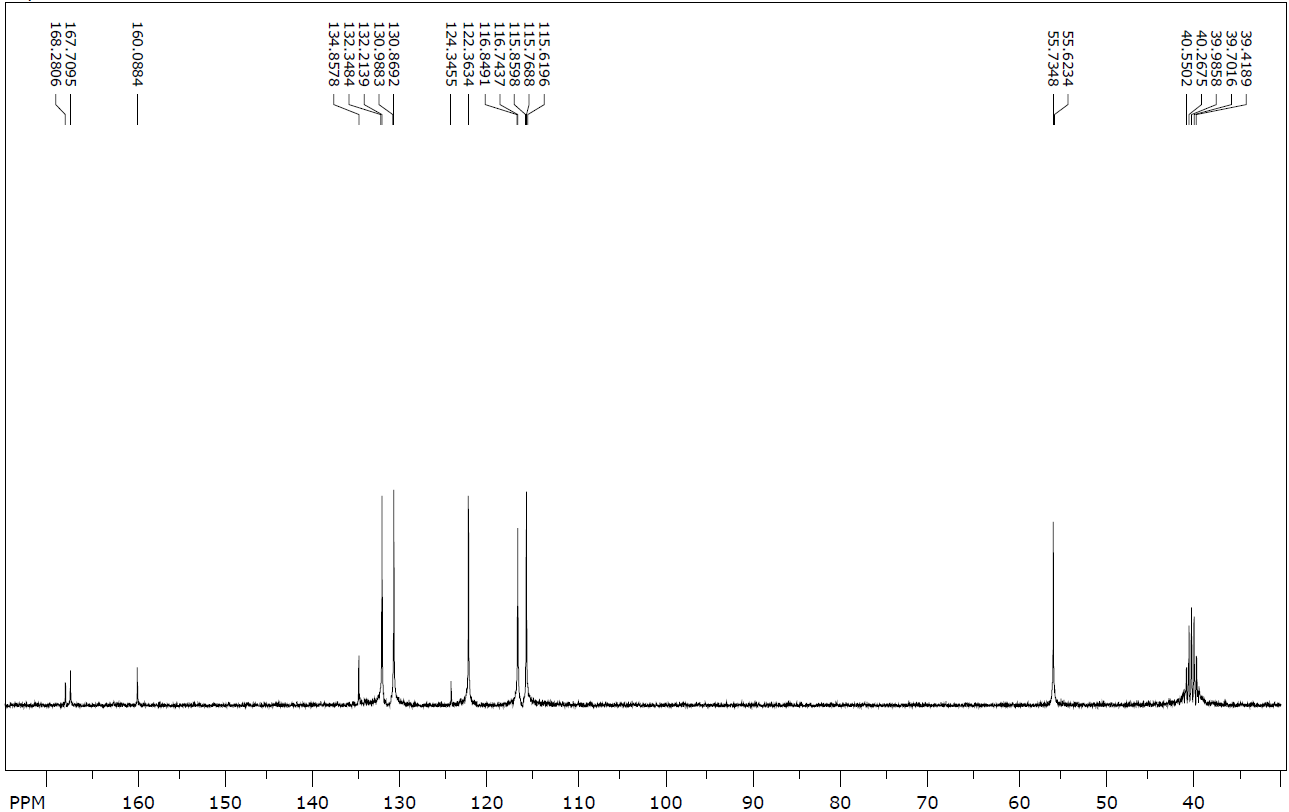


**5-(3-hydroxy-4-methoxybenzylidene)thiazolidine-2,4-dione (1e)**

Using thiazolidinedione (0.234 g, 2 mmol) and 3-hydroxy-4-methoxybenzaldehyde (0.304 g, 2 mmol), in accordance with the General Procedure, the title compound **1e** was obtained (0.271 g,  53.9 % yield) as a yellow solid (m.p. 254 – 257 °C). **^1^H** (600 MHz) δ 12.46 (s, 1H, NH), 9.47 (s, 1H, OH), 7.62 (s, 1H, CH), 7.06 (d, *J* = 2.16 Hz, 2H, arom.), 7.00 (d, *J* = 1.56 Hz, 1H, arom.), 3.81 (s, 3H, OCH_3_). **^13^C** (150 MHz) δ 168.03; 167.40; 149.99; 146.89; 132.24; 125.63; 123.45; 119.96; 115.84; 112.37; 55.63.

**Mass spectrum (1e)**


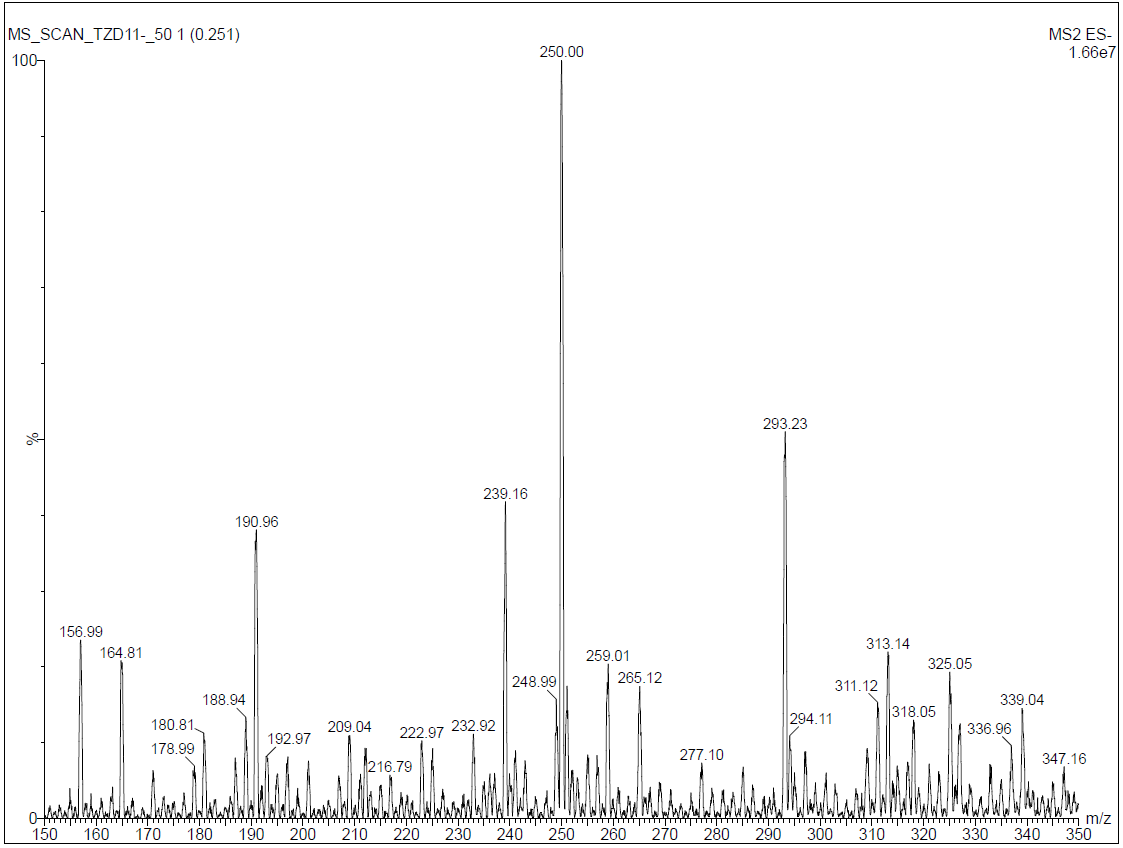


**^1^H NMR spectrum (1e)**


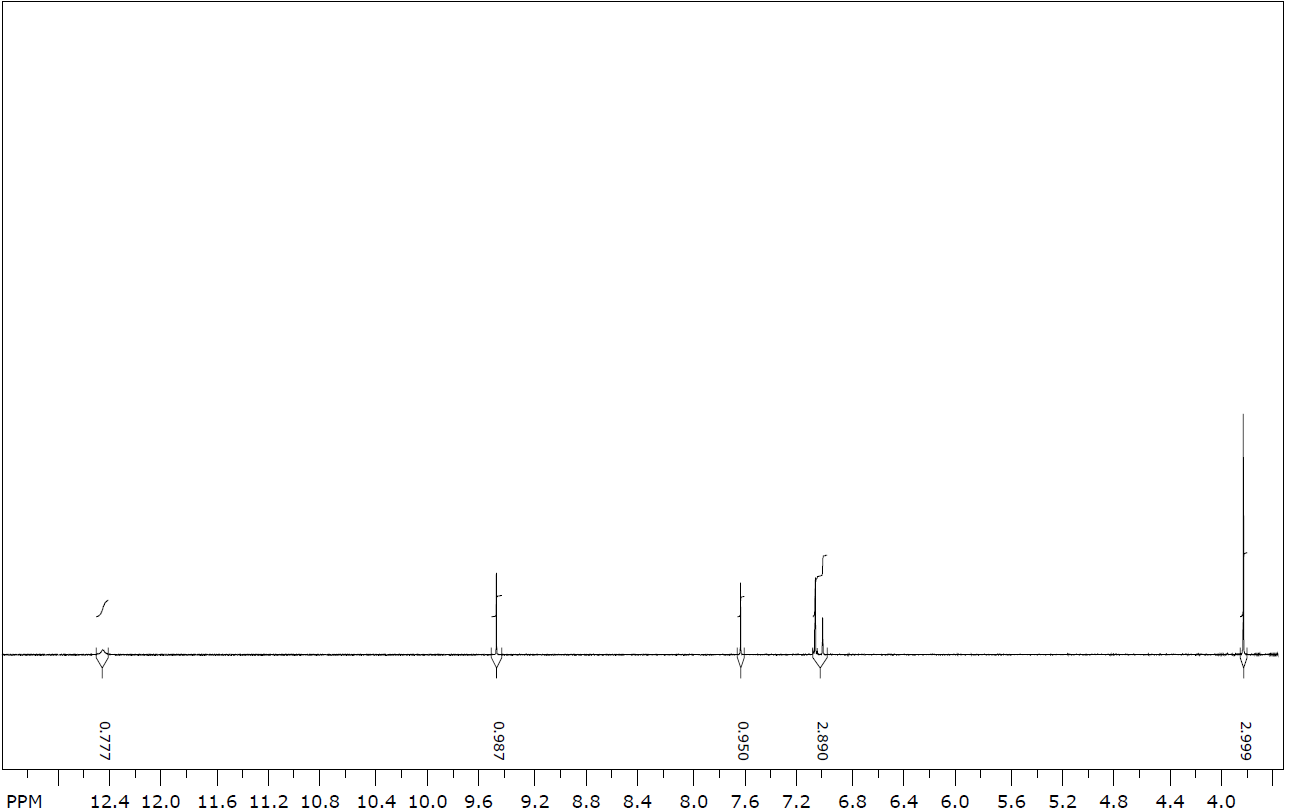


**^13^C NMR spectrum (1e)**


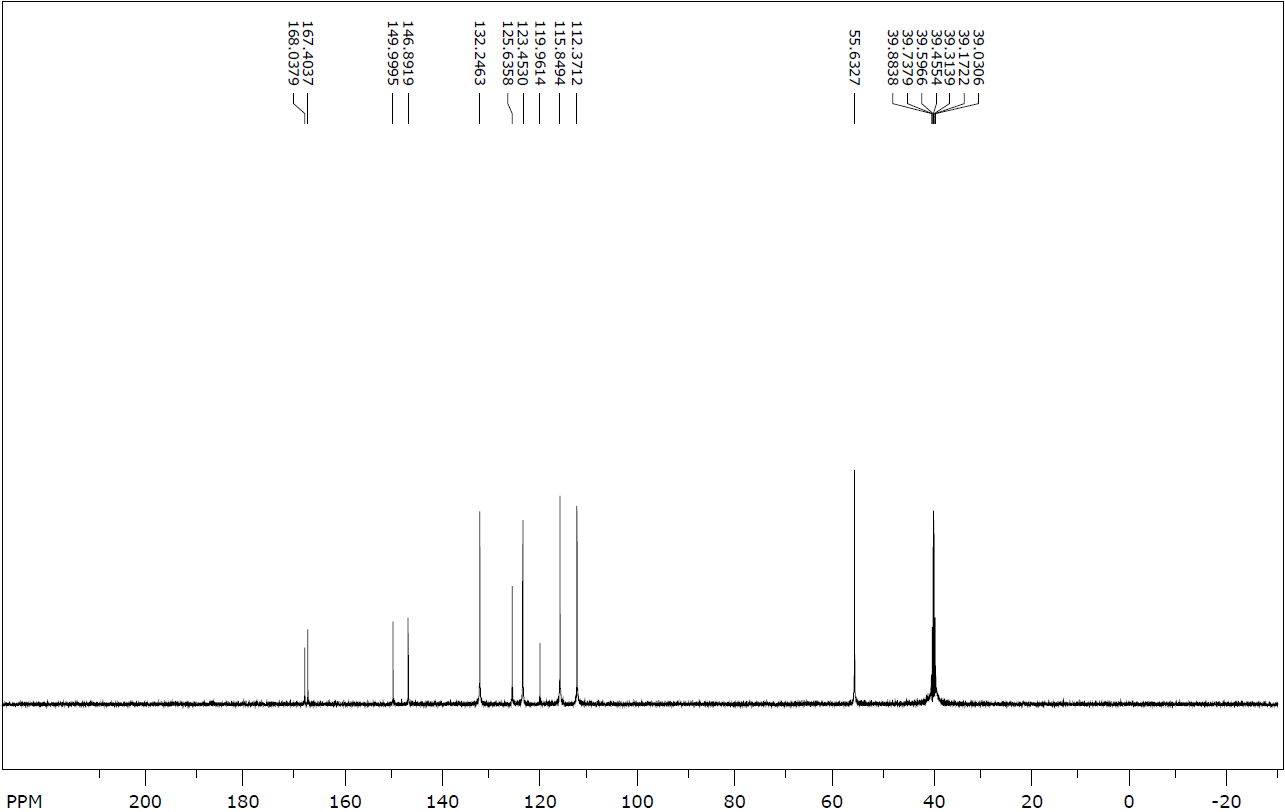


**5-(3,4-dihydroxybenzylidene)thiazolidine-2,4-dione (1f)**

Using thiazolidinedione (0.234 g, 2 mmol) and 3,4-dihydroxybenzaldehyde (0.276 g, 2 mmol), in accordance with the General Procedure, the title compound **1f** was obtained (0.139 g, 29.3 % yield) as a brown solid (m.p. 270 – 271 °C). **^1^H** (600 MHz) δ 12.42 (s, 1H, NH), 9.82 (s, 1H, OH), 9.44 (s, 1H, OH), 7.60 (s, 1H, CH), 6.99 (d, *J* = 1.98 Hz, 1H, arom.), 6.96 (q, *J* = 8.28; 1.95 Hz, 1H, arom.), 6.87 (d, *J* = 8.22 Hz, 1H, arom.). **^13^C** (150 MHz) δ 168.15; 166.53; 148.57. 145.82; 132.63; 124.28; 123.89; 118.72; 116.37; 116.22.

**Mass spectrum (1f)**


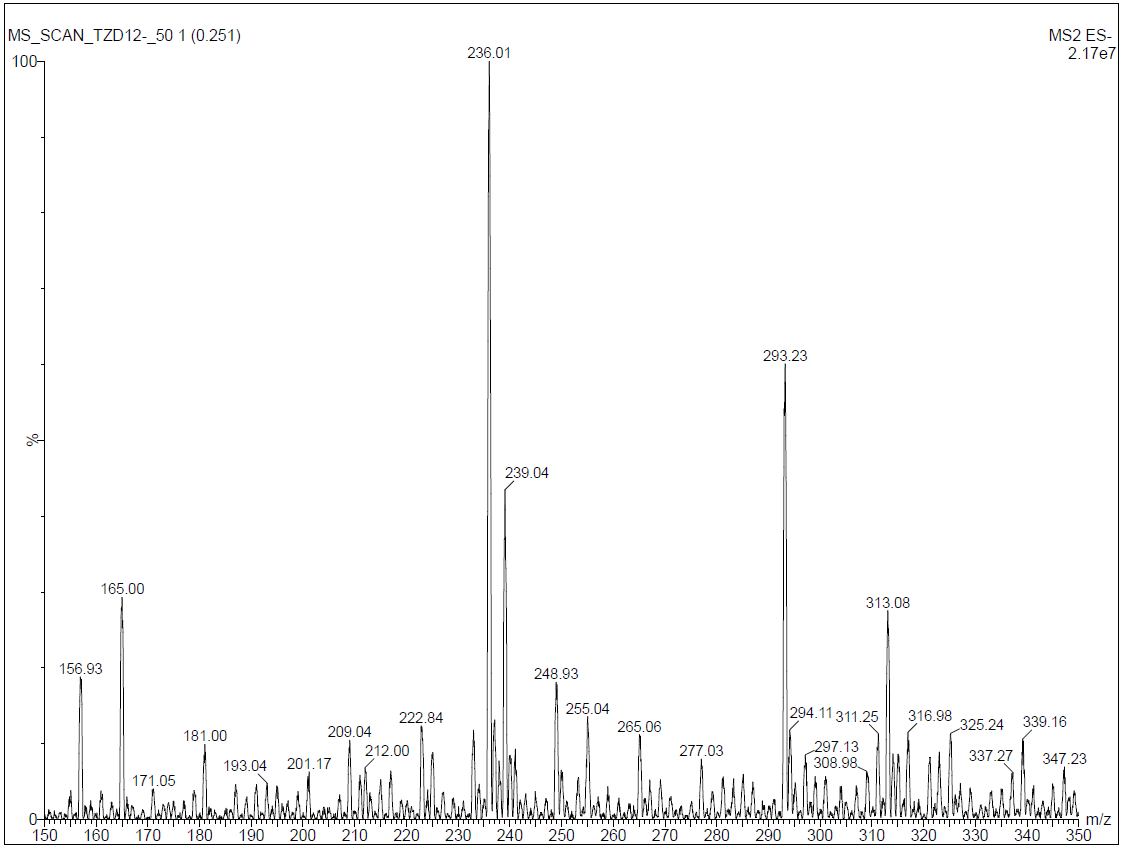


**^1^H NMR spectrum (1f)**


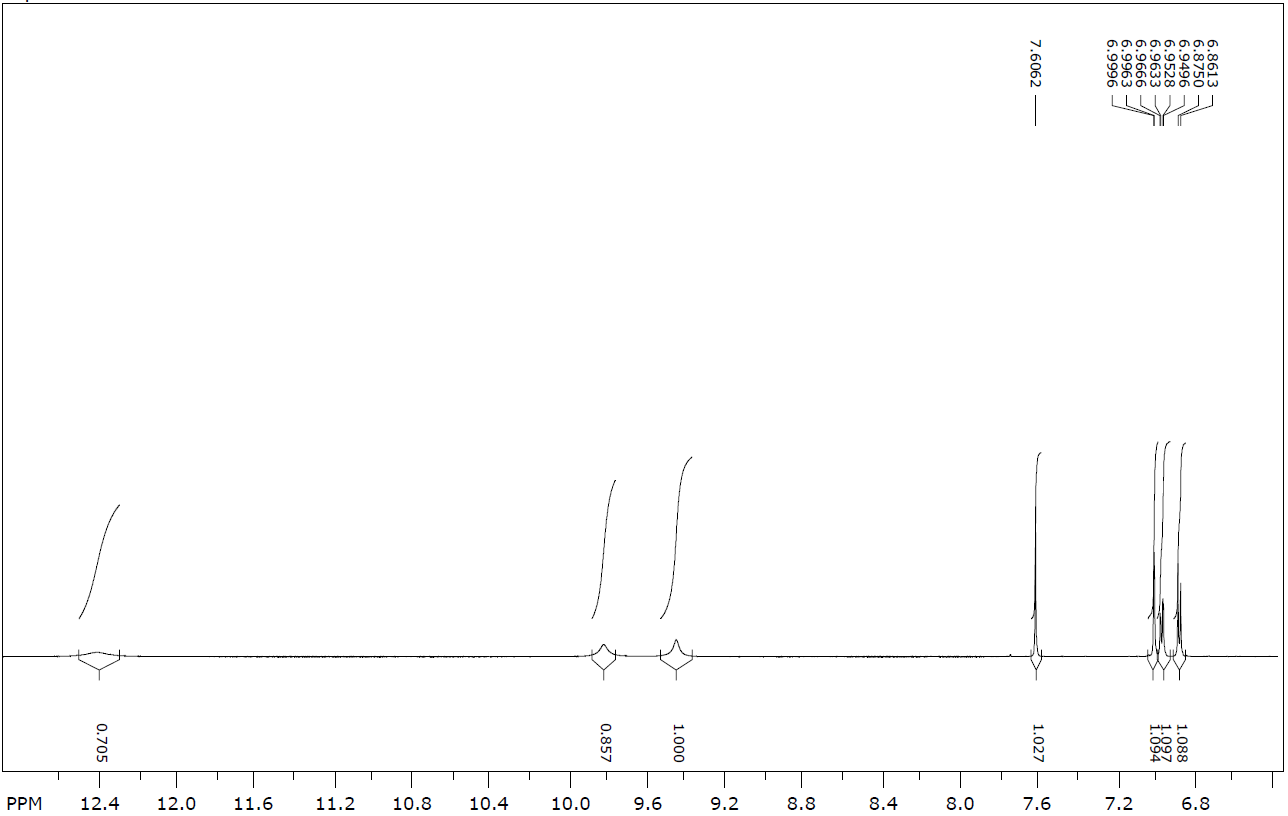


**^13^C NMR spectrum (1f)**


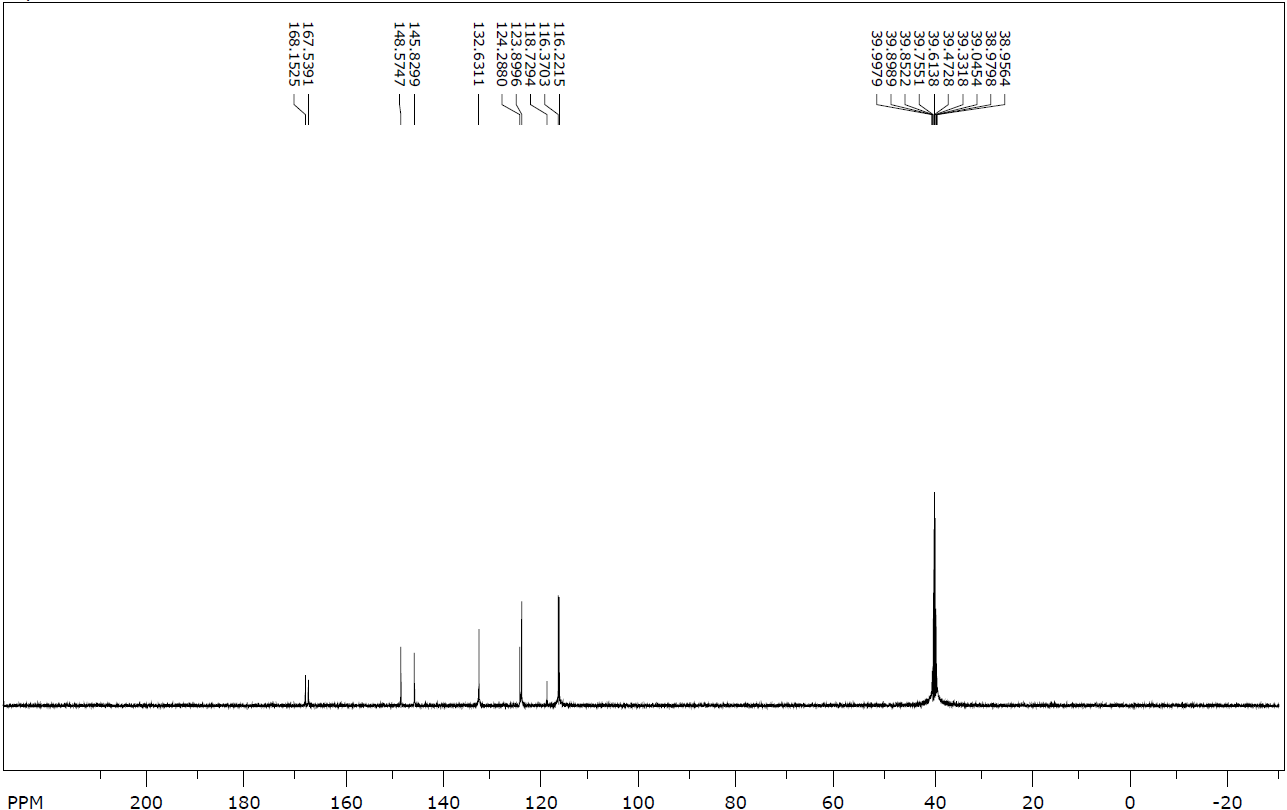


**5-(3,4,5-trimethoxybenzylidene)thiazolidine-2,4-dione (1g)**

Using thiazolidinedione (0.234 g, 2 mmol) and 3,4,5-trimethoxybenzaldehyde (0.392 g, 2 mmol), in accordance with the General Procedure, the title compound **1g** was obtained (0.341 g, 57.8 % yield) as a yellow solid (m.p. 172 – 174 °C). **^1^H** (600 MHz) δ 12.59 (s, 1H, NH), 7.72 (s, 1H, CH), 6.88 (s, 2H, arom.), 3.80 (s, 6H, OCH_3_), 3.70 (s, 3H, OCH_3_). **^13^C** (150 MHz) δ 167.81; 167.20. 155.15; 139.35; 132.05; 128.49; 122.41; 107.48; 60.16; 55.96.

**Mass spectrum (1g)**


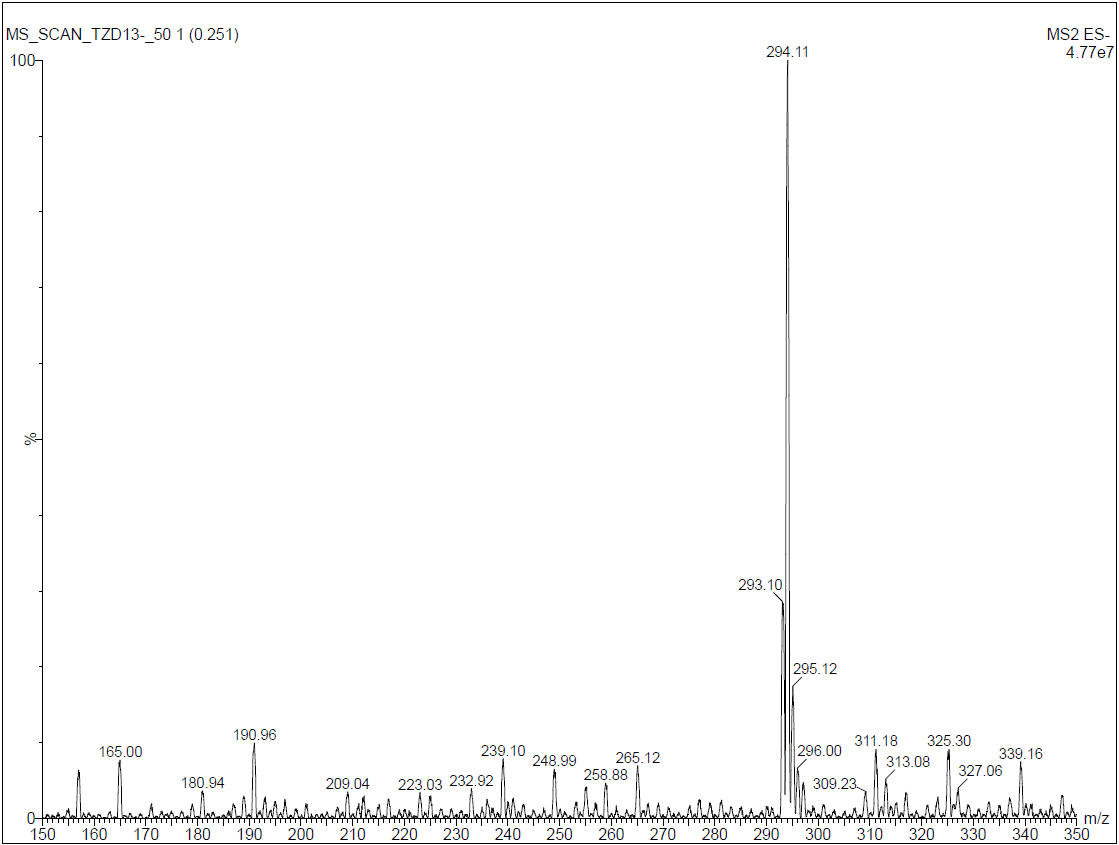


**^1^H NMR spectrum (1g)**


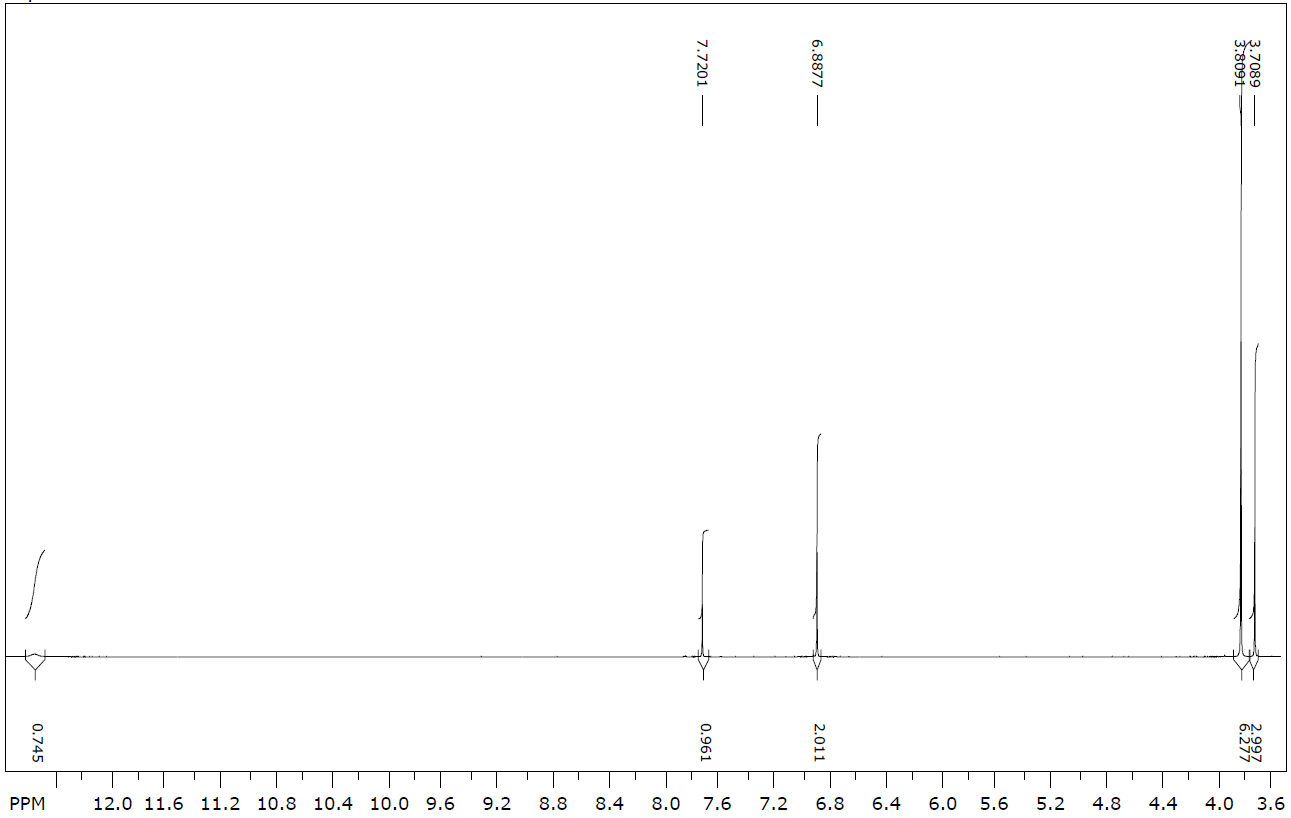


**^13^C NMR spectrum (1g)**


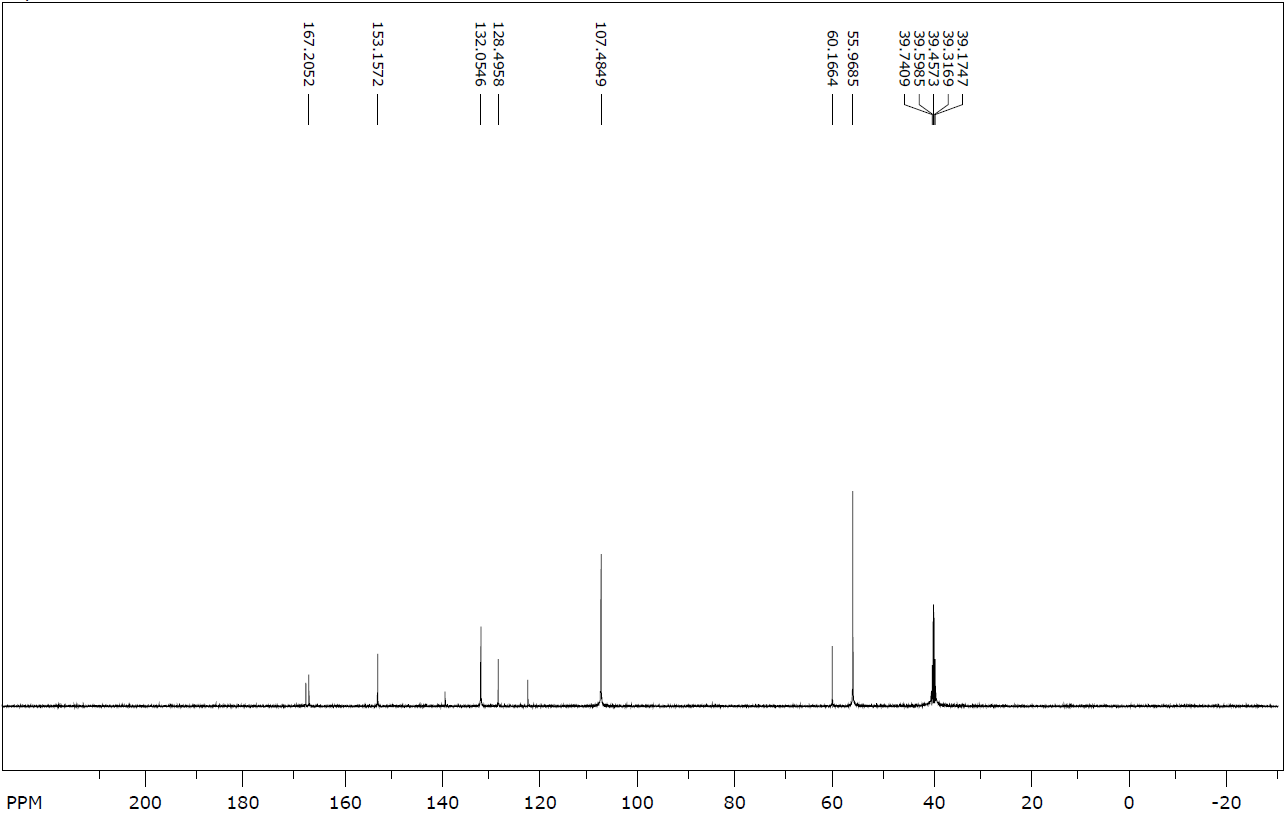


**5-(2,5-dimethoxybenzylidene)thiazolidine-2,4-dione (1h)**

Using thiazolidinedione (0.234 g, 2 mmol) and 2,5-dimethoxybenzaldehyde (0.332 g, 2 mmol), in accordance with the General Procedure, the title compound **1h** was obtained (0.432 g, 81.3 % yield) as a yellow solid (m.p. 220 – 223 °C). **^1^H** (600 MHz) δ 12.56 (s, 1H, NH), 7.90 (s, 1H, CH), 7.07 (t, *J* = 2.64 Hz, 2H, arom.), 6.89 (d, *J* = 2.40 Hz, 1H, arom.), 3.82 (s, 3H, OCH_3_), 3.74 (s, 3H, OCH_3_). **^13^C** (150 MHz) δ 167.91; 167.28; 153.01; 152.36; 126.35; 123.79; 121.90; 117.57. 113.22; 113.00; 56.06: 55.47.

**Mass spectrum (1h)**


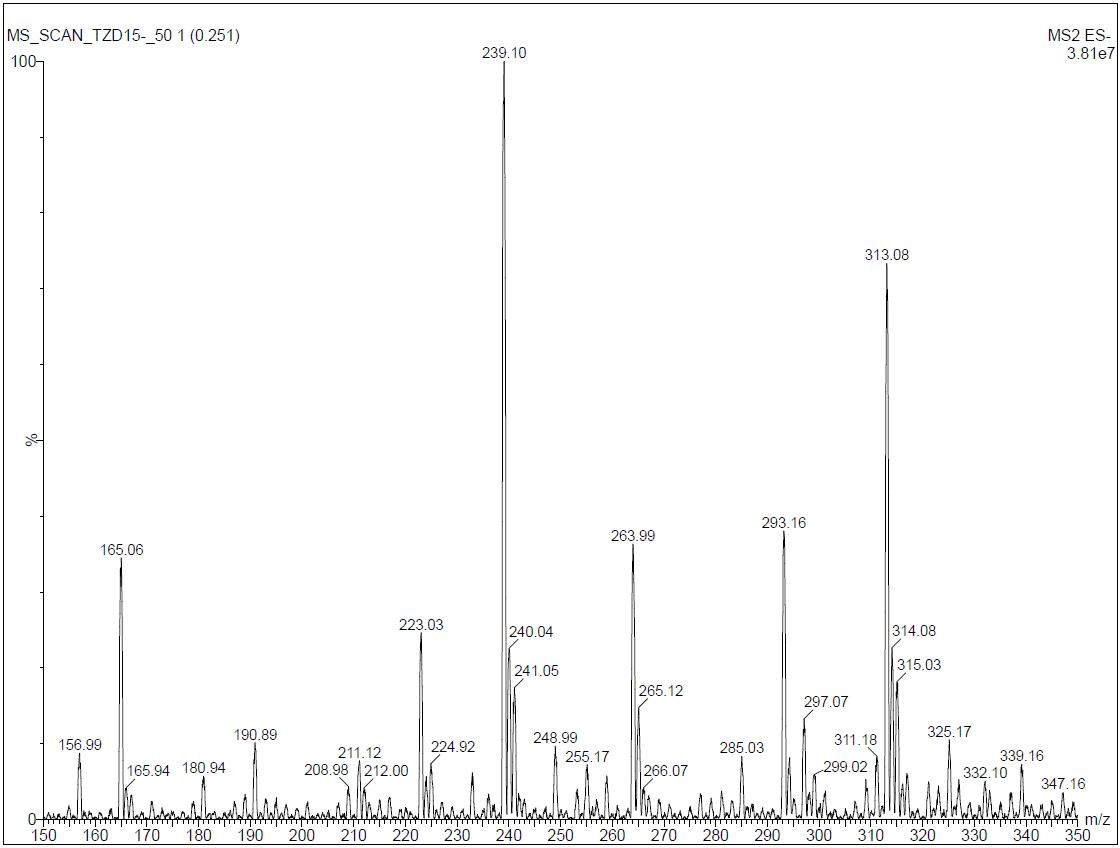


**^1^H NMR spectrum (1h)**


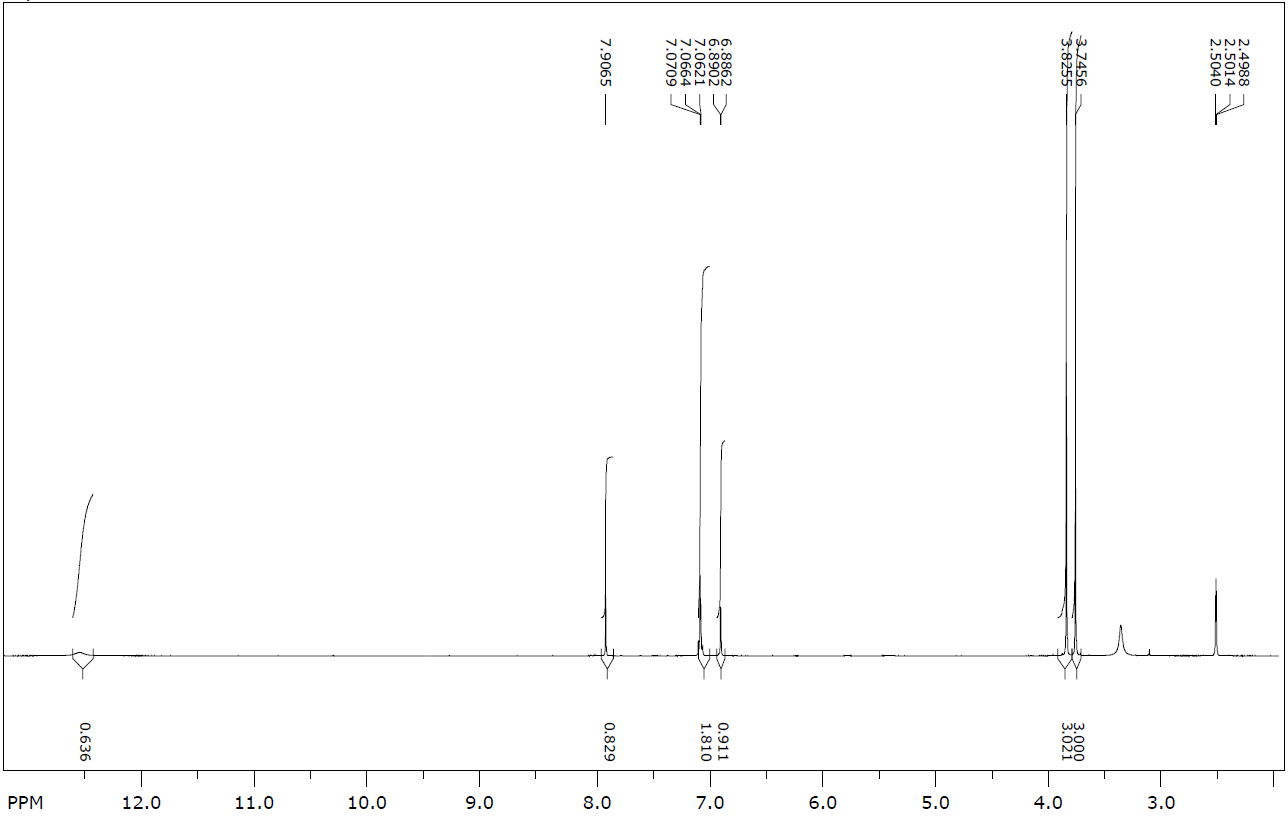


**^13^C NMR spectrum (1h)**


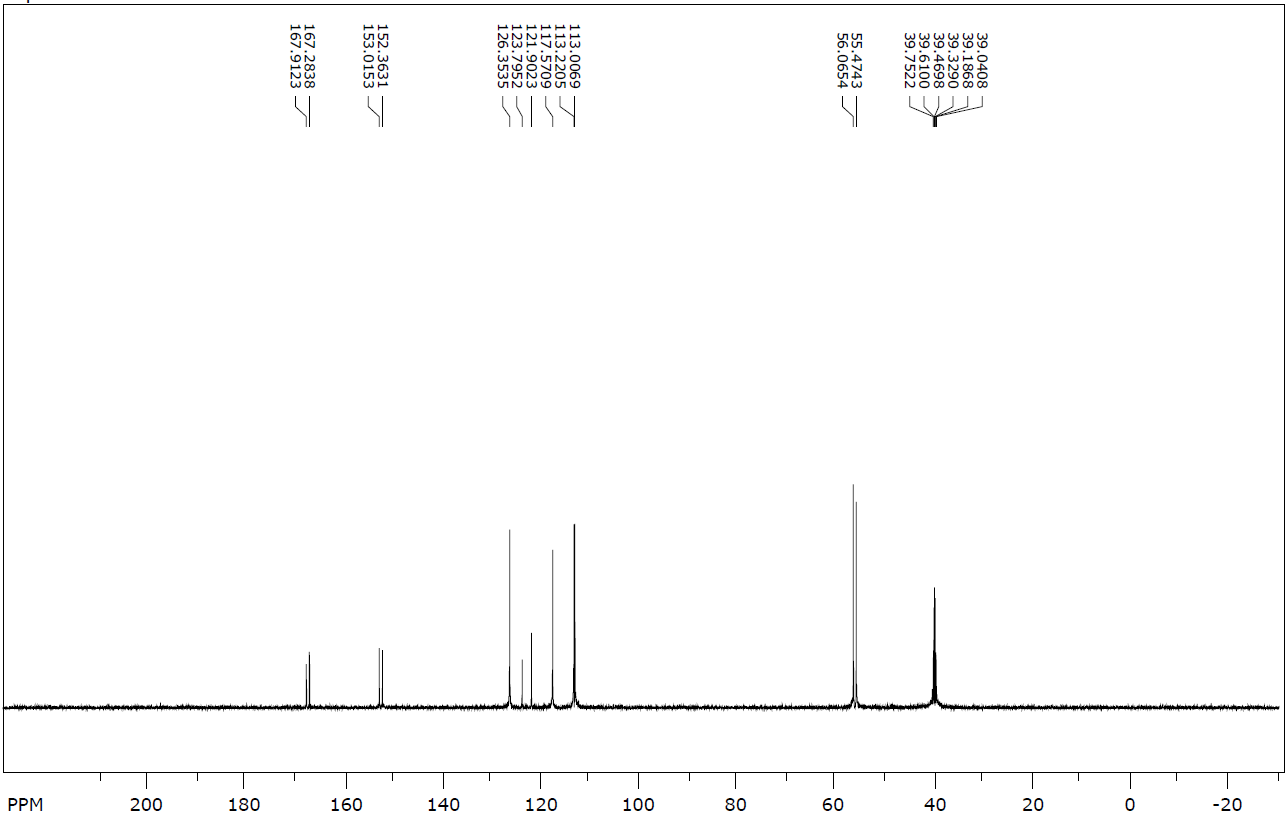


**5-(3-bromobenzylidene)thiazolidine-2,4-dione (1i)**

Using thiazolidinedione (0.234 g, 2 mmol) and 3-bromobenzaldehyde (235 µl, 2 mmol), in accordance with the General Procedure, the title compound **1i** was obtained (0.397 g, 69.8 % yield) as a white solid (m.p. 210 – 212 °C). **^1^H** (300 MHz) δ 12.69 (s, 1H, NH), 7.82 (t, *J* = 1.56 Hz, 1H, arom.), 7.78 (s, 1H, CH), 7.68 (m, 1H, arom.), 7.59 (d, *J* = 7.86 Hz, 1H, arom.), 7.50 (t, *J* = 7.82 Hz, 1H, arom.). **^13^C** (150 MHz) δ 168.05; 167.68; 135.98; 133.31; 133.23; 131.79; 130.46; 128.55; 125.92; 122.90.

**Mass spectrum (1i)**


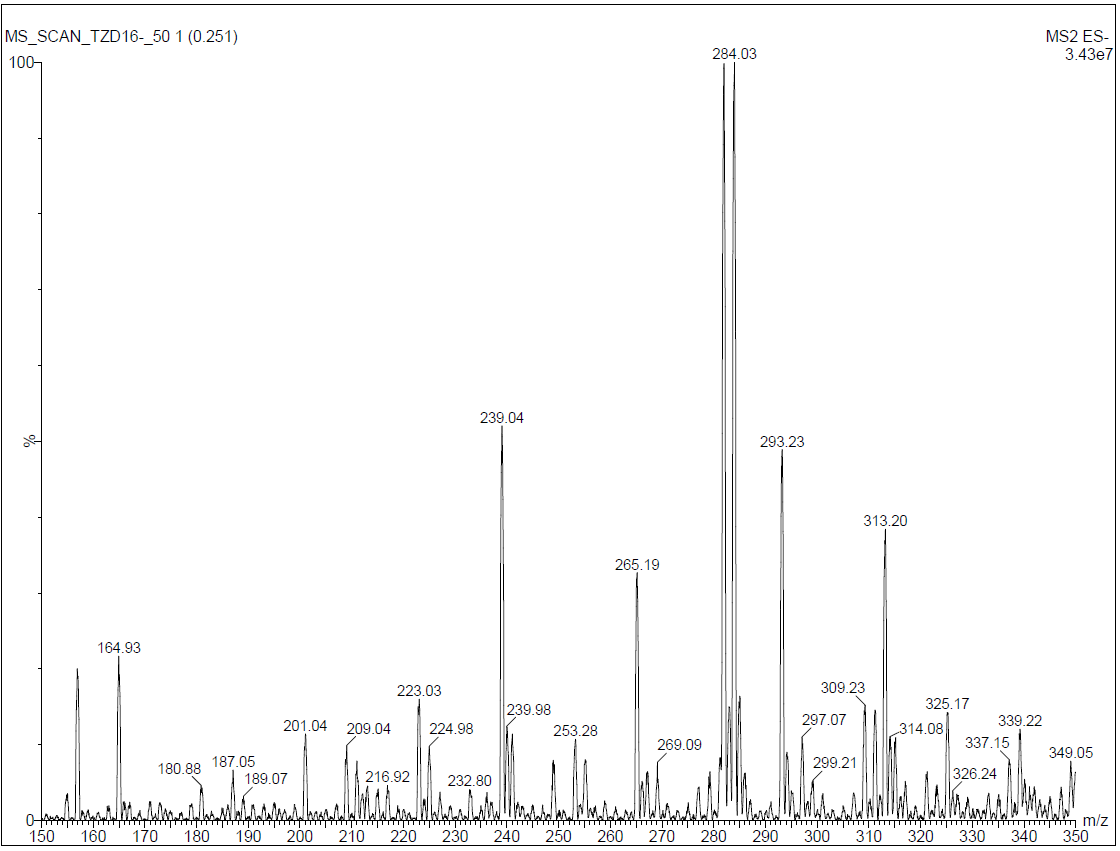


**^1^H NMR spectrum (1i)**


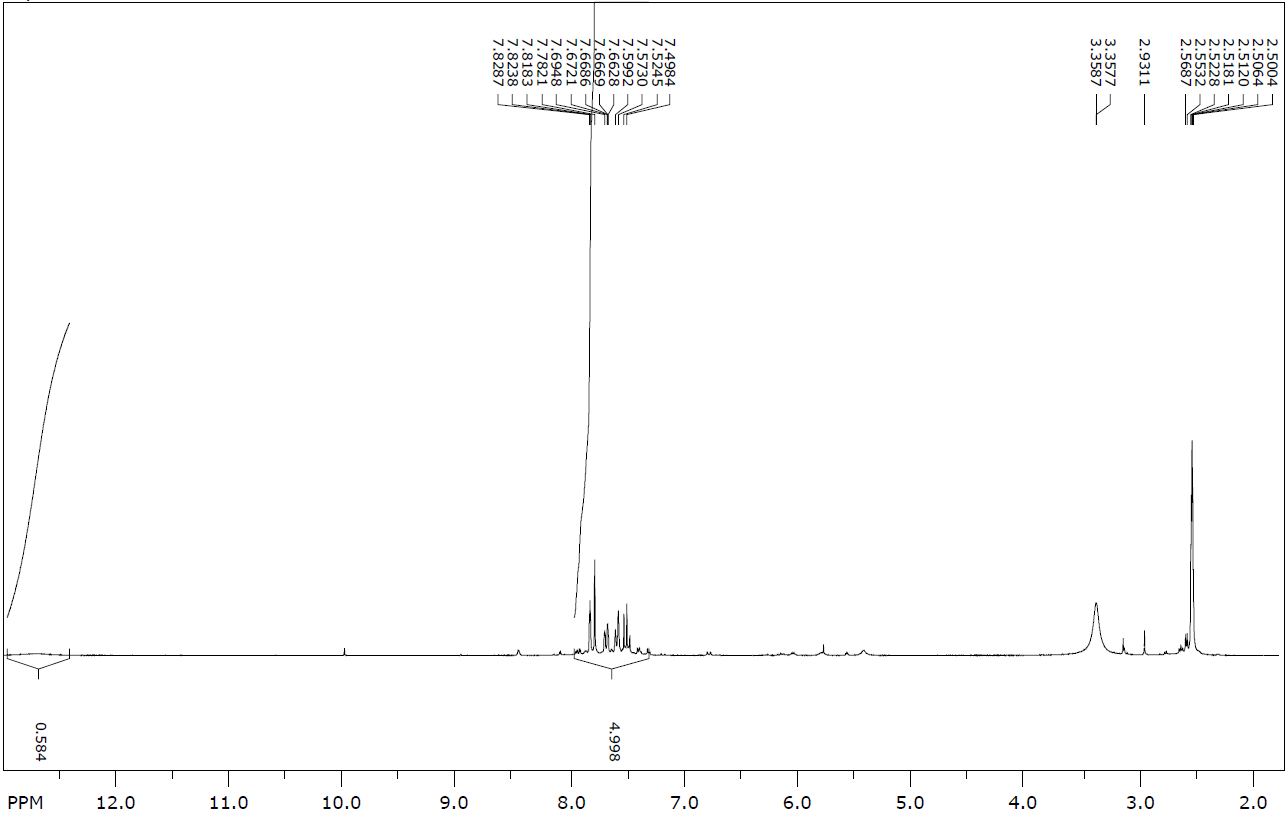


**^13^C NMR spectrum (1i)**


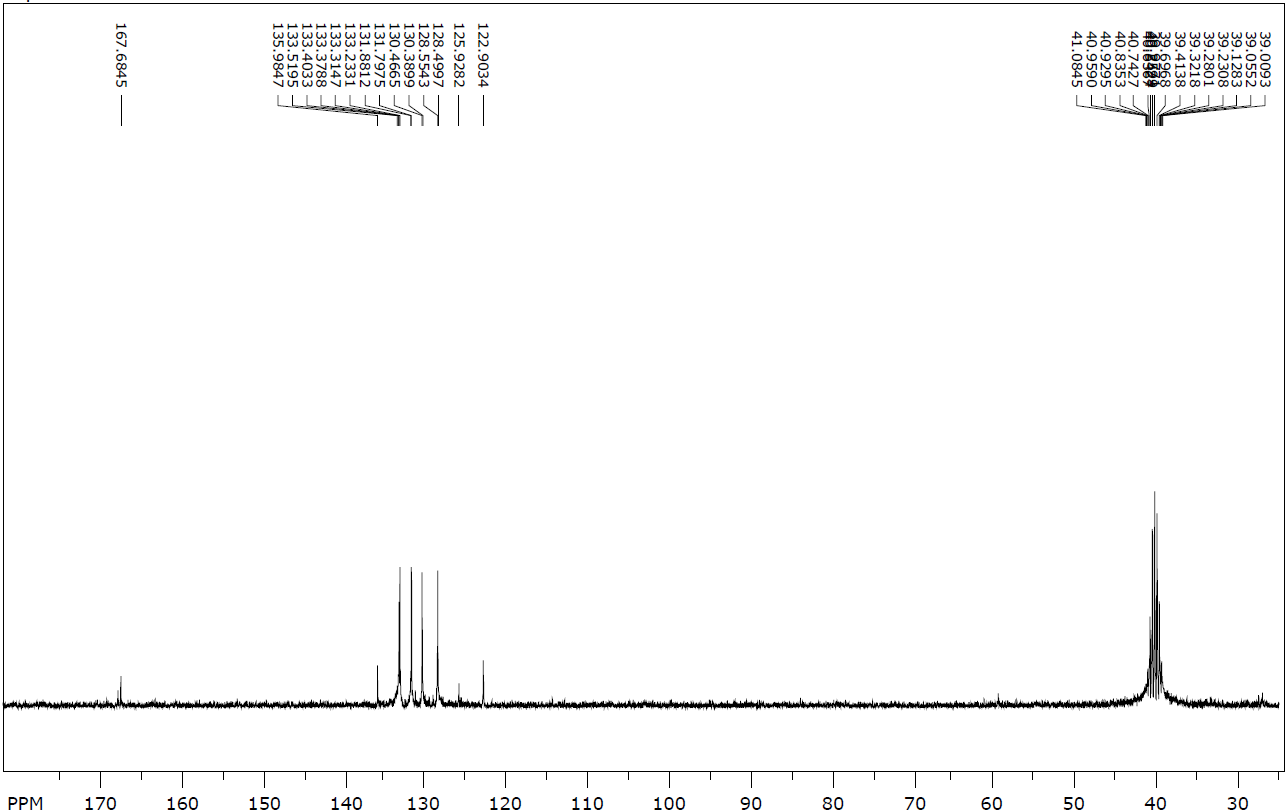


**5-(2-hydroxy-5-nitrobenzylidene)thiazolidine-2,4-dione (1j)**

Using thiazolidinedione (0.234 g, 2 mmol) and 5-nitrosalicylaldehyde (0.334 g, 2 mmol), in accordance with the General Procedure, the title compound **1j** was obtained (0.334 g, 62.8 % yield) as a red solid (m.p. 226 – 228 °C). **^1^H** (600 MHz) δ 8.40 (s, 1H, OH), 8.17 (d, *J* = 2.82 Hz, 1H, arom.), 8.04 (dd, *J* = 9.24; 2.82 Hz, 1H, arom.), 7.85 (s, 1H, arom.), 6.80 (d, *J* = 9.18 Hz, 1H, arom.). **^13^C** (150 MHz) δ 169.06; 127.19; 125.27; 125.07; 121.01; 117.88.

**Mass spectrum (1j)**


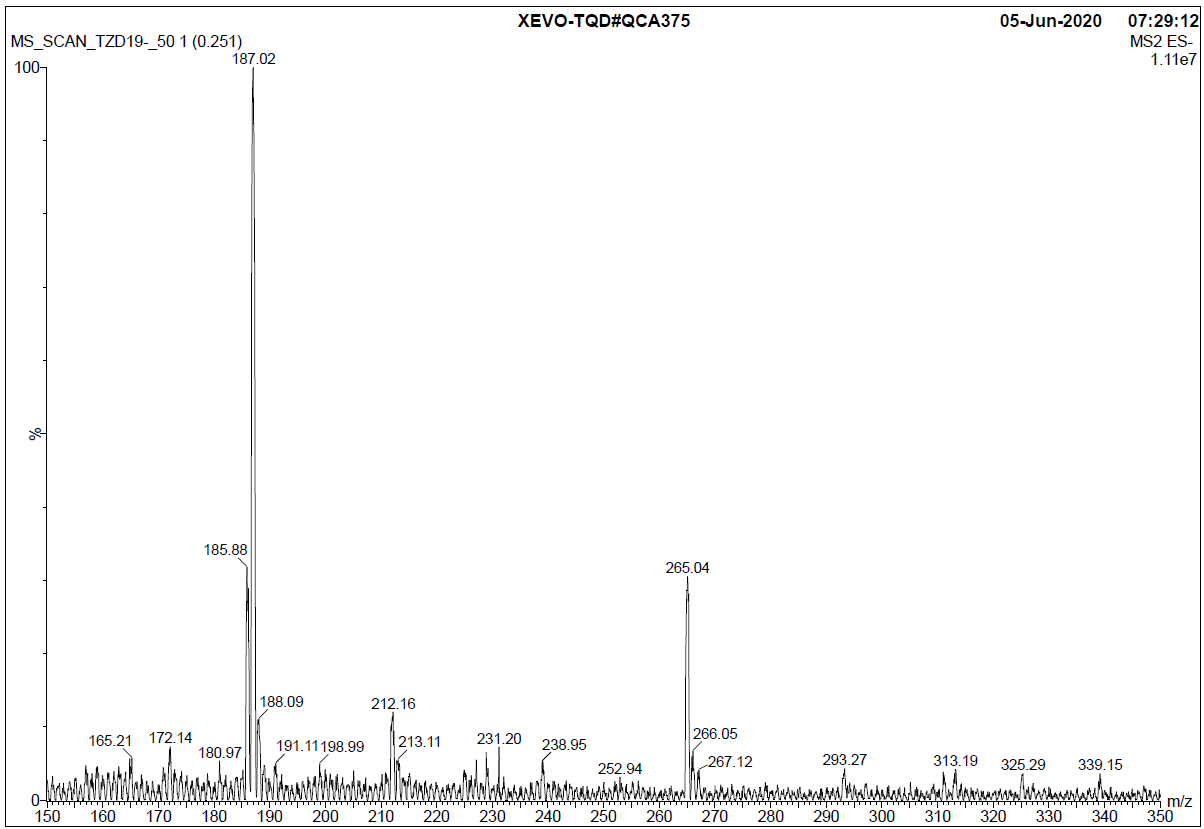


**^1^H NMR spectrum (1j)**


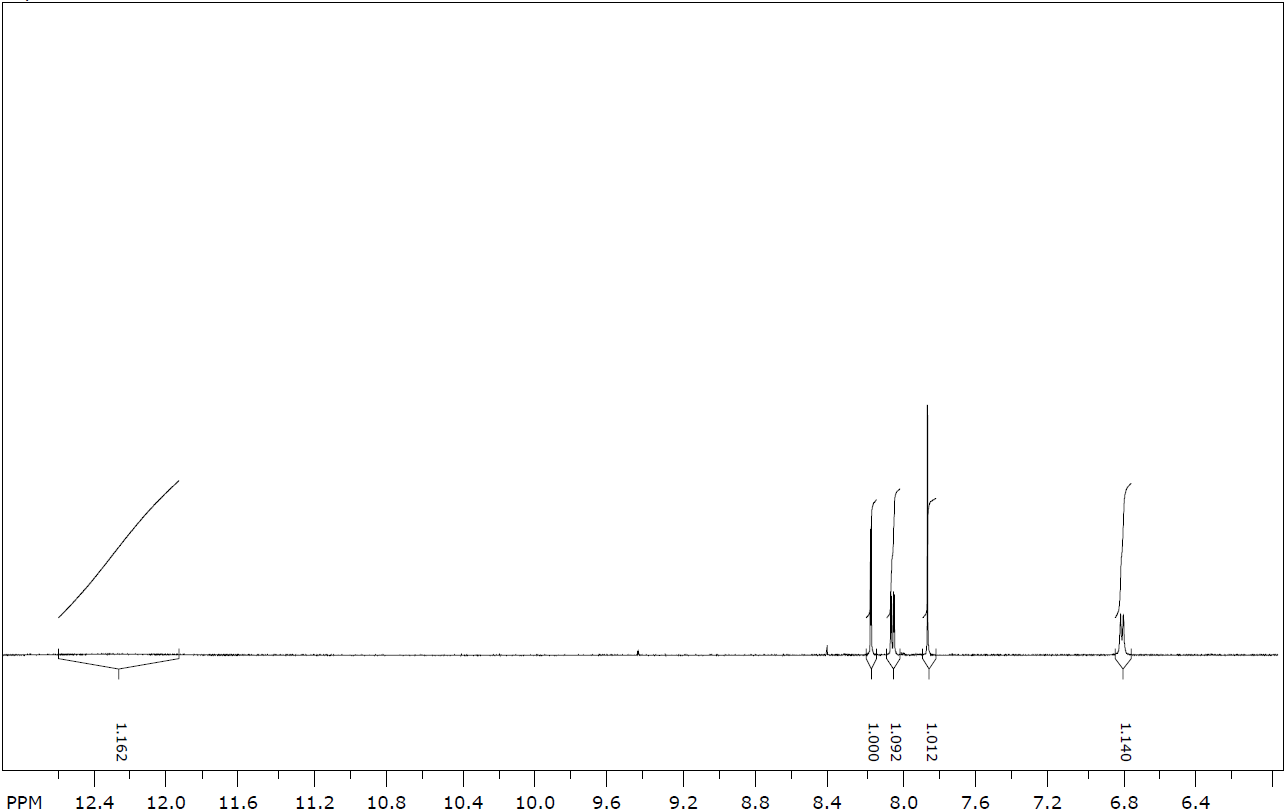


**^13^C NMR spectrum (1j)**


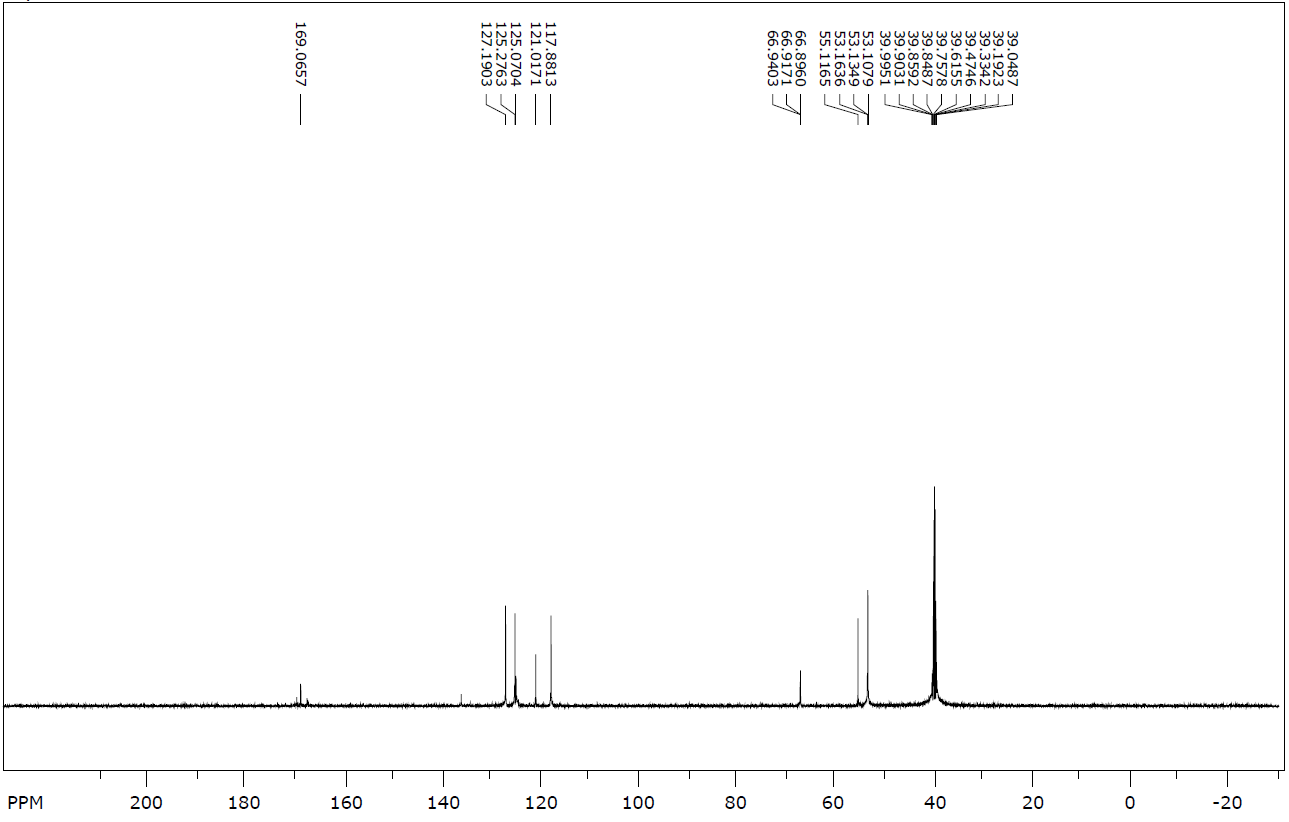


**5-(2-methoxybenzylidene)thiazolidine-2,4-dione (1k)**

Using thiazolidinedione (0.234 g, 2 mmol) and 2-methoxybenzaldehyde (0.241 µl, 2 mmol), in accordance with the General Procedure, the title compound **1k** was obtained (0.334 g, 71.0 % yield) as a yellow solid (m.p. 240 – 241 °C). **^1^H** (600 MHz) δ 12.54 (s, 1H, NH), 7.95 (s, 1H, CH), 7.45 – 7.48 (m, 1H, arom.), 7.39 (dd, *J* = 7.74; 1.38 Hz, 1H, arom.), 7.14 (d, *J* = 8.22 Hz, 1H, arom.), 7.08 (t, *J* = 7.53 Hz, 1H, arom.), 3.87 (s, 3H, OCH_3_). **^13^C** (150 MHz) δ 168.09; 167.41; 132.36; 128.52; 126.42; 123.43; 121.41. 120.90; 111.82; 55.73.

**Mass spectrum (1k)**


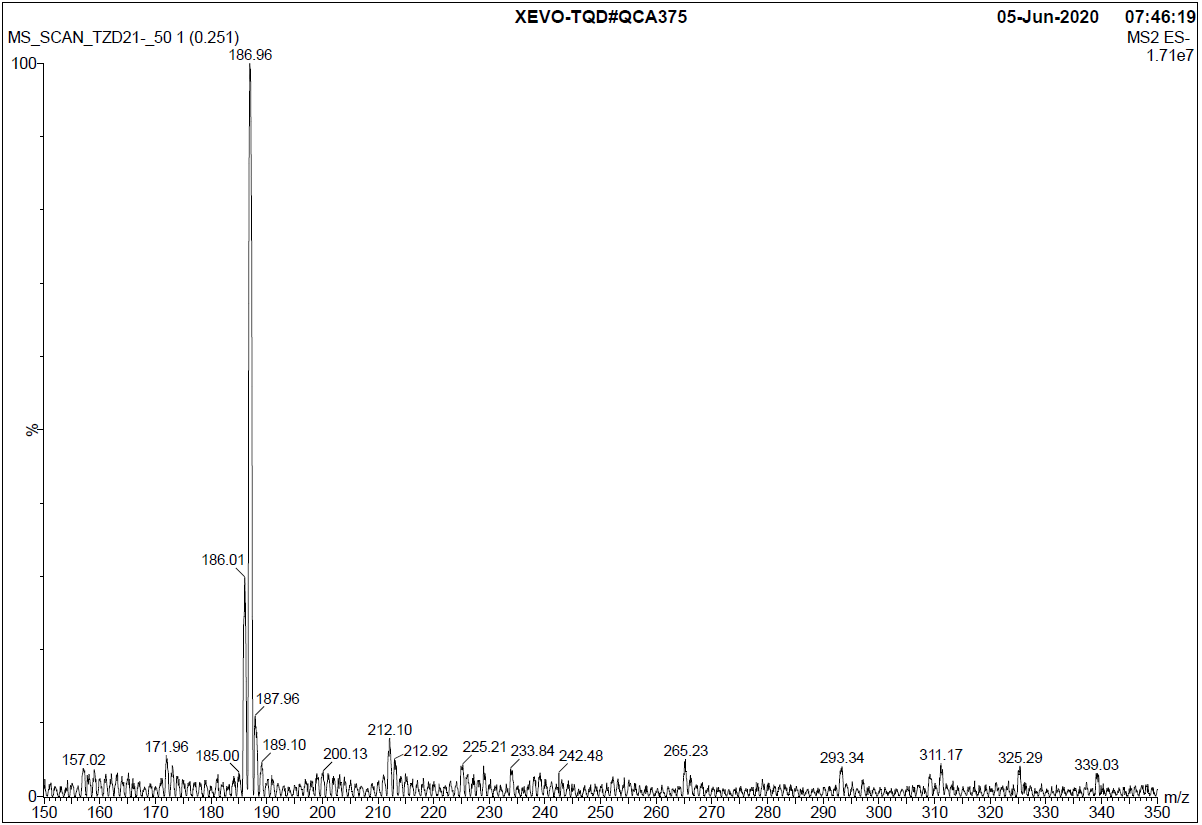


**^1^H NMR spectrum (1k)**


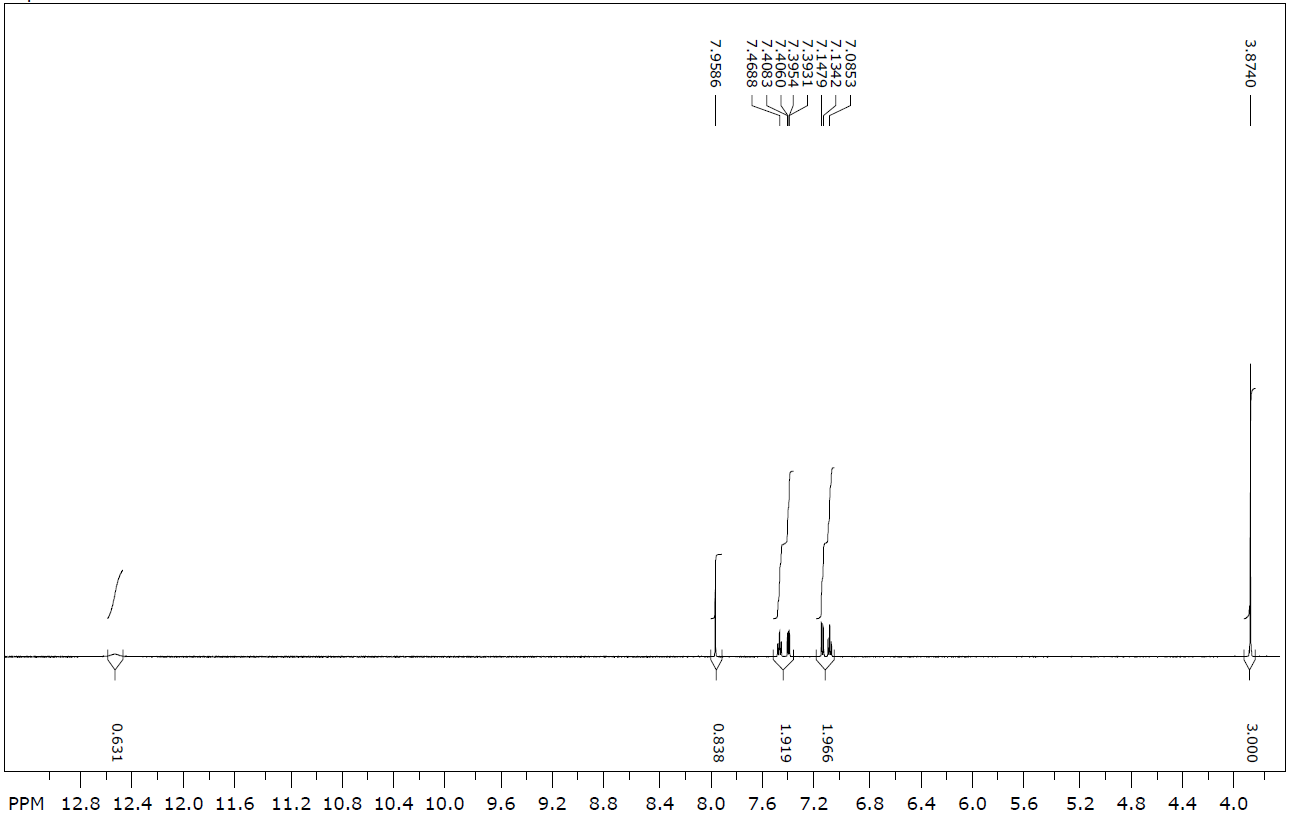


**^13^C NMR spectrum (1k)**


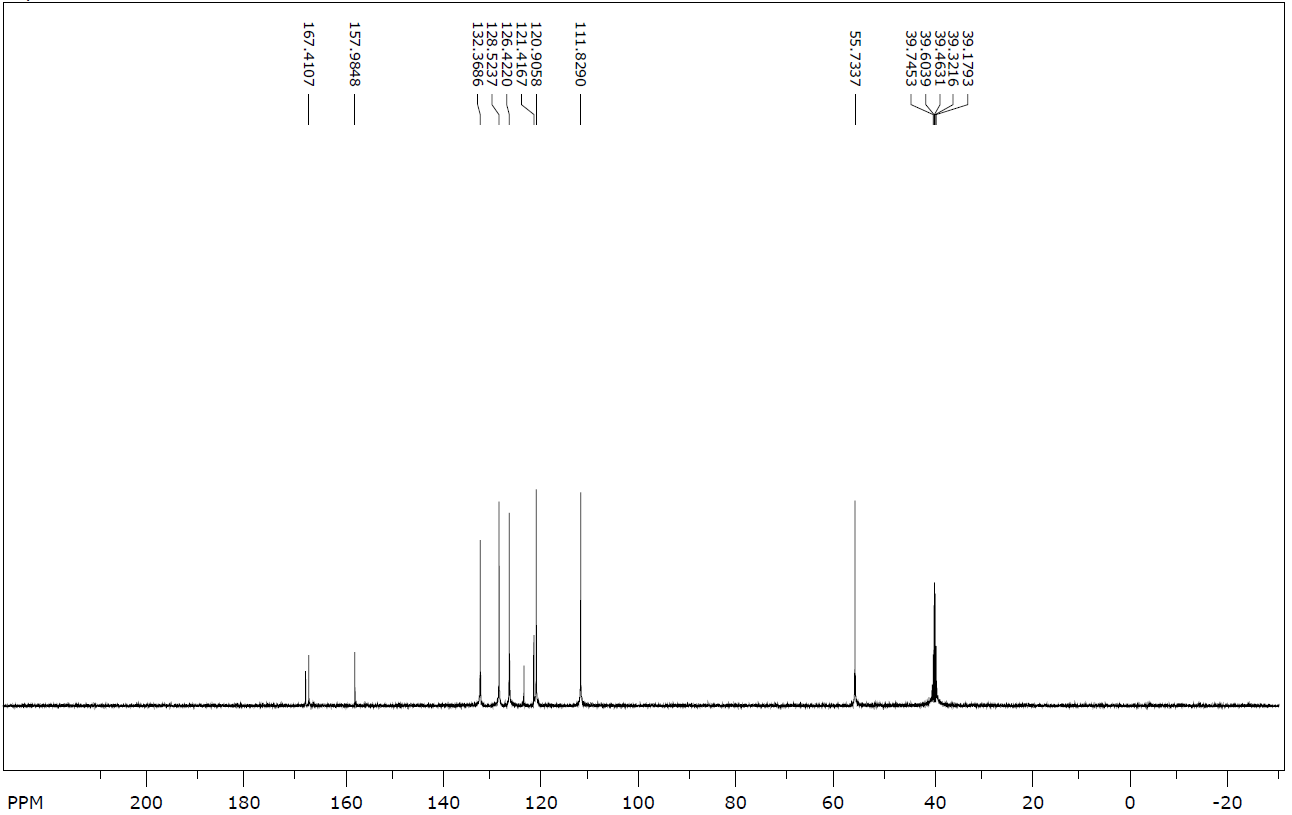


**5-(3-hydroxybenzylidene)thiazolidine-2,4-dione (1l)**

Using thiazolidinedione (0.234 g, 2 mmol) and 3-hydroxybenzaldehyde (0.244 g, 2 mmol), in accordance with the General Procedure, the title compound **1l** was obtained (0.132 g, 29.7 % yield) as a brown solid (m.p. 262 – 264 °C). **^1^H** (300 MHz) δ 12.58 (s, 1H, NH), 9.82 (s, 1H, OH), 7.67 (s, 1H, CH), 7.31 (t, *J* = 7.89 Hz, 1H, arom.), 7.01 (d, *J* = 7.89 Hz, 1H, arom.), 6.96 (s, 1H, arom.), 6.86 (dd, *J* = 8.07; 1.76 Hz, 1H, arom.). **^13^C** (150 MHz) δ 168.42; 167.80; 158.32; 134.65; 132.45. 130.83; 123.75; 121.78; 118.19; 116.37.

**Mass spectrum (1l)**


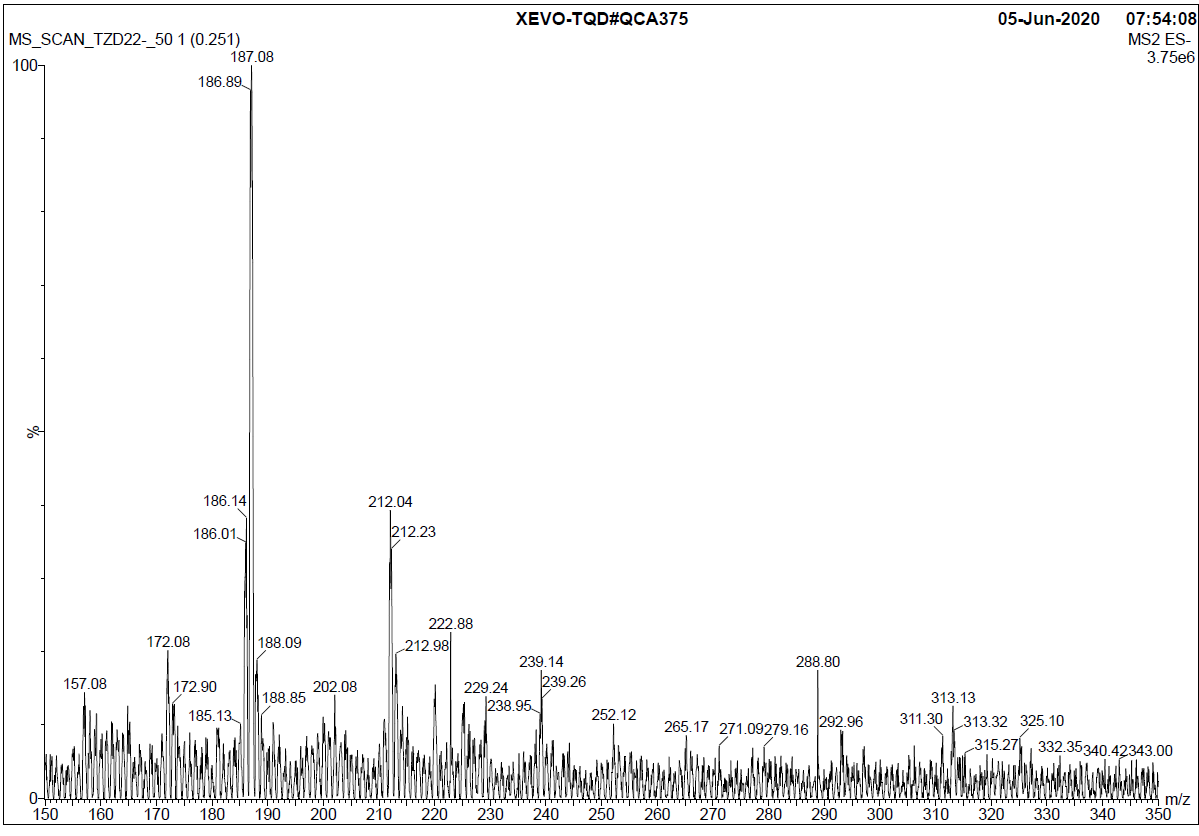


**^1^H NMR spectrum (1l)**


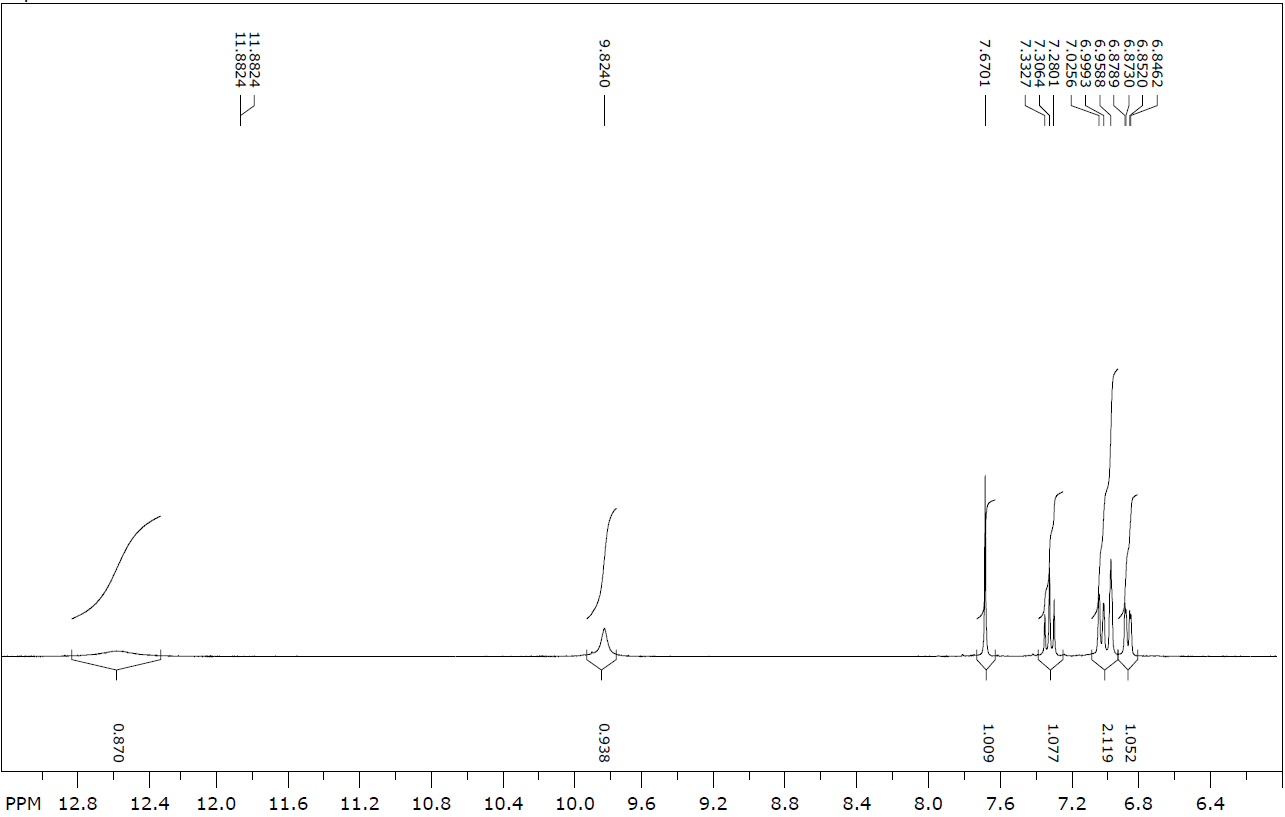


**^13^C NMR spectrum (1l)**


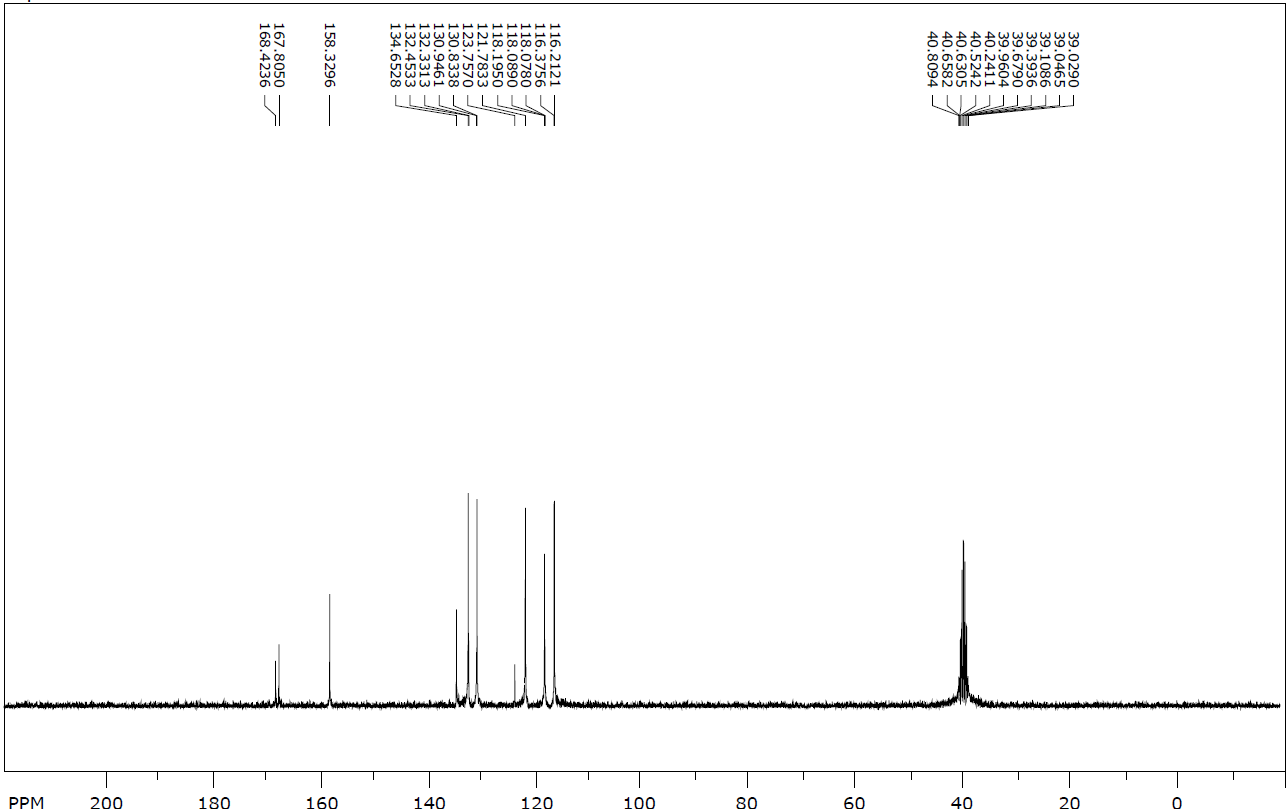


**5-(4-hydroxybenzylidene)thiazolidine-2,4-dione (1m)**

Using thiazolidinedione (0.234 g, 2 mmol) and 4-hydroxybenzaldehyde (0.244 g, 2 mmol), in accordance with the General Procedure, the title compound **1m** was obtained (0.269 g, 60.8 % yield) as a yellow solid (m.p. 296 – 297 °C). **^1^H** (600 MHz) δ 12.43 (s, 1H, NH), 10.30 (s, 1H, OH), 7.69 (s, 1H, CH), 7.45 (d, *J* = 8.58 Hz, 2H, arom.), 6.91 (d, *J* = 8.64 Hz, 2H, arom.). **^13^C** (150 MHz) δ 168.05; 167.52; 159.84; 132.35; 132.24; 123.90; 118.97; 116.28.

**Mass spectrum (1m)**


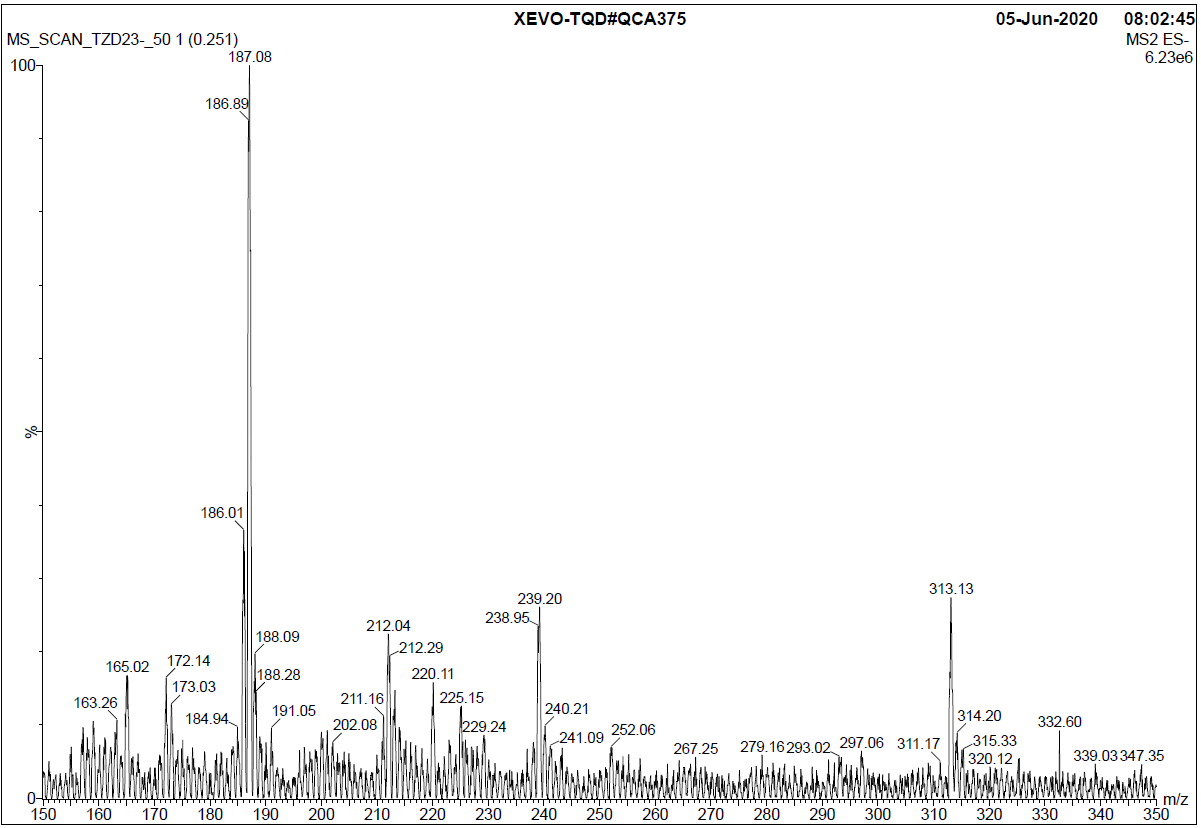


**^1^H NMR spectrum (1m)**


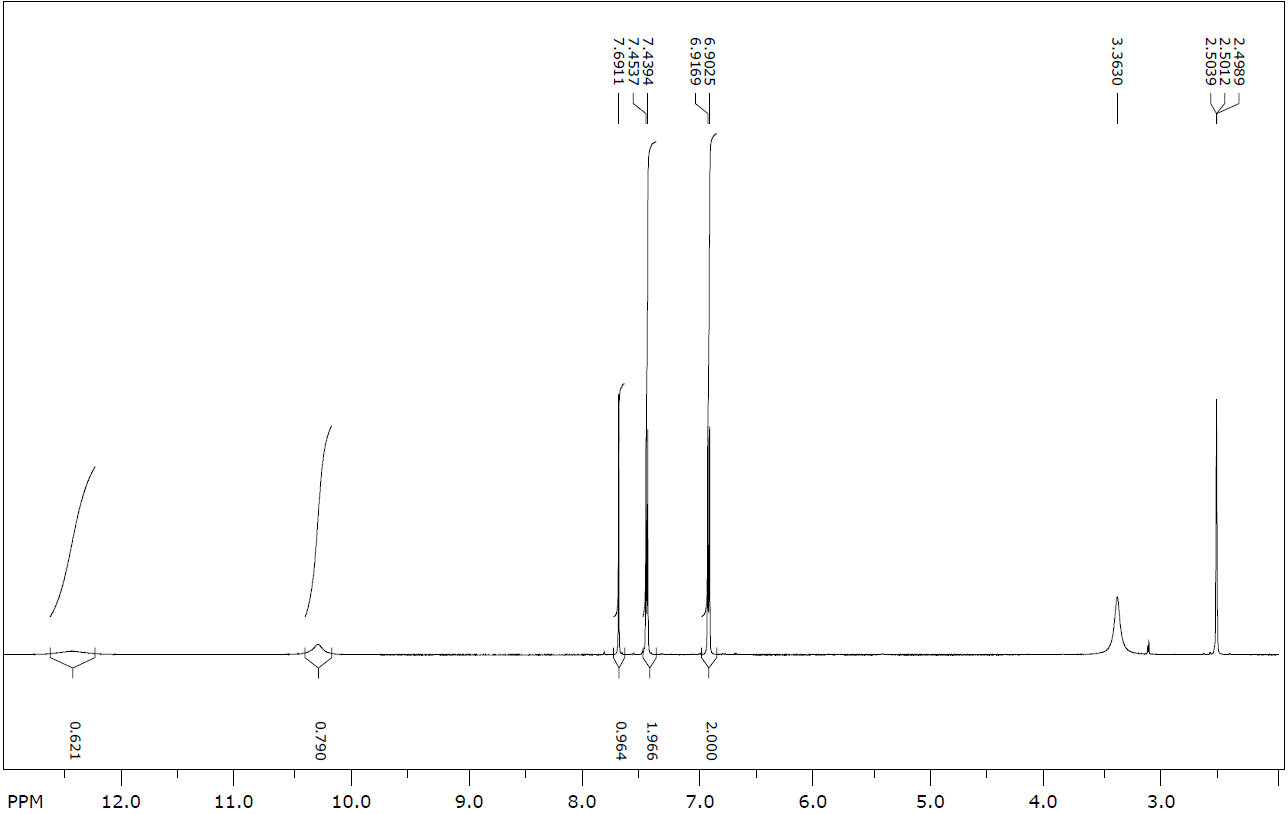


**^13^C NMR spectrum (1m)**


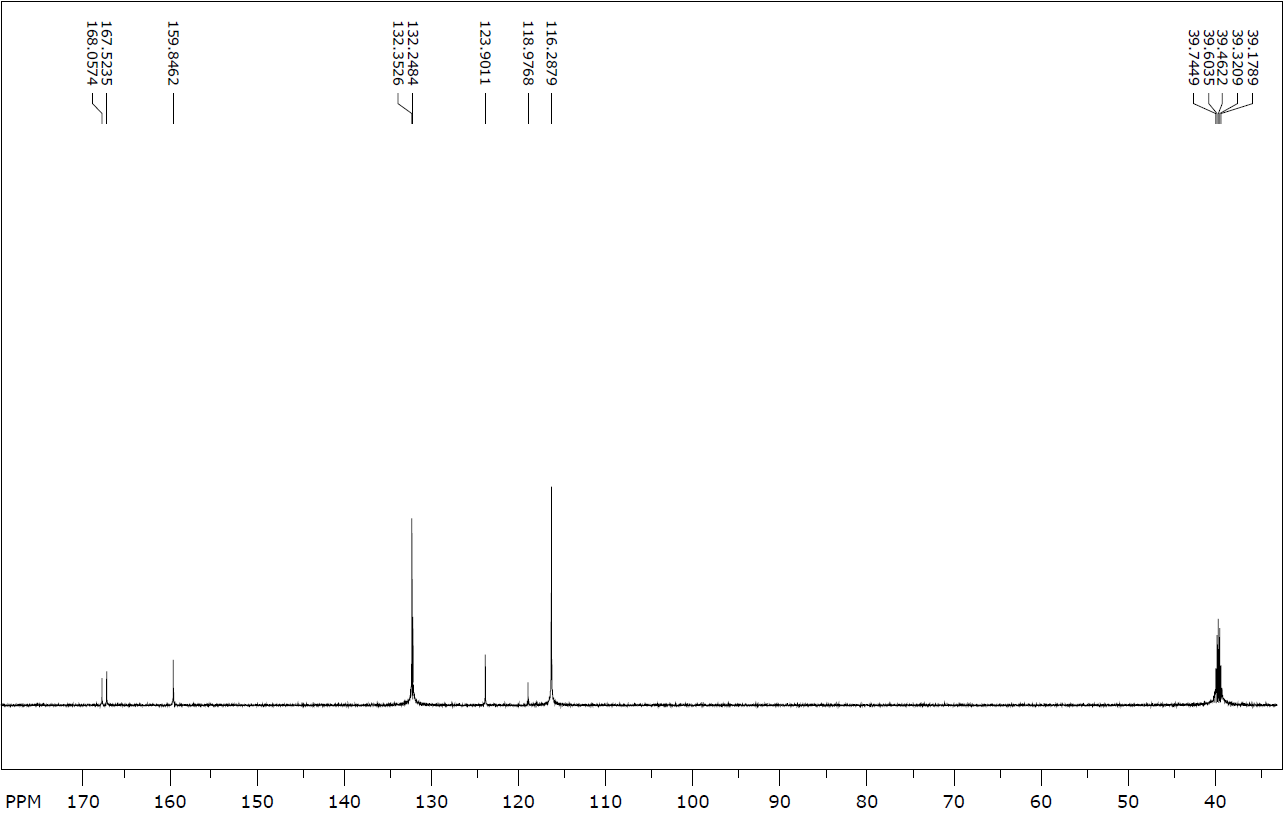


**5-(4-(dimethylamino)benzylidene)thiazolidine-2,4-dione (1n)**

Using thiazolidinedione (0.234 g, 2 mmol) and 4-dimethylaminobenzaldehyde (0.298 g, 2 mmol), in accordance with the General Procedure, the title compound **1n** was obtained (0.451 g, 90.9 % yield) as a orange solid (m.p. 286 – 289 °C). **^1^H** (600 MHz) δ 12.31 (s, 1H, NH), 7.66 (s, 1H, CH), 7.43 (d, *J* = 9.00 Hz, 2H, arom.), 6.82 (d, *J* = 9.00 Hz, 2H, arom.), 3.01 (s, 6H, CH_3_). **^13^C** (150 MHz) δ 168.15; 167.57; 151.39; 132.84; 132.08; 127.75; 119.79; 115.69; 111.99.

**Mass spectrum (1n)**


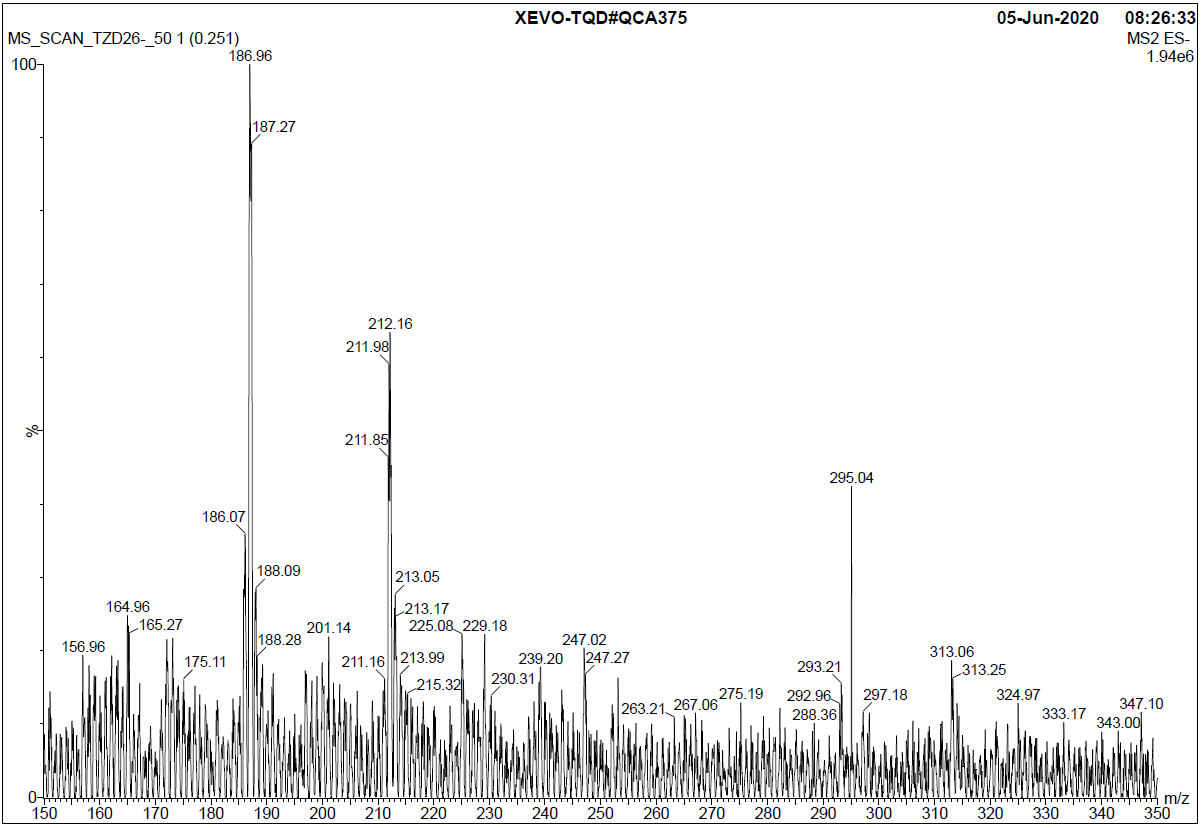


**^1^H NMR spectrum (1n)**


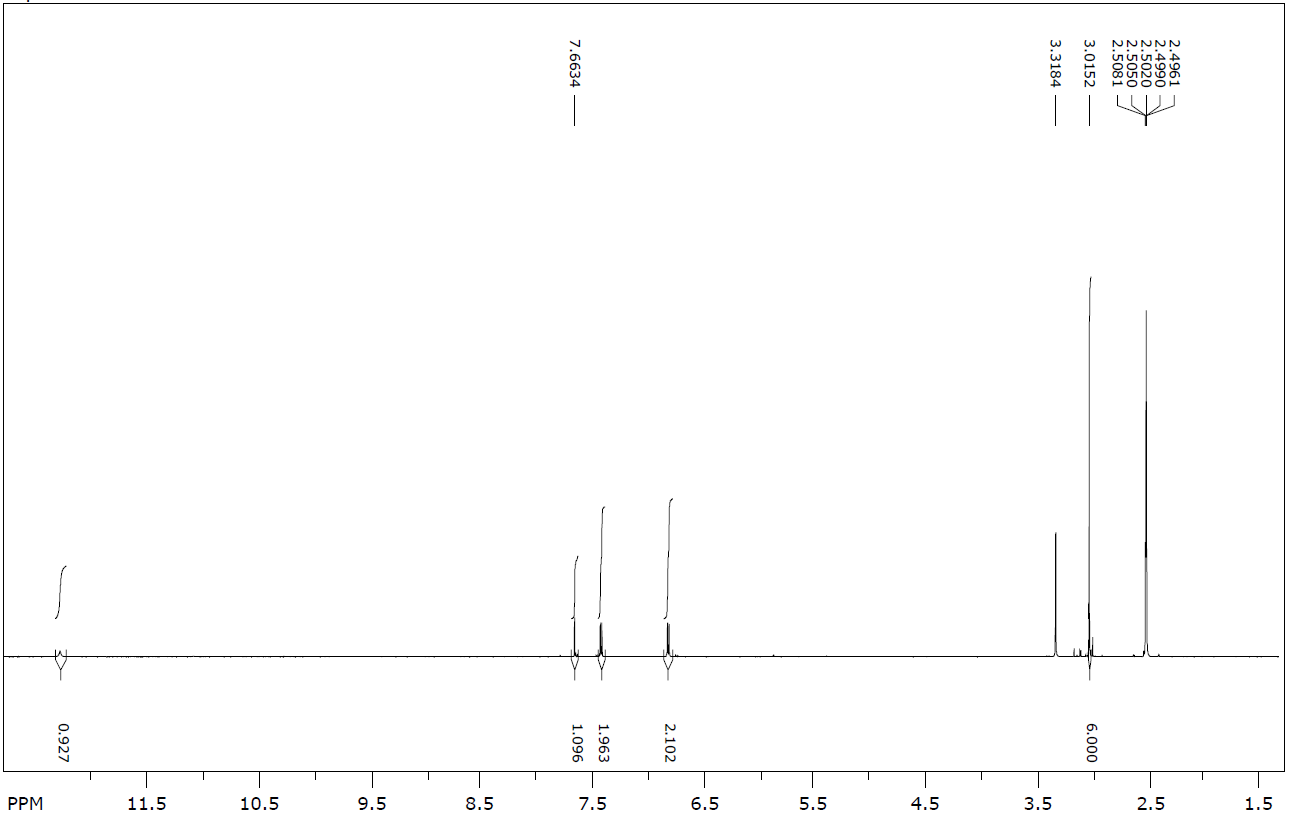


**^13^C NMR spectrum (1n)**


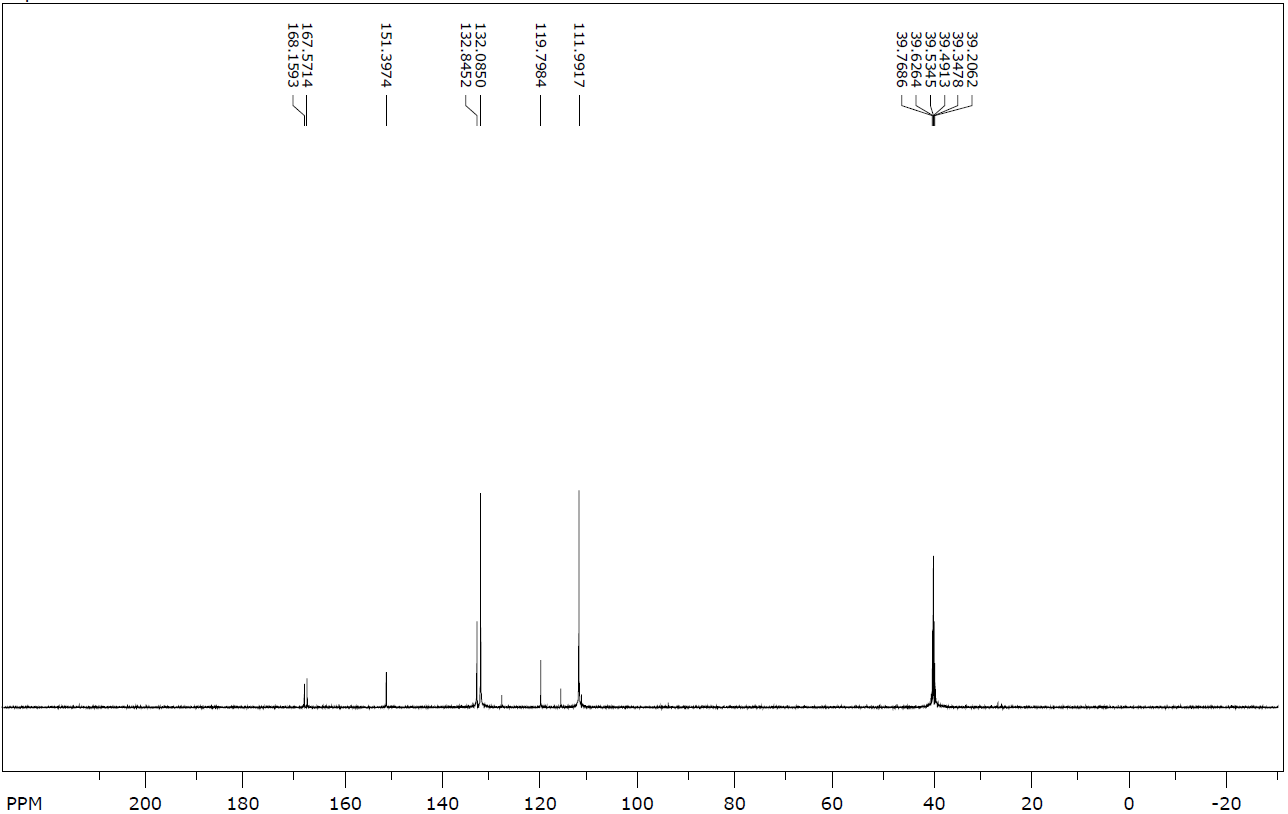


**5-(4-(benzyloxy)-2-hydroxybenzylidene)thiazolidine-2,4-dione (1o)**

Using thiazolidinedione (0.117 g, 1 mmol) and 4-(benzyloxy)salicylaldehyde (0.228 g, 1 mmol), in accordance with the General Procedure, the title compound **1o** was obtained (0.291 g, 88.9 % yield) as a yellow solid (m.p. 184 – 188 °C). **^1^H** (600 MHz) δ 12.39 (s, 1H, NH), 10.54 (s, 1H, OH), 7.91 (s, 1H, CH), 7.44 (d, *J* = 8.46 Hz, 2H, arom), 7.40 (t, *J* = 7.47 Hz, 2H, arom.), 7.35 (m, 1H, arom.), 7.28 (d, *J* = 8.76 Hz, 1H, arom.), 6.65 (dd, *J* = 8.76; 2.46 Hz, 1H, arom.), 6.57 (d, *J* = 2.52 Hz, 1H, arom.), 5.11 (s, 2H, CH_2_). **^13^C** (150 MHz) δ 161.08; 158.70; 136.57; 129.40; 128.44; 127.92; 127.69; 113.99; 106.85; 102.01; 69.28; 55.11; 53.13.

**Mass spectrum (1o)**


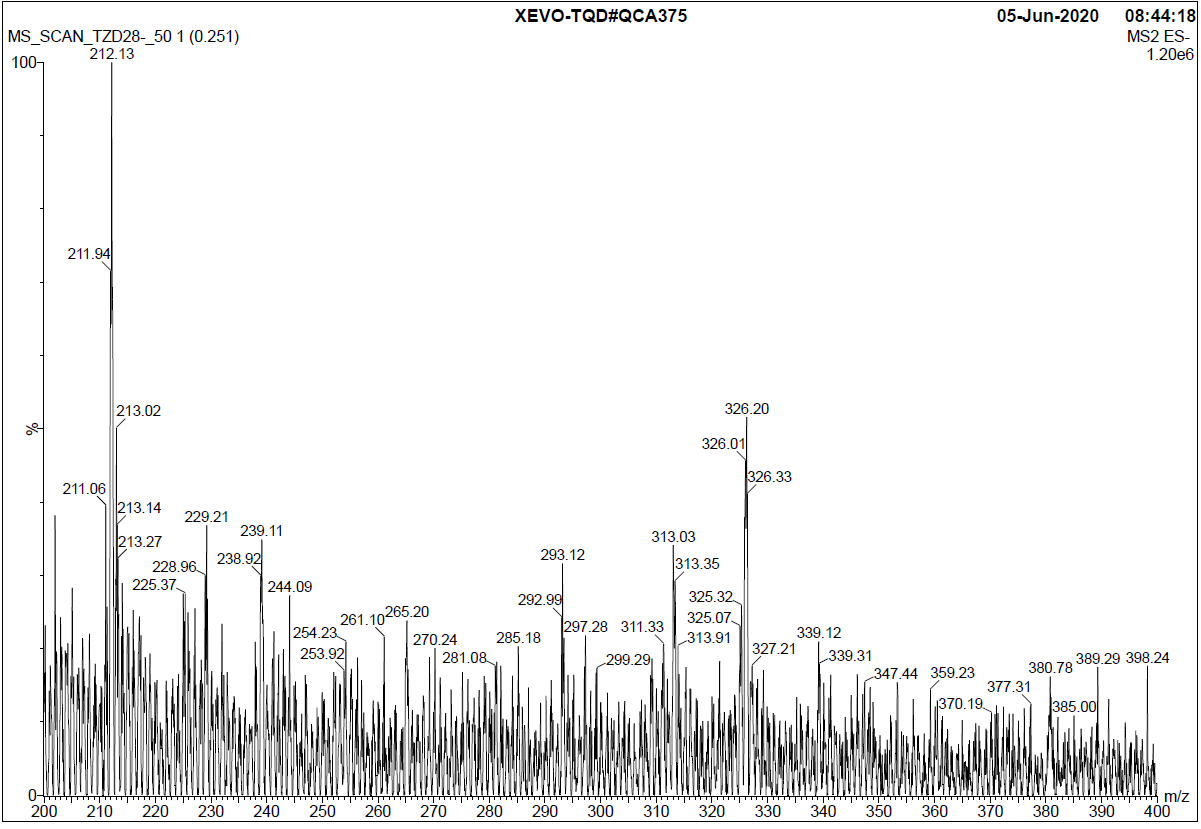


**^1^H NMR spectrum (1o)**


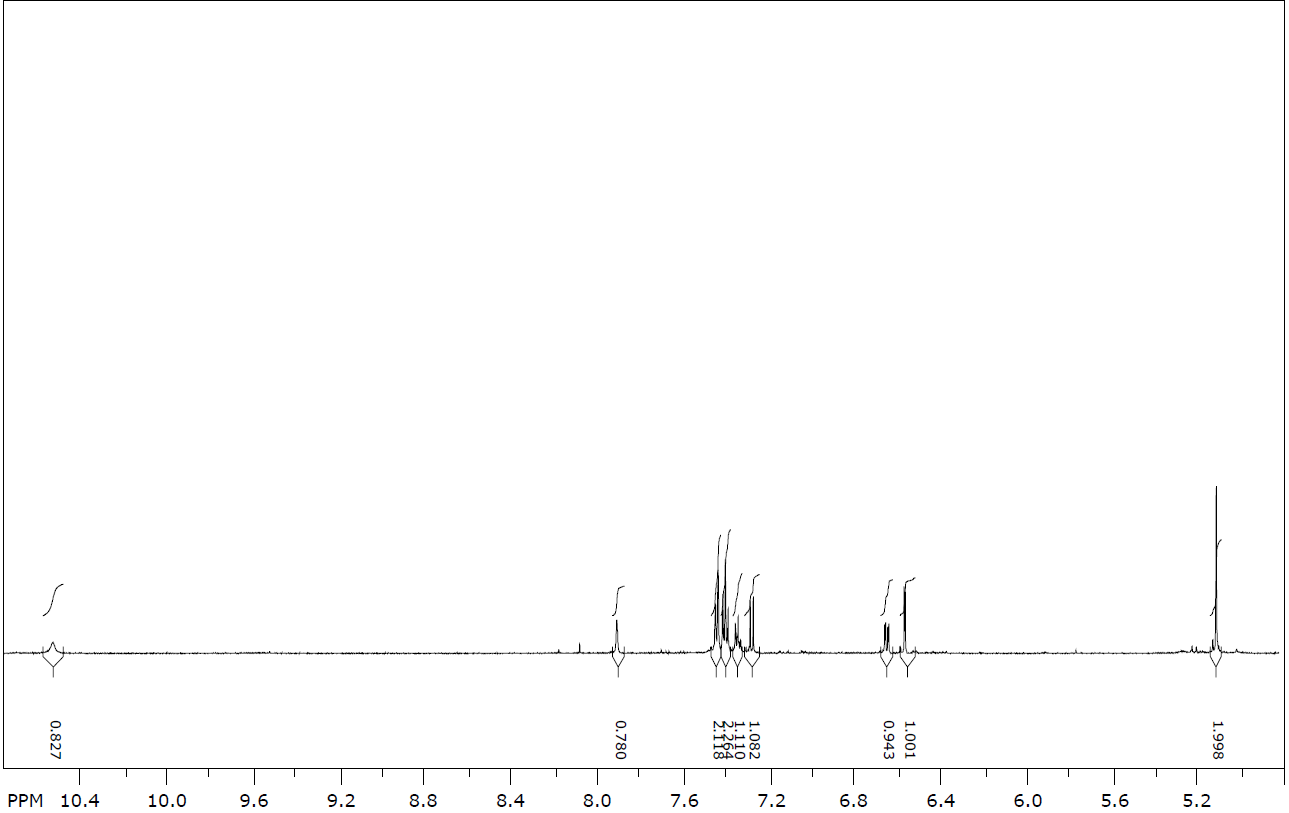


**^13^C NMR spectrum (1o)**


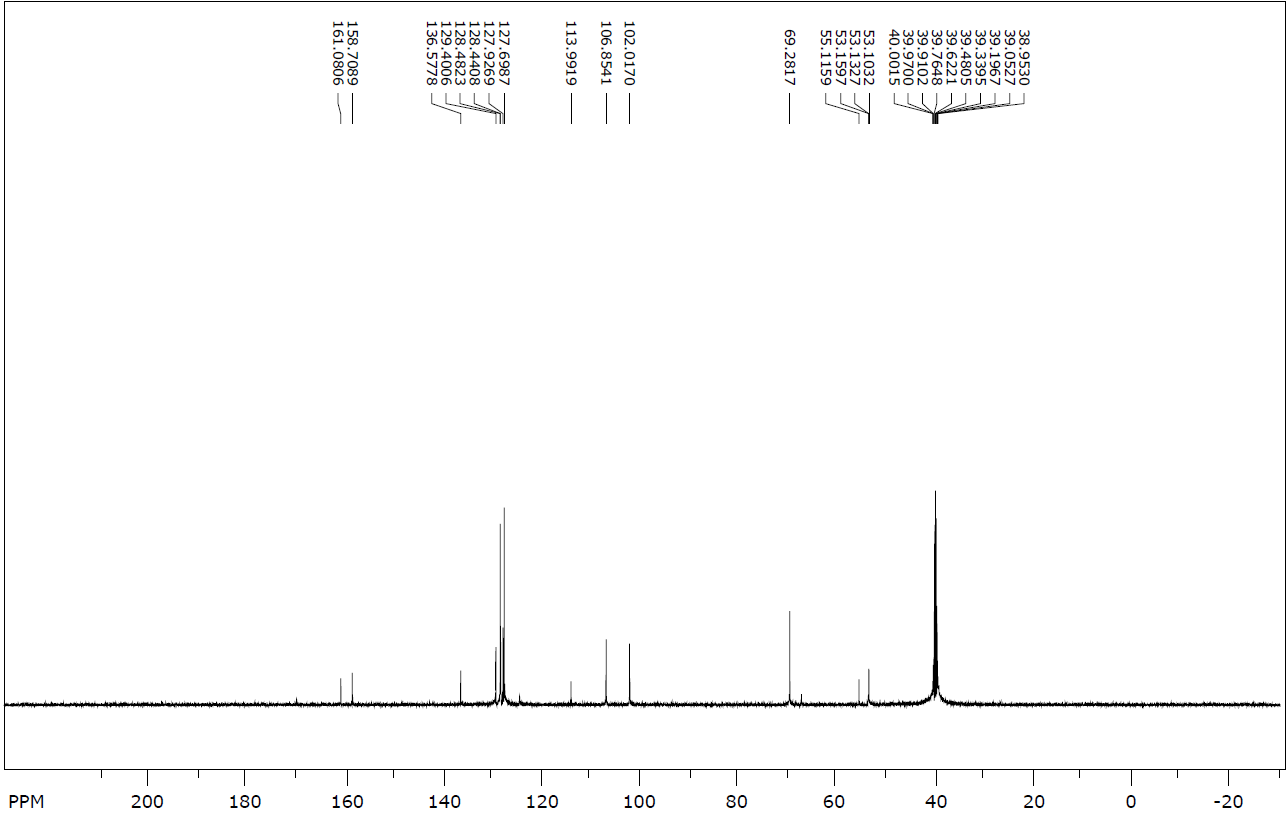


**5-(3-fluorobenzylidene)thiazolidine-2,4-dione (1p)**

Using thiazolidinedione (0.234 g, 2 mmol) and 3-fluorobenzaldehyde (212 µl, 2 mmol), in accordance with the General Procedure, the title compound **1p** was obtained (0.140 g, 31.4 % yield) as a white solid (m.p. 169 – 171 °C). **^1^H** (600 MHz) δ 12.67 (s, 1H, NH), 7.78 (s, 1H, CH), 7.58 (q, *J* = 7.98; 6.18 Hz, 1H, arom.), 7.44 (dd, *J* = 9.96; 1.98 Hz, 1H, arom.), 7.42 (d, *J* = 7.92 Hz, 1H, arom.), 7.32 (ddd, *J* = 2.16; 8.46; 8.70 Hz, 1H, arom.). **^13^C** (150 MHz) δ 167.54; 167.11; 163.03; 161.41; 135.35; 131.29; 130.31; 125.50; 119.97; 116.58.

**Mass spectrum (1p)**


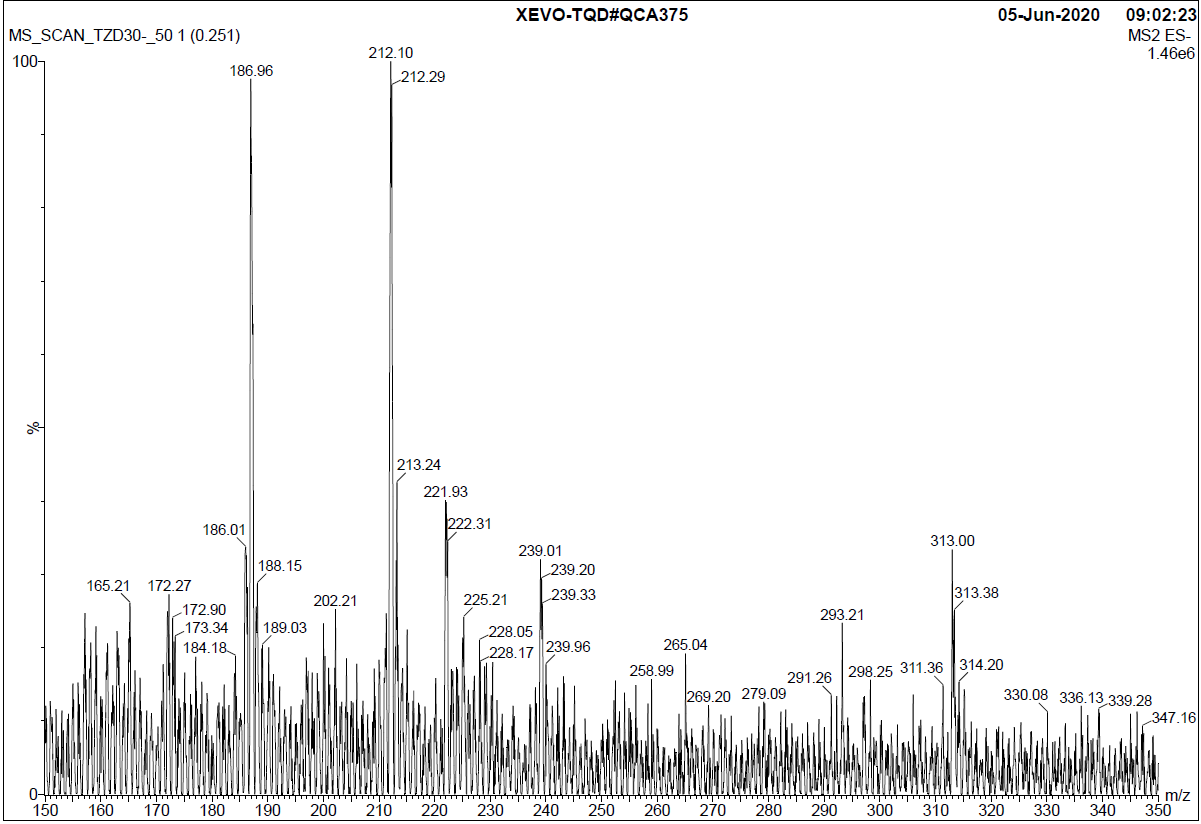


**^1^H NMR spectrum (1p)**


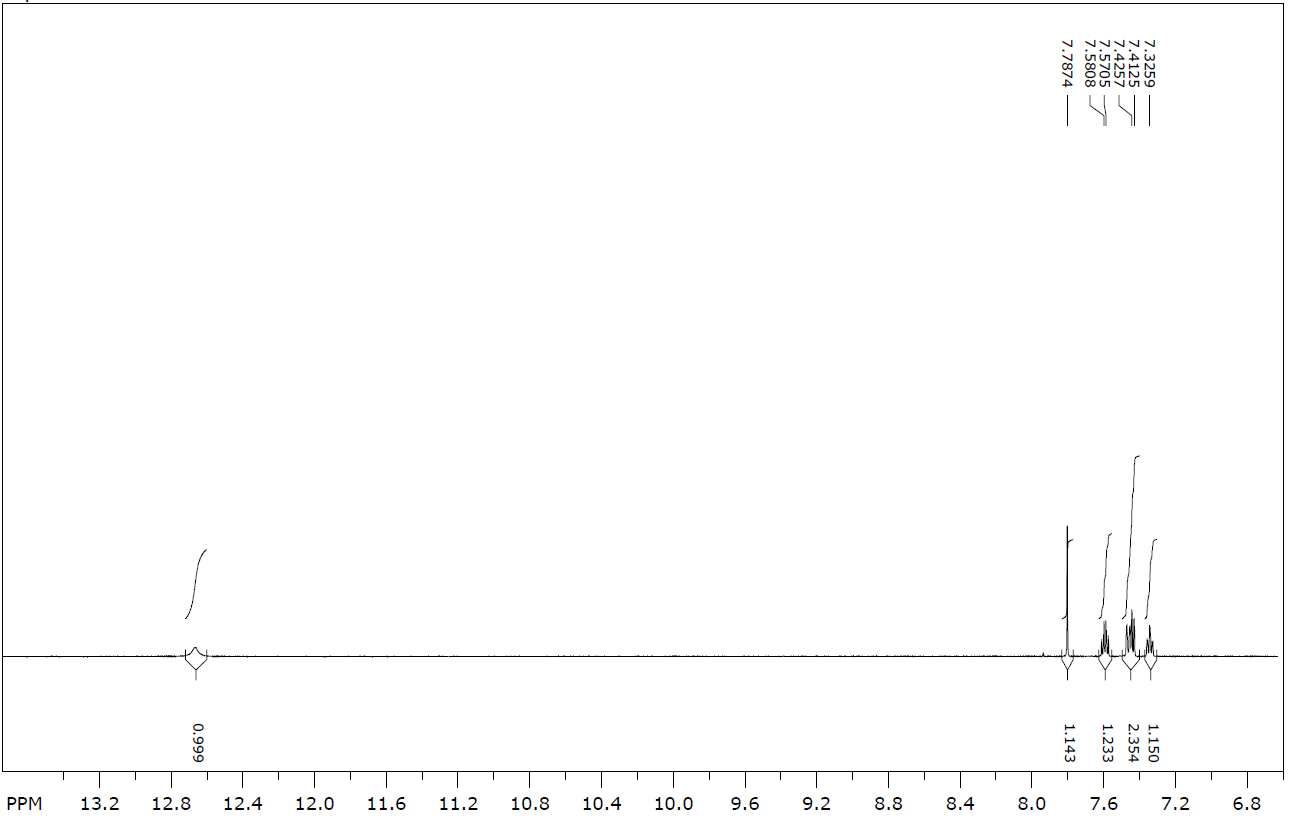


**^13^C NMR spectrum (1p)**


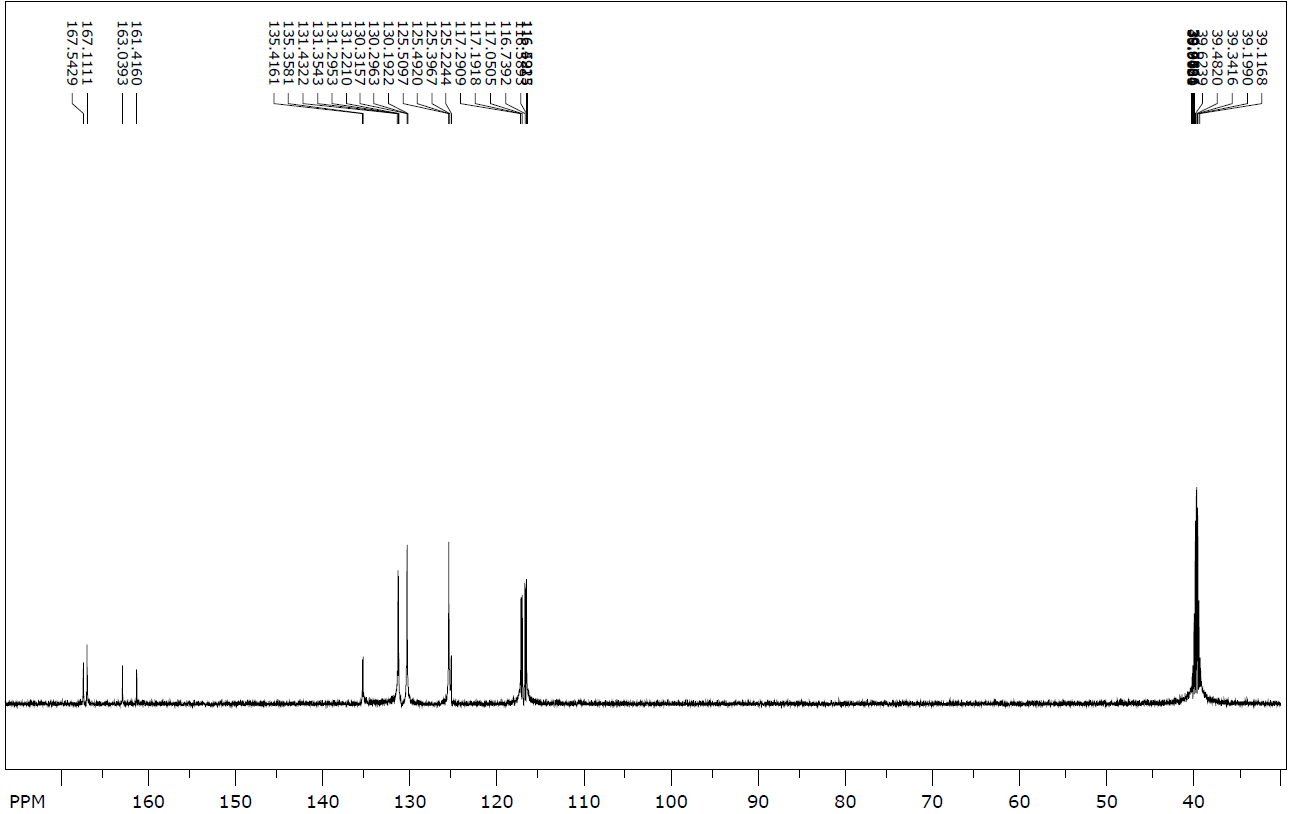


**5-(2,4-dimethoxybenzylidene)thiazolidine-2,4-dione (1q)**

Using thiazolidinedione (0.234 g, 2 mmol) and 2,4-dimethoxybenzaldehyde (0.332 g, 2 mmol), in accordance with the General Procedure, the title compound **1q** was obtained (0.467 g, 87.9 % yield) as a yellow solid (m.p. 251 – 253 °C). **^1^H** (300 MHz) δ 12.42 (s, 1H, NH), 7.90 (s, 1H, CH), 7.33 (d, *J* = 8.40 Hz, 1H, arom.), 6.69 (dd, *J* = 11.61; 2.40 Hz, 2H, arom.), 3.87 (s, 3H, OCH_3_), 3.82 (s, 3H, OCH_3_). **^13^C** (150 MHz) δ 168.04; 163.52; 160.28; 130.52; 126.92; 120.34; 114.73; 106.74; 99.09; 56.38; 56.09.

**Mass spectrum (1q)**


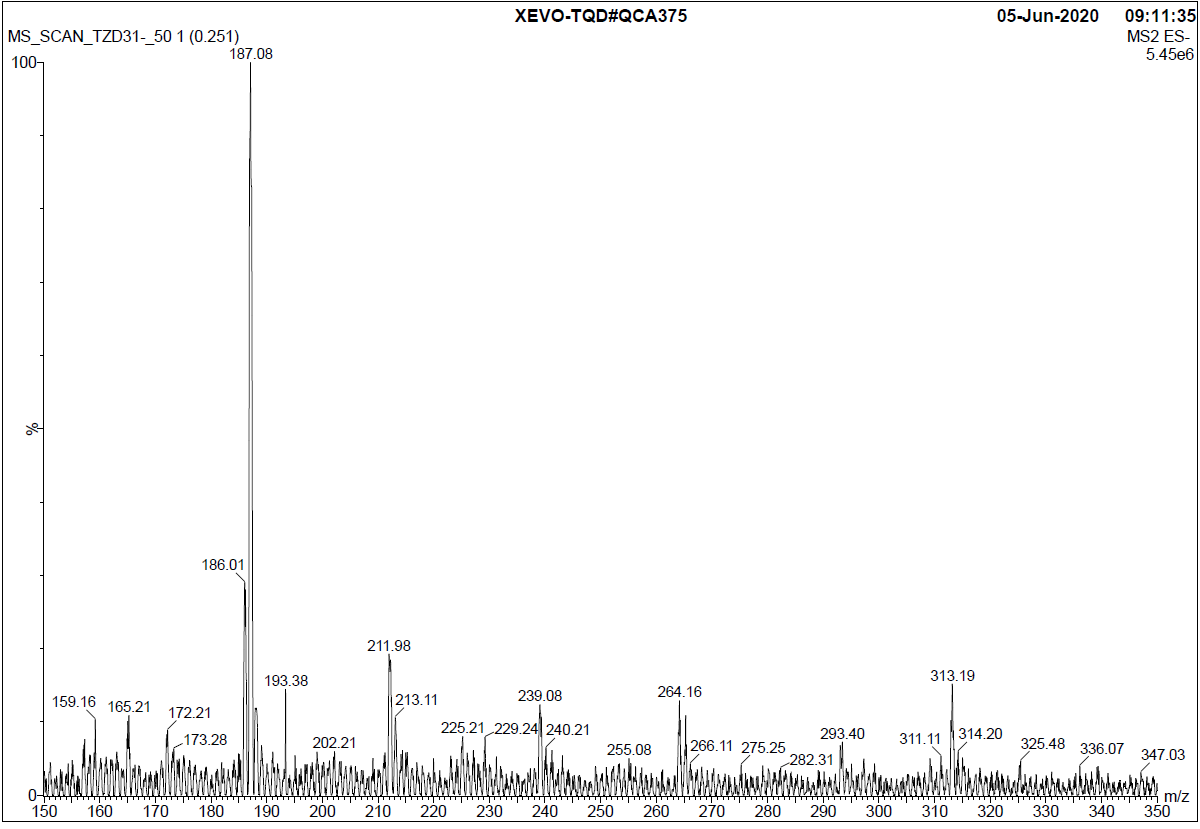


**^1^H NMR spectrum (1q)**


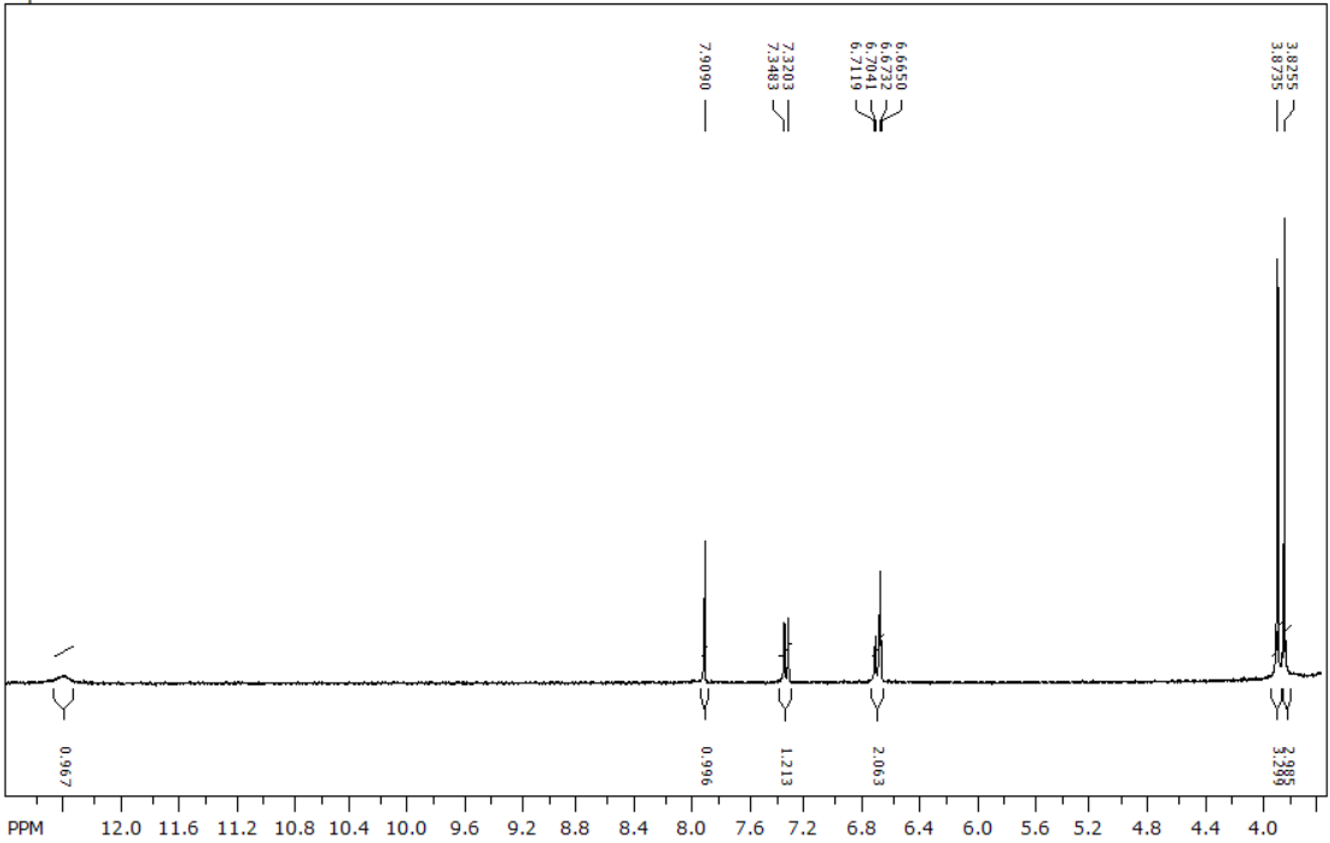


**^13^C NMR spectrum (1q)**


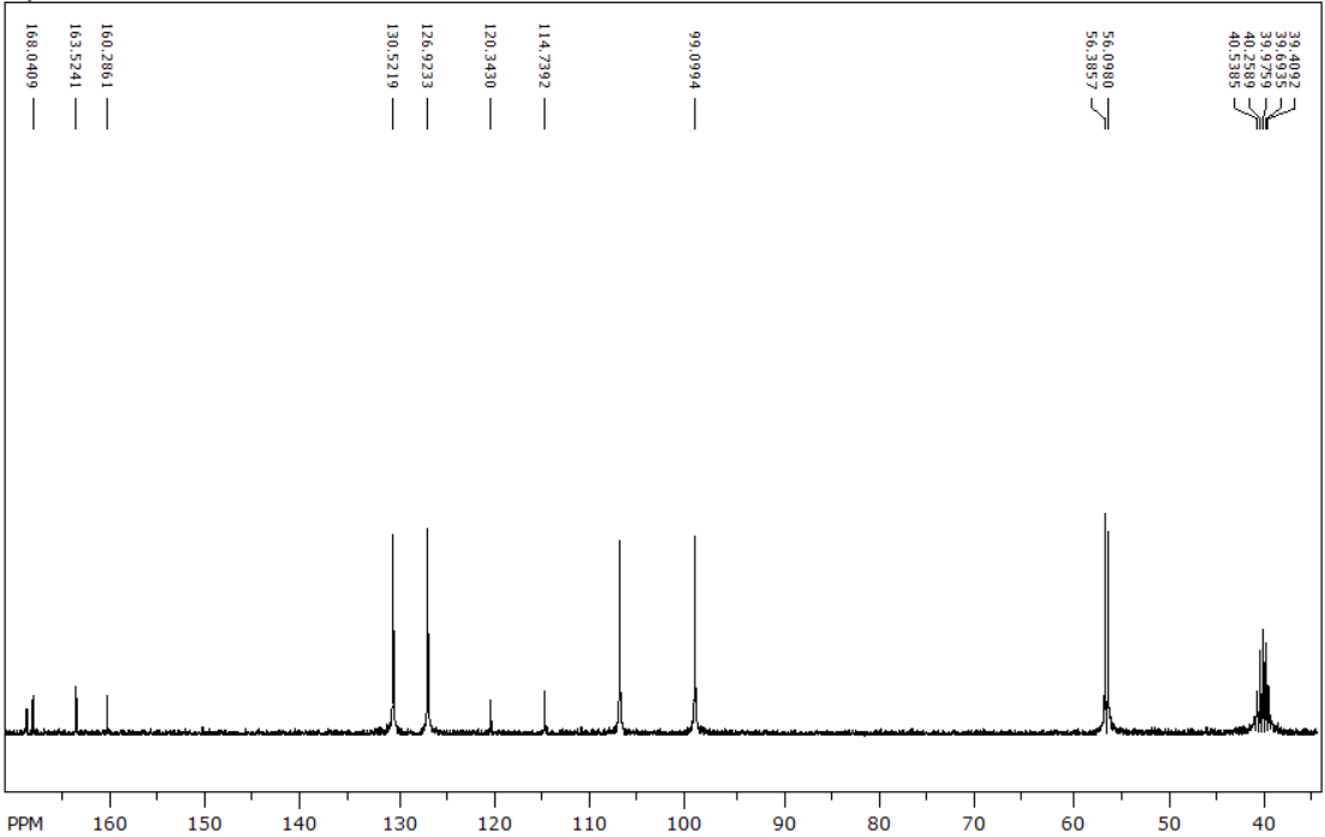


**5-benzylidenethiazolidine-2,4-dione (1r)**

Using thiazolidinedione (0.234 g, 2 mmol) and benzaldehyde (205 µl, 2 mmol), in accordance with the General Procedure, the title compound **1r** was obtained (0.134 g, 32.7 % yield) as a white solid (m.p. 251 – 254 °C). **^1^H** (600 MHz) δ 12.62 (s, 1H, NH), 7.80 (s, 1H, CH), 7.52 (m, 6H, arom.). **^13^C** (150 MHz) δ 168.33; 167.77; 133.49; 132.24; 130.87; 130.46; 129.77; 124.01.

**Mass spectrum (1r)**


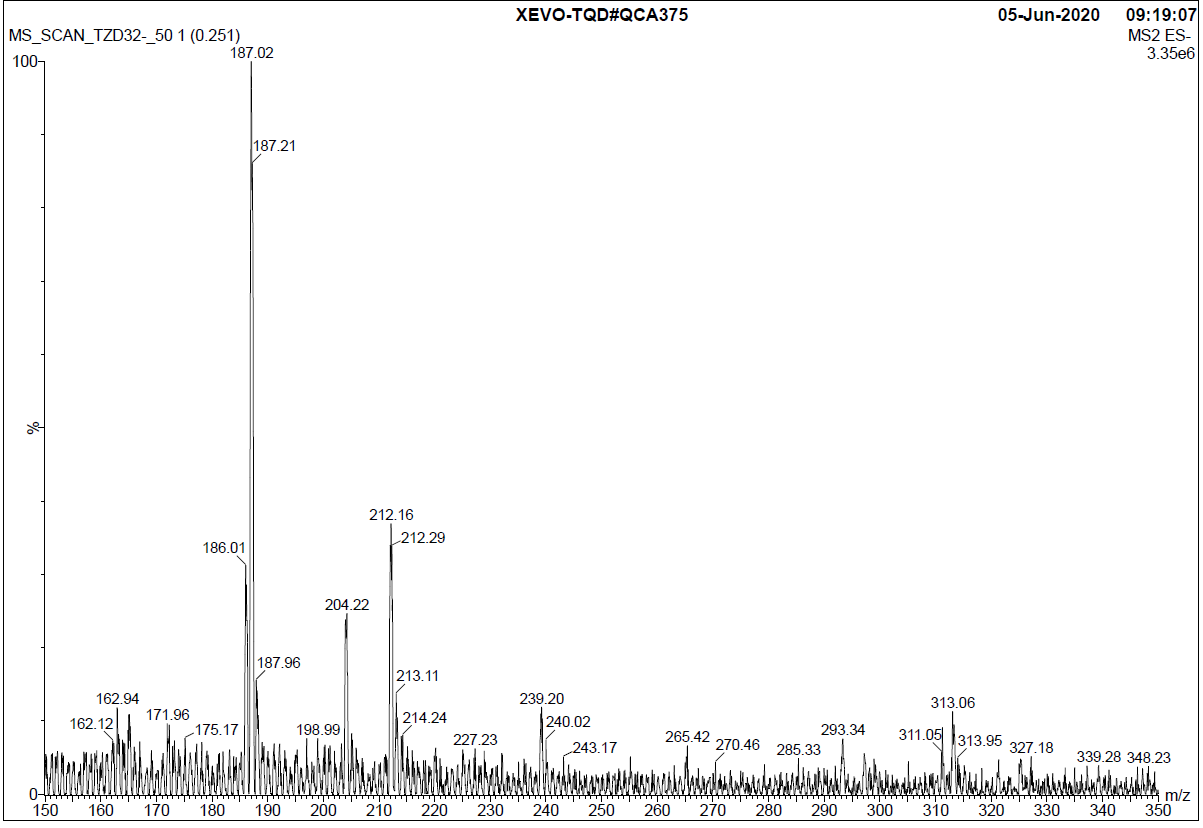


**^1^H NMR spectrum (1r)**


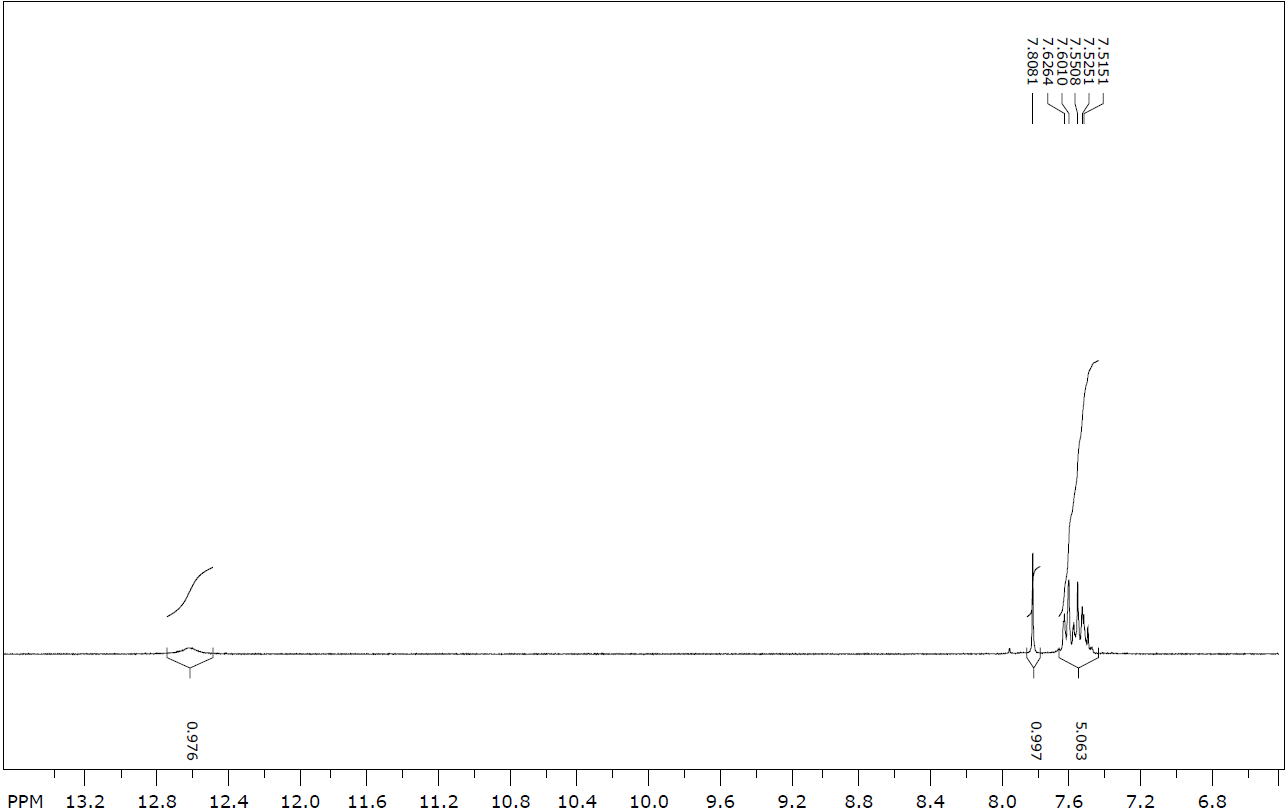


**^13^C NMR spectrum (1r)**


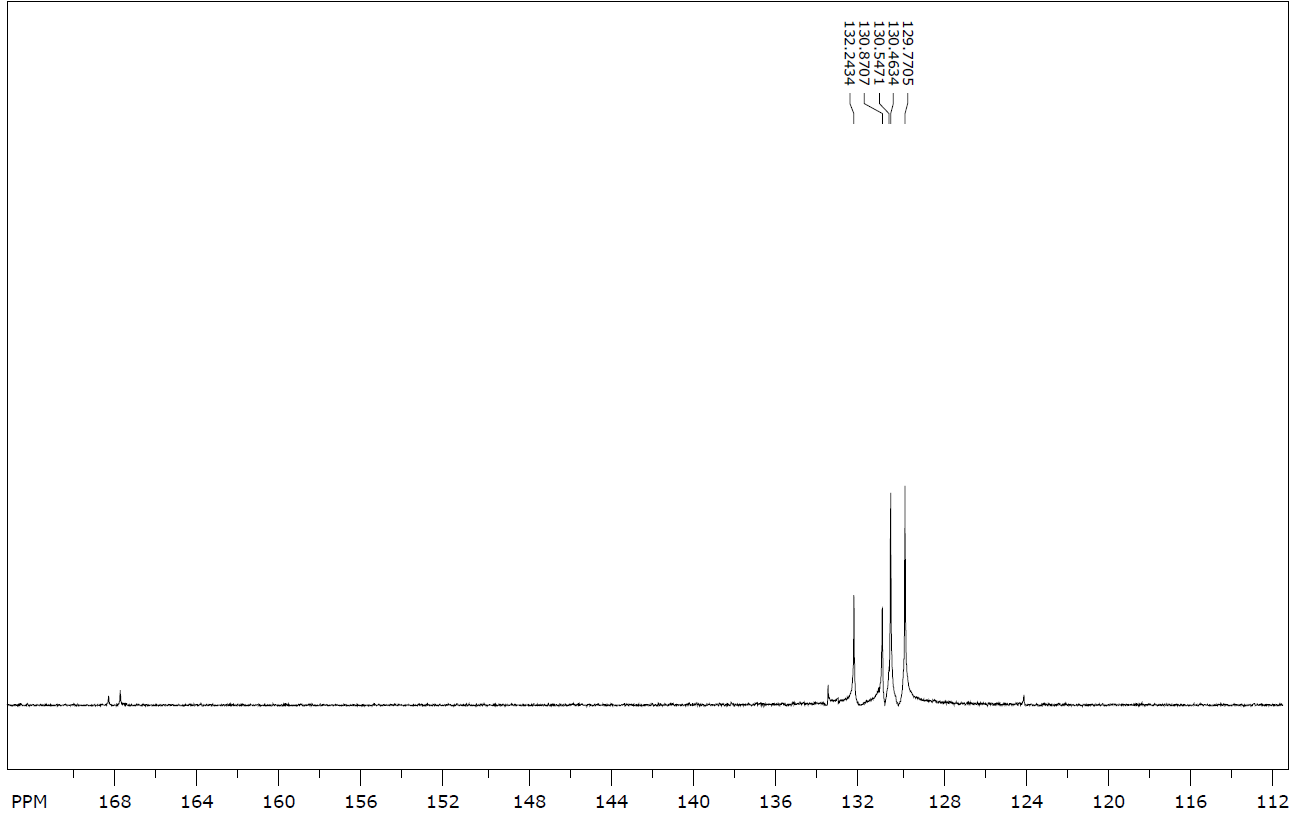


**5-((1*H*-indol-3-yl)methylene)thiazolidine-2,4-dione (1s)**

Using thiazolidinedione (0.234 g, 2 mmol) and indole-3-carboxaldehyde (0.290 g, 2 mmol), in accordance with the General Procedure, the title compound **1s** was obtained (0.439 g, 89.8 % yield) as a yellow solid (m.p.  >300 °C). **^1^H** (600 MHz) δ 12.31 (s, 1H, NH), 12.12 (s, 1H, NH), 7.88 (d, *J* = 7.86 Hz, 1H, arom.), 7.73 (d, *J* = 2.76 Hz, 1H, arom.), 7.51 (d, *J* = 8.04 Hz, 1H, arom.), 7.25 (t, *J* = 7.44 Hz, 1H, arom.), 7.19 (t, *J* = 7.38 Hz, 1H, arom.). **^13^C** (150 MHz) δ 167.23; 136.16; 128.58; 126.75; 124.41; 123.02; 121.00; 118.29; 116.20; 112.36; 110.39.

**Mass spectrum (1s)**


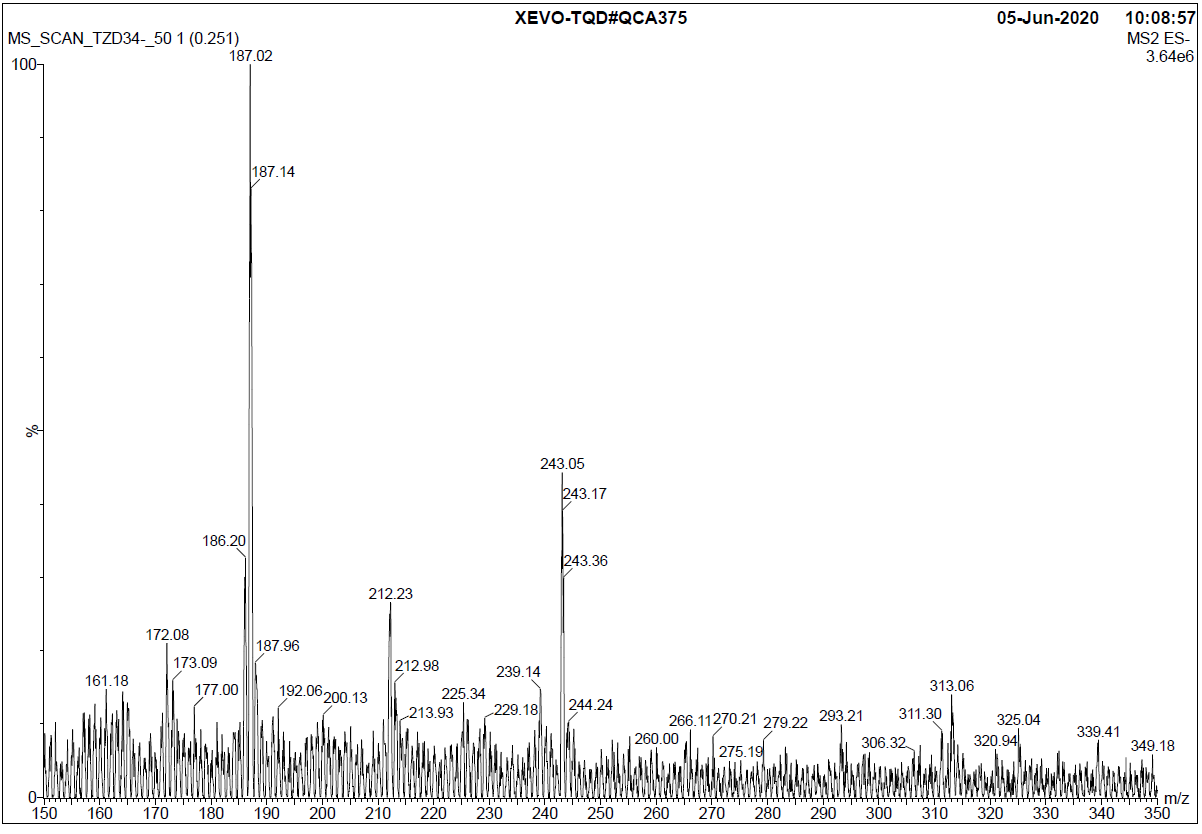


**^1^H NMR spectrum (1s)**


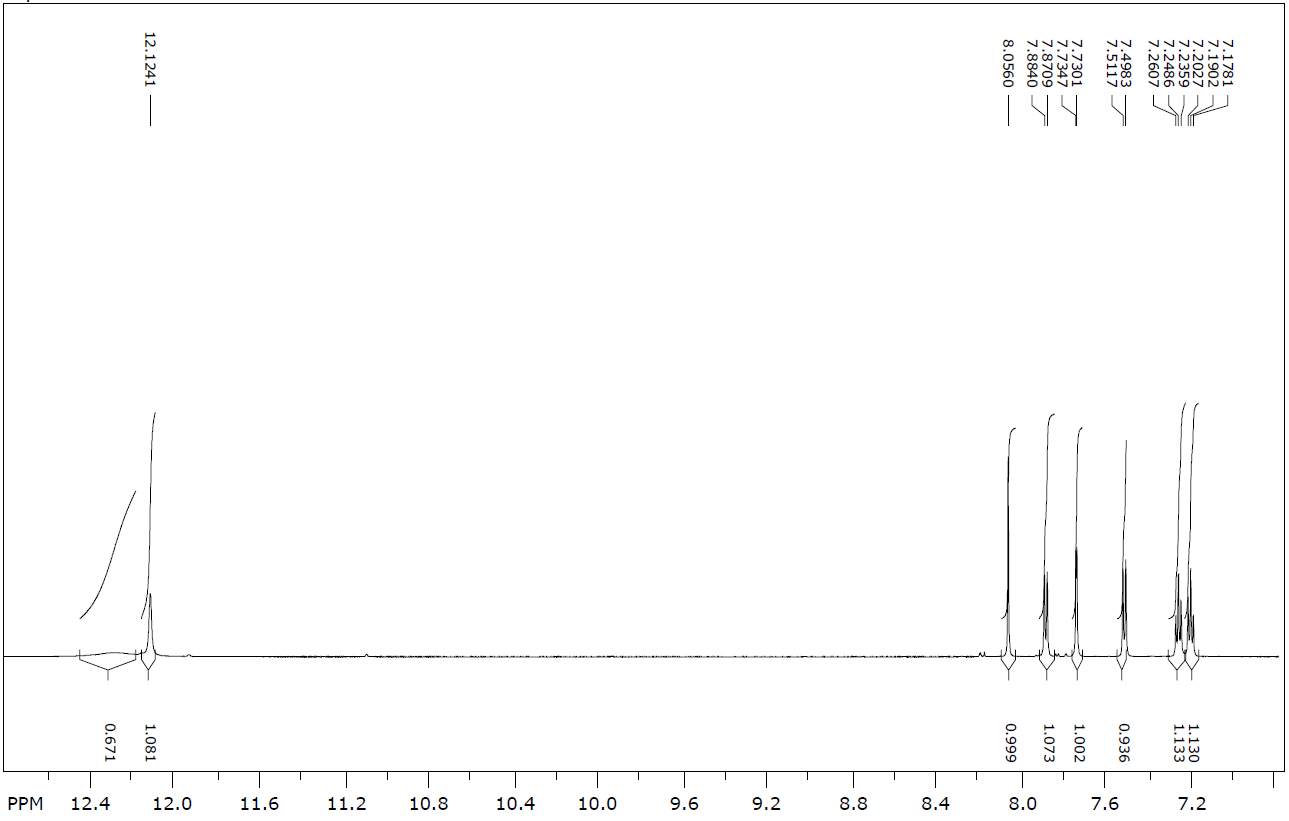


**^13^C NMR spectrum (1s)**


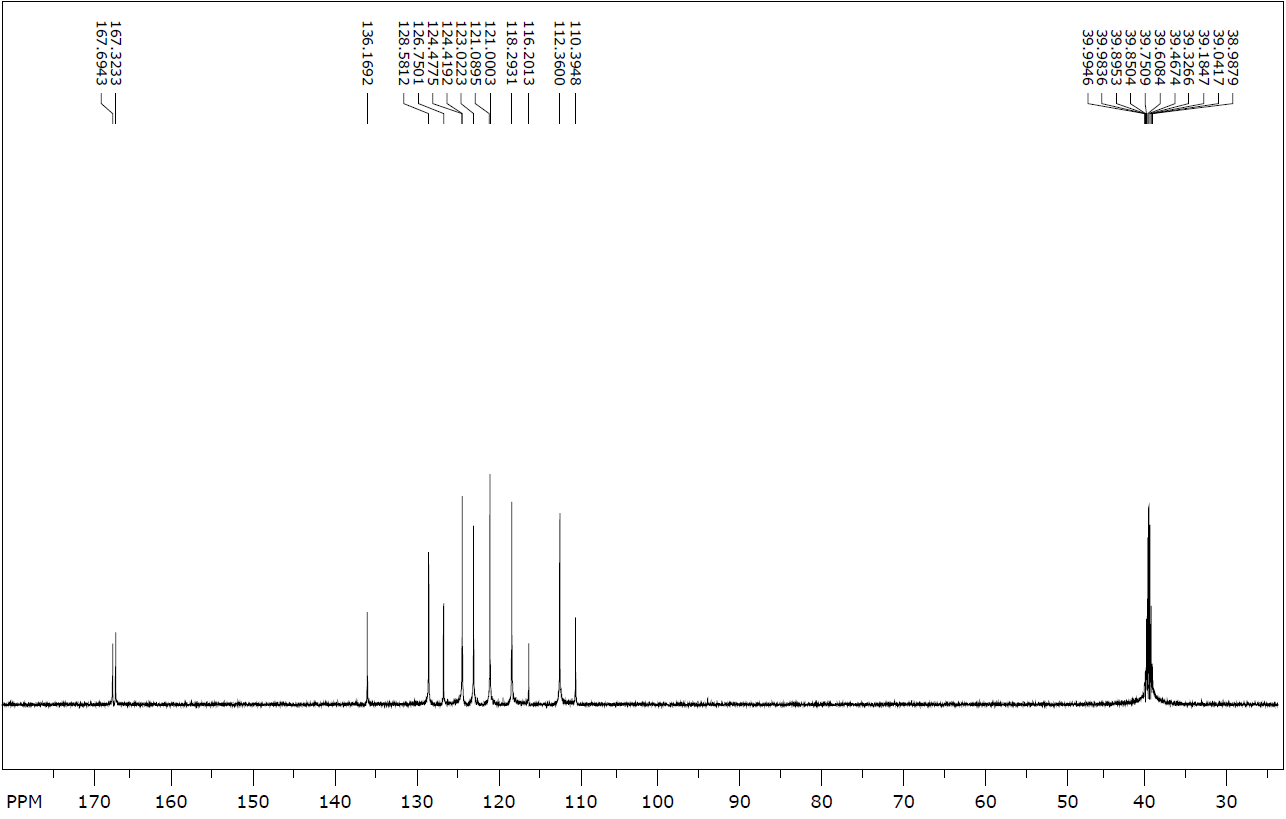

Supplement: Supplementary file 2 [file DataSheet1.docx]
